# The clinical-phenotype continuum in *DYNC1H1-*related disorders—genomic profiling and proposal for a novel classification

**DOI:** 10.1038/s10038-020-0803-1

**Published:** 2020-08-12

**Authors:** Lena-Luise Becker, Hormos Salimi Dafsari, Jens Schallner, Dalia Abdin, Michael Seifert, Florence Petit, Thomas Smol, Levinus Bok, Lance Rodan, Ingrid Krapels, Stephanie Spranger, Bernhard Weschke, Katherine Johnson, Volker Straub, Angela M. Kaindl, Nataliya Di Donato, Maja von der Hagen, Sebahattin Cirak

**Affiliations:** 1 Charité–Universitätsmedizin Berlin, Department of Neuropediatrics, Center for Chronically Sick Children, Institute of Cell- and Neurobiology, Berlin, Germany; 2 Department of Pediatrics, Faculty of Medicine and University Hospital Cologne, Center for Molecular Medicine Cologne (CMMC), University of Cologne, Cologne, Germany; 3 Department of Neuropediatrics, Medizinische Fakultät Carl Gustav Carus, Technische Universität Dresden, Dresden, Germany; 4 Carl Gustav Carus Faculty of Medicine, Institute for Clinical Genetics, Technische Universität Dresden, Dresden, Germany; 5 Human Cytogenetics Department, National Research Centre, Cairo, Egypt; 6 Carl Gustav Carus Faculty of Medicine, Institute for Medical Informatics and Biometry, Dresden, Germany; 7 University of Lille, EA 7364-RADEME, Lille, France; 8 CHU Lille, Hôpital Jeanne de Flandre, Service de Génétique Clinique, Avenue Eugène Avinée, Lille, France; 9 CHU Lille, Hôpital Jeanne de Flandre, Service de Génétique Clinique, Institut de Génétique Médicale, Lille, France; 10 Department of Pediatrics, Máxima Medical Center, Veldhoven, The Netherlands; 11 Department of Pediatrics and Neurology, Division of Genetics and Genomics, Boston Children’s Hospital, Boston, MA USA; 12 Department of Clinical Genetics Maastricht, University Medical Center, Maastricht, The Netherlands; 13 Practice for Human Genetics, Bremen, Germany; 14 John Walton Muscular Dystrophy Research Centre, Newcastle University and Newcastle Hospital NHS Foundation Trust, Newcastle upon Tyne, UK; 15 Carl Gustav Carus Faculty of Medicine, Institute for Clinical Genetics, Technische Universität Dresden, Dresden, Germany; 16 Department of Pediatrics, Faculty of Medicine and University Hospital Cologne, Center for Molecular Medicine Cologne (CMMC), Center for Rare Diseases, University of Cologne, Cologne, Germany

**Keywords:** Epilepsy, Disease genetics

## Abstract

Mutations in the cytoplasmic dynein 1 heavy chain gene (*DYNC1H1*) have been identified in rare neuromuscular (NMD) and neurodevelopmental (NDD) disorders such as spinal muscular atrophy with lower extremity dominance (SMALED) and autosomal dominant mental retardation syndrome 13 (MRD13). Phenotypes and genotypes of ten pediatric patients with pathogenic *DYNC1H1* variants were analyzed in a multi-center study. Data mining of large-scale genomic variant databases was used to investigate domain-specific vulnerability and conservation of *DYNC1H1*. We identified ten patients with nine novel mutations in the *DYNC1H1* gene. These patients exhibit a broad spectrum of clinical findings, suggesting an overlapping disease manifestation with intermixed phenotypes ranging from neuropathy (peripheral nervous system, PNS) to severe intellectual disability (central nervous system, CNS). Genomic profiling of healthy and patient variant datasets underlines the domain-specific effects of genetic variation in *DYNC1H1*, specifically on toleration towards missense variants in the linker domain. A retrospective analysis of all published mutations revealed domain-specific genotype–phenotype correlations, i.e., mutations in the dimerization domain with reductions in lower limb strength in *DYNC1H1*–NMD and motor domain with cerebral malformations in *DYNC1H1*–NDD. We highlight that the current classification into distinct disease entities does not sufficiently reflect the clinical disease manifestation that clinicians face in the diagnostic work-up of *DYNC1H1*-related disorders. We propose a novel clinical classification for *DYNC1H1*-related disorders encompassing a spectrum from *DYNC1H1–*NMD with an exclusive PNS phenotype to *DYNC1H1*–NDD with concomitant CNS involvement.

## Introduction

Mutations in the cytoplasmic dynein 1 heavy chain 1 gene (*DYNC1H1)* were first described in 2010 in autosomal dominant lower extremity-predominant spinal muscular atrophy 1 (SMALED1; MIM#158600) [1, 2]. Subsequent reports linked *DYNC1H1* mutations to neuromuscular (NMD, e.g., axonal Charcot-Marie-Tooth disease type 20 (CMT20; MIM#614228) [3, 4]) and neurodevelopmental disorders (NDD), e.g., malformations of cortical development (MCD), autosomal dominant mental retardation 13 (MRD13; MIM#614563) [5], and hereditary spastic paraplegia. Approximately 120 patients with *DYNC1H1* mutations have been published so far.

**Fig. 1:**
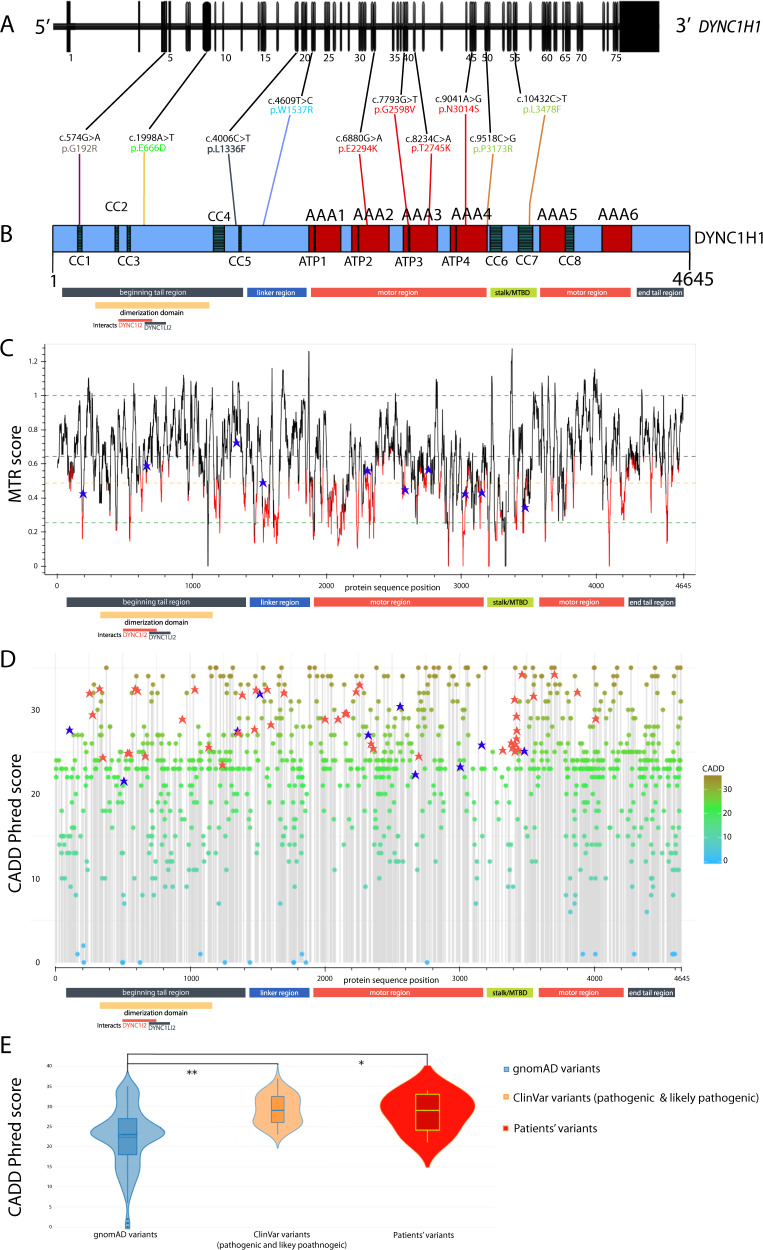
Overview of *DYNC1H1* variants identified in this study. Calculated MTR and CADD-Phred score values for the variants from the healthy population and our patient collective show that pathogenic *DYNC1H1* mutations cluster in regions of less genetic heterogeneity, specifically in highly conserved domains. **a** Ten variants in the *DYNC1H1* gene (NM_001376.5, 78 exons) identified in our patients and concomitant position in **b**. *DYNC1H1* protein structure (Q14204); pictogram with protein domains: coiled coil domain (CC, gray), ATPase associated with various cellular activities domain (AAA, red), ATP-binding region in AAA domain (ATP, dark brown), rest of protein in blue. We noted all regions (beginning tail region gray, linker region dark blue, motor region red, stalk/microtubule-binding domain green, end tail region gray) and specified the dimerization domain in yellow with interaction partners DYNC1I2 and DYNC1LI2 noted below. The mutations on protein level are presented in the above-mentioned color scheme. **c** Missense tolerant ratio (MTR) gene viewer result for *DYNC1H1* (ENST00000360184) with window size 21 (http://biosig.unimelb.edu.au/mtr-viewer/); patients’ variants are marked with blue crosses. Protein regions noted below as in **b**. **d** CADD-Phred scores of all gnomAD variants with ClinVar patient variants (marked with red asterisks) and our patient’s variants (marked with blue asterisks), score >20 indicates likely pathogenic computation, score >30 indicates pathogenic [48]. In general, CADD is a gene-level scoring for potential proxy-deleterious variants and has to be treated with caution. The linker mutations in our patient collective show amino acid exchanges with more significant changes in physicochemical properties when compared with variants from a healthy population dataset. The patients’ mutations in the motor region are found in highly conserved AAA domains with higher CADD-Phred score values. However, the pathogenic mutations from patients are in regions where allele frequencies and high CADD-Phred scores are “thinned out”. For the raw data, please see Supplementary Table 2. Protein regions noted below as in **b**. **e** Violin plot for CADD-Phred scores for variants recorded in gnomAD database (left in blue, https://gnomad.broadinstitute.org/); likely pathogenic and pathogenic variants according to ClinVar (middle in orange, https://www.ncbi.nlm.nih.gov/clinvar/), and ten patients variants (right in red), please see Supplementary Table 2 for raw data. Variance analysis (ANOVA, SigmaPlot 12.5, SYSTAT, USA) revealed significant differences between the groups “gnomAD variants” and “ClinVar variants” (**p < 0.01) as well as the groups “gnomAD variants” and “patients’ variants” in our ten patients (*p < 0.05). There was no significant difference between the groups “ClinVar variants” and “patients’ variants”

*DYNC1H1* encodes for a subunit of the cytoplasmic dynein complex. *DYNC1H1* comprises four major protein regions, i.e., tail domains (amino acid residues 1–1373 and 4222–4646), linker domain (aa 1374–1867), motor domains with AAA domains (ATPases associated with a variety of cellular activities, aa 1868–3168 and 3553–4221), and the stalk or microtubule-binding domain (MTBD, aa 3169–3552) (Fig. 1b). Recent molecular biological reports have shed light on the functional role of *DYNC1H1* in neuronal development. The dynein–dynactin complex is, together with cargo adapters (e.g., BICD2) [6–8], key for trafficking of cellular cargo to the minus-end on microtubules, for spindle pole organization, Golgi maintenance, endolysosomal processing, and nuclear positioning migration during mitosis in eukaryotic cells [6, 9, 10].

In this multi-center study, we report the clinical course of ten pediatric patients with *DYNC1H1-*associated phenotypes with nine novel pathogenic variants, highlighting the broad clinical heterogeneity of dyneinopathies and proposing a new clinical classification for *DYNC1H1*-related disorders.

## Materials and methods

### Genetic investigations

We included ten patients (P1–P10) with *DYNC1H1* (NM_001376.5) mutations in this multi-center study. All parents and/or patients gave informed consent. The study was approved by the ethics committee of the Medical Faculty, University of Cologne (17-096).

Genetic investigations of P1 via a next-generation sequencing panel (MYO-SEQ project in Newcastle University) revealed a heterozygous variant in *DYNC1H1* after various genes had been sequenced without detection of putative pathogenic variants at the time when whole-exome sequencing (WES) was not easily available [11]. Subsequently, the *DYNC1H1* variant was identified by Sanger Sequencing in the father. Later, P1 and both parents underwent trio-WES and the *DYNC1H1* variant was confirmed in P1 and his father [11].

P2 was diagnosed via a targeted next-sequencing gene panel following enrichment using highly specific Molecular Inversion Probes [12]. For P3, we performed a commercial targeted next-generation sequencing panel including coding regions of 61 NMD genes (spinal muscular atrophy and limb-girdle muscular dystrophies panel, MGZ München).

P4 received trio-WES after enrichment for the IDT xGene exome research panel followed by 2 × 150 bp sequencing with a mean target coverage of 100-fold on Illumina NextSeq500 Sequencer. Alignment (mapping to GRCh37/hg19), variant identification (SNPs and indels), variant annotation, and filtering were performed using the CLC Biomedical Genomics Workbench (Qiagen, Hilden, Germany). Variants were filtered with a focus on protein-altering variants (missense, frame-shift, splice-site, and premature stop-codons) rare or absent (de novo variants) from public databases (gnomAD and 1000 Genomes project) as previously described [13].

In P5, we performed trio-WES after enrichment with Agilent SureSelect V6 kit (Agilent, USA) on an Illumina HiSeq 4000 Sequencer (Illumina, USA) with 2 × 75 bp sequencing protocol according to the manufacturer’s best-practice protocol, a mean coverage of 85 for the patient and father and a mean coverage of 80 for the mother was achieved [14, 15]. The sequencing data were analyzed using a version of the Cologne Center for Genomics exome pipeline, version 2.20 [16]. The annotated variant lists were uploaded to the Cologne Center for Genomics Varbank (https://varbank.ccg.uni-koeln.de) database for variant filtering. Since a trio-WES was available, we additionally performed calling and filtering for de novo variants using deNovoGear [17].

For P6, the *DYNC1H1* variant in patient and parents was found by a commercial next-generation sequencing gene panel for fetal akinesia (ID 078.03, MGZ München).

In P7, we performed WES after enrichment with the NimbleGen MedExome kit (Roche NimbleGen, Basel, Switzerland) on an Illumina HiSeq 4000 Sequencing System (Illumina, San Diego, CA, USA) with 2 × 150 bp sequencing protocol according to the manufacturer’s best-practice protocol. The variant calling and filtering pipeline were described earlier elsewhere [14].

In P8, trio-WES was performed after enrichment using the SOLiD‐optimized SureSelect All Human Exon Kit (50 Mb; Agilent, Technologies, Santa Clara, CA, USA), followed by sequencing on 5500XL sequencers (Life Technologies, Carlsbad, CA, USA). Quality control parameters were checked throughout the laboratory workflow. Sequence reads were aligned to the human genome (hg19) using Lifescope v2.1 (Life Technologies), followed by variant calling on the aligned sequence. Variants were annotated using a custom analysis pipeline. Samples were automatically checked for quality (e.g., median coverage). For further information on the sequencing procedure, please see previously published data [18].

In P9, we performed WES with Agilent Clinical Research Exome Kit on an Illumina HiSeq 2000 with 2 × 100 bp reads, mean depth of coverage of 103×, and quality threshold of 95.9% (percentage of XomeDx which is covered by at least ten sequence reads/10× coverage). The data were aligned to reference NM sequence based on GRCh37/hg19 and analyzed for sequence variants using a custom-developed analysis tool (Xome Analyzer).

In P10, we performed a next-generation sequencing panel targeting *PAFAH1B1, KIF5C, KIF2A, TUBG1, CRADD,* and *DYNC1H1*, after using enrichment with the Agilent in-solution hybridization technology followed by sequencing on an Illumina HiSeq Sequencing system (Illumina, USA). The analysis included a next-generation sequencing-based copy number variant calling and analysis. The panel was supplemented with an MLPA-based deletion- and duplication analyses for *PAFAH1B1*.

We performed dideoxy seqeucning for confirmation of all the patients’ *DYNC1H1* variants and for further co-segregation analyses, except for P1 and P10.

All variants were scored based on the classification by the standards and guidelines of the American College of Medical Genetics and Genomics—American College of Molecular Pathology (ACMG) for the interpretation of variants [19].

### Genotype–phenotype analyses including retrospective literature analysis

To further characterize the phenotype of patients with mutations in *DYNC1H1*, we searched via “Pubmed” for publications of patients with mutations in *DYNC1H1* from 01.01.2010 to 01.01.2020 and identified 24 publications with 120 patients [1, 3–5, 20–38]. We used IBM SPSS Statistics 25 (IBM Corp. Released 2017, Armonk, NY) to generate a database of all patients including our ten patients (*n* = 130) and assigned each patient for 12 clinical manifestations (upper and lower limb strength, intellectual disability (ID), behavior, seizures, MRI abnormalities including pachygyria, heterotopias, enlarged ventricles, hypoplasia of corpus callosum, of the brain stem, and the cerebellum) either a physiological phenotype “0” or an abnormal phenotype “1”. Where information was not available or not specifically mentioned in the publications, we did not assign a number and marked it as missing. For an overview of all patients included in the analyses, please see Supplementary Table 1.

### Statistical analyses

We performed further in silico and genomic analysis [39] for all variants, checking in population databases such as gnomAD (http://gnomad.broadinstitute.org), 1000 Genomes [40], GME [41], and ExAC browser (http://exac.broadinstitute.org/). To evaluate the pathologic potential of the resulting variants, we used MutationTaster (http://www.mutationtaster.org/), SIFT (http://sift.jcvi.org/), PolyPhen2 (http://genetics.bwh.harvard.edu/pph2/), GERP++ [42], REVEL [43], CADD-Phred (https://cadd.gs.washington.edu/), and MutPred2 (http://mutpred.mutdb.org/).

**Fig. 2:**
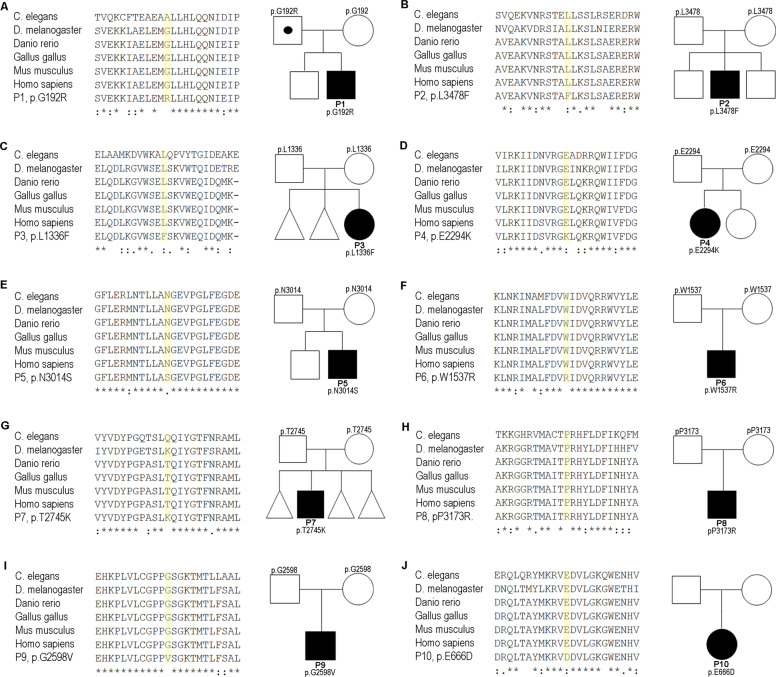
Pedigrees of P1–10 and multi-species sequence alignment of the mutated proteins. **a** Pedigree and multi-species sequence alignment of P1, to note, father is also heterozygous but does not show any symptoms. **b**–**j** Multi-species sequence alignment and pedigrees of P2–10. Multi-species sequence alignment was performed using Clustal Omega (Caenorhabditis elegans, NP_491363.1; Drosophila melanogaster, NP_001261430.1; Danio rerio, NP_001036210.1; Gallus gallus, XP_015143281.1; Mus musculus, NP_084514.2; Homo sapiens, NP_001367.2). □ male, not affected; ○, female, not affected; with dot, carrier; ■, male, affected; ●, female, affected; *, fully conserved: conserved between groups of strongly similar properties; conservation between groups of weakly similar properties

The missense tolerance ratio (MTR, Fig. 1c) calculates the number of observed missense DNA variations relative to the number of all observed (missense and synonymous) single base variants, then divided by the number of expected missense mutations relative to the number of all possible variations in that segment [44]. To evaluate the evolutionary conservation, we performed multiple sequence alignment for all patients using Clustal Omega (https://www.ebi.ac.uk/Tools/msa/clustalo/) (Fig. 2).

In order to judge the pathogenicity on a domain-specific protein level and investigate the domain-specific vulnerability and conservation of *DYNC1H1*, we analyzed datasets on *DYNC1H1* variants in a healthy population (gnomAD database) with a patient collective. We performed two-factor analysis of variances (ANOVA, SigmaPlot 12.5, SYSTAT, USA) for CADD-Phred score values and MTR score values between the groups “reports” (healthy subjects vs patients) and the groups “protein regions” (tail vs linker vs motor vs MTBD). For these analyses, we pooled the ten patients from our collective with pathogenic and likely pathogenic *DYNC1H1* mutation reports from the ClinVar database (Supplementary Table 2). For all statistical analyses, we performed one-factor or two-factor ANOVA as specified in the text, and we report significant differences with a *p* value below 0.05 or lower.

For statistical analyses of the genotype–phenotype analyses, we performed Pearson’s Chi-Square test, Lambda, Phi, and Cramer V test (Supplementary Table 3) to correlate the phenotype to the localization of the mutations categorized by the domain (beginning tail, dimerization, linker, motor domain). We plotted the results with a balloon plot using R 3.6.3 GUI 1.70 El Capitan build (7735) for MacOS and the packages ggpubr, ggplot2, and magrittr (Fig. 4b, c).

## Results

### Clinical findings

We report ten pediatric patients (P1–P10) with overlapping *DYNC1H1*-associated phenotypes, onset in infancy, and little to no disease progression (Table 1, Fig. 2). Motor development was delayed in all patients except for P10. Eight out of ten patients had muscular weakness and atrophy predominantly of the lower limbs (P1–P8) with “crouching” movements and three patients exhibited weakness also of the upper limbs (P1, P6, and P7). Deep tendon reflexes (DTR) were normal in three patients (P4, P9, and P10); all other patients had reduced DTR in the lower limb but normal DTR in the upper limb.

**Table 1:** Clinical heterogeneity between ten patients (P1–P10) with *DYNC1H1* mutations

Pat.	*DYNC1H1* variant	Inheritance	Onset (progression)	Eye abnorm.	Spine/pelvis deformity	Orthopedic abnorm. (contractures)	Muscle strength	DTR	Motor develop.	Gait	Cognition/behavioral disorder	Seizures	NCS	Cranial MRI/CT	Organ malformation
P1	c.574G > A p.Gly192Arg	AD het	Infancy (no)	No	Hip dysplasia lumbar hyperlordosis	Pes cavus	− − LE − UE − Hip (pos. Gowers sign)	+ UL − − LL	Delayed	Broad-based waddling	Aggressive behavior, ADHS, mild learning impairment	No	Normal	Normal	Mild aortic valve insufficiency, dilatative uropathy III°
P2	c.10432C > T p.Leu3478Phe	de novo het	Infancy (slow)	Strabismus	No	Flat feet, pes equinus (hips, knees)	− − PLE − Hip (neg. Gowers sign)	+ UL − − LL	Delayed	Broad-based waddling	Severe ID	Focal epilepsy	Signs of left axonal loss of sensory and motor neurons	Enlarged right ventricle, white matter hypomyelination	x
P3	c.4006C > T p.Leu1336Phe	de novo het	Infancy (slow)	No	Lumbar hyperlordosis	Pes equinus	− − PLE	+ UL − − LL	Delayed	Broad-based waddling	Mild learning impairment	No	Normal	Partially CC hypoplasia, clumsy ventricle; syringomyelia	Bicuspid aortic valve
P4	c.6880G > A p.Glu2294Lys	de novo het	Infancy (no)	No	No	Pes equinovarus	− − PLE (pos. Gowers sign)	+	Severely delayed	Broad-based waddling	Severe ID	Focal epilepsy	x	Diffuse pachygyria with thick cortex; frontal, perisylvian predominance	x
P5	c.9041A > G p.Asn3014Ser	de novo het	Infancy (slow)	Bilateral congenital cataract	No	No	− − LE	+ UL − − LL	Severely delayed	x	Global develop. delay	No	Signs of left axonal loss of sensory and motor neurons	Arachnoidal cysts, unspecific corticomedullary junction lesions, periventricular hyperintensities, cortical atrophy	x
P6	c.4609T > C, p.Trp1537Arg	de novo, het	Infancy (slow)	Convergent strabismus	Merging of dens tip and atlas anterior arch	Pes adductus (hips)	− PLE − UE	+ UL − − LL	Delayed	Broad-based waddling	Speech delay	No	x	Normal	x
P7	c.8234C > A p.Thr2745Lys	de novo het	Infancy (slow)	No	No	No	− − LE − UE	+ UL − − LL	Delayed	x	Motor stereotype, attention deficit	No	x	Cerebellum dysplasia	Congenital anterior diaphragmatic hernia
P8	c.9518C > G p.Pro3173Arg	de novo het	Infancy (slow)	Bilateral cataract (onset 9 month)	No	No	− − LE	+ UL − − LL	Delayed	Broad-based waddling with support	Global develop. delay, ID	Focal epilepsy	Signs of left axonal loss of sensory and motor neurons	Bifrontal PMG, periventricular white matter abnormalities, ventriculomegaly	x
P9	c.7793G > T p.Gly2598Val	de novo het	Infancy (no)	Bilateral cataracts (onset 9 months)	No	No	Full strength	+	Delayed	Minor imbalance	Global develop. delay, ADHD	No (but ETPs in EEG)	x	CC hypoplasia, periatrial white matter volume loss, heterotopia	x
P10	c.1998A > T p.Glu666Asp	de novo het	Infancy (no)	Amblyopia	No	No	Full strength	+	Normal	Minor imbalance	Global develop. delay, severe speech impairment	Focal epilepsy	x	Bifrontal pachygyria, heterotopia, pons hypoplasia	Accessory spleen

*ADHD* attention deficit hyperactivity disorder, *abnorm.* abnormalities, *CNS* central nervous system, *CC* corpus callosum, *develop* development, *DLL* distal lower limb, *DTR* deep tendon reflexes, *EEG* electroencephalography, *ETP* epileptic potentials, *ID* intellectual disability, *LE* lower extremity, *MCD* malformation of cortical development, *NCS* nerve conduction studies, *sec.* secondary, *PLE* proximal lower extremity, *PMG* polymicrogyria, *PNS* the peripheral nervous system, *UE* upper extremity, *−* mildly reduced, *−−* severely reduced, *+* normal, *x* not performed/documented

Feet deformities were present in five patients (pes cavus: P1; pes equinus: P2, P3; pes equinovatus: P4, pes adductus: P6), contractures in two patients (hips: P2, P6; knees: P2), spine deformities in three patients (lumbar hyperlordosis: P1, P3; merging of dens tip and atlas anterior arch: P6), and one patient displayed with hip dysplasia (P1). Gait was assessed in eight patients and abnormal in all with the majority having a broad-based waddling gait (P1–P4, P6, and P8) and two with only minor imbalance (P9 and P10). All patients had varying degrees of neurodevelopmental delay, ranging from mild learning impairment, via speech developmental delay (P6, P10) to global developmental delay (P5, P10) or mild (P3, P8) or severe ID (P4).

Information about nerve conduction velocities were available in five patients, with three patients having signs of axonal impairment of sensory and motor neurons (P2, P5, and P7).

**Fig. 3:**
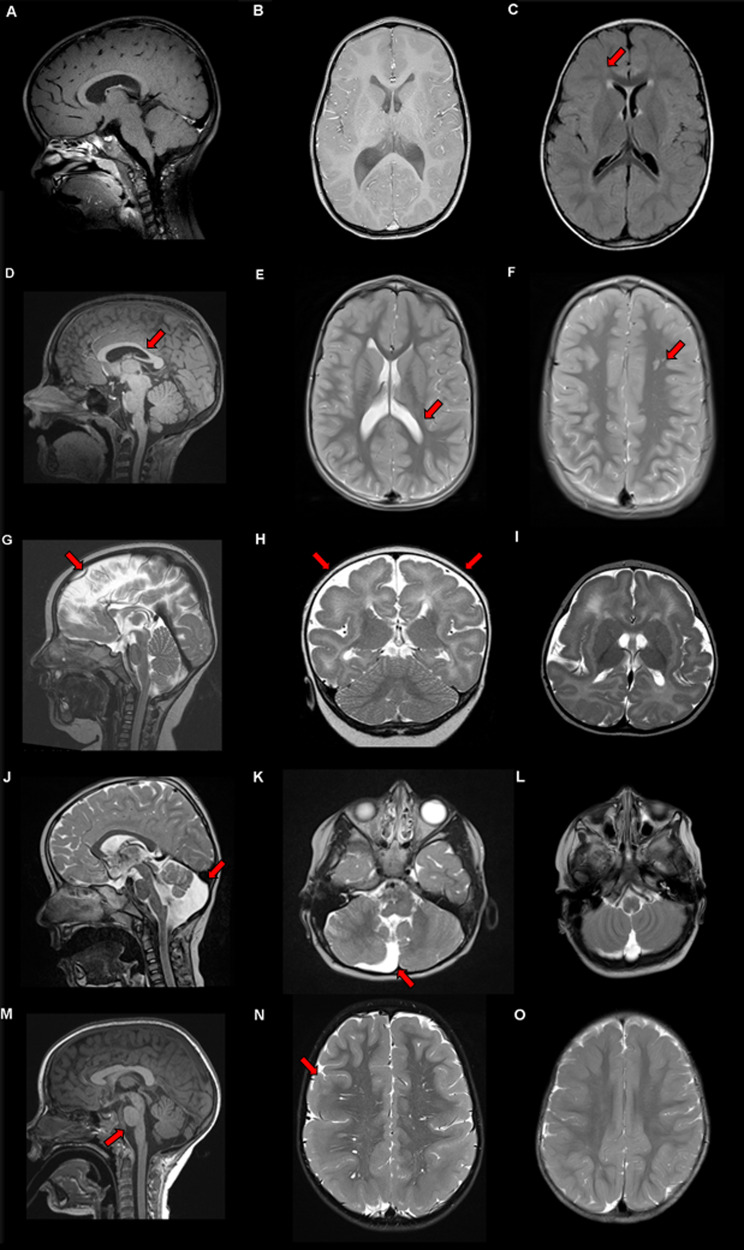
Brain abnormalities found in patients with mutations in *DYNC1H1*. The arrow in the figure points out the brain abnormality described in the figure legend. **a** Sagittal and **b** axial T1 weighted image of P6 at the age of 3 years showing normal brain anatomy. **c** Axial FLAIR sequences in P5 at 23 months-of-age showing periventricular lesions and cortical atrophy. **d** Sagittal T1 weighted MRI of P9 at the age of 7 years, revealed thinning of posterior body of corpus callosum. **e** Axial T2-weighted MRI of P9, revealed enlarged posterior horns of lateral ventricles and periatrial white matter volume loss. **f** Axial T2-weighted MRI of P9, indicating cortical heterotopia in left frontal deep white matter. **g** Sagittal, **h** coronal, and **i** axial T2-weighted MRI of P4 at the age of 7 months-of-age showing diffuse pachygyria with cortical thickness >10 mm with frontal and perisylvian predominance. **j** Sagittal and **k** transversal T2-weighted MRI of P7 at the age of 4 years showing an isolated dysplasia of the right cerebellum and cerebellar vermis with an enlargement of the fourth ventricle on the left and notably without the involvement of the pons or mesencephalon. **l** Transversal T2-weighted MRI of P5 at 23 months-of-age showing in contrast to **k** no vermis or ventricle enlargement. **m** Sagittal T2-weighted MRI of P10 at the age of 4 years showing brain stem hypoplasia. **n** Axial T2-weighted MRI of P10 showing bilateral temporal and parietal pachygyria reaching until dorsal part of the frontal lobe. **o** Axial T2-weighted MRI of P5 at 23 months-of-age showing cortical brain atrophy without pahygyria

Early onset epilepsy was diagnosed in P2, P4, P8, and P10 at a median age of 27 months (range 11–48 months). Most patients remained seizure free with single or combined anticonvulsive medication, except the therapy-refractory course of P4, where levetiracetam and ketogenic diet merely reduced seizure frequency (Table 1). Magnetic resonance imaging (MRI) revealed brain anomalies in all patients except P1 and P6, with ventricle abnormalities (P2, P3, and P7), pachygyria (P4 and P10), corpus callosum hypoplasia (P3 and P9), pons hypoplasia (P10), and cerebellar dysplasia (P7) (Table 1, Fig. 3).

Furthermore, we identified extra nervous system symptoms, irrespective of the predominant clinical presentation, i.e., (i) facial dysmorphism, macrocephaly (P2), (ii) ophthalmologic anomalies (strabism, (P2, P6), amblyopia (P10), congenital cataracts (P5, P8, and P9)), (iii) osteocutaneous anomalies (e.g., prominent calcanei, cutis laxa, P5), (iv) syringomyelia (P3), (v) bicuspid aortic valve without stenosis but mild insufficiency, (vi) mild aortic valve insufficiency (P1), (vii) congenital diaphragmatic hernia (P7), (viii) dilatative uropathy (P1), and (ix) an accessory spleen (P10) (Table 1). The father of P1, who carries the same variant, has no definite signs or symptoms of a NMD, but he has never been athletic and has developed bilateral hip anthropathy around the age of 40 years. A simular case of a family member with the same mutation as a severely affected individual has been described in the literature before [37].

### Domain-specific review of genotype–phenotype correlations in the literature

**Fig. 4:**
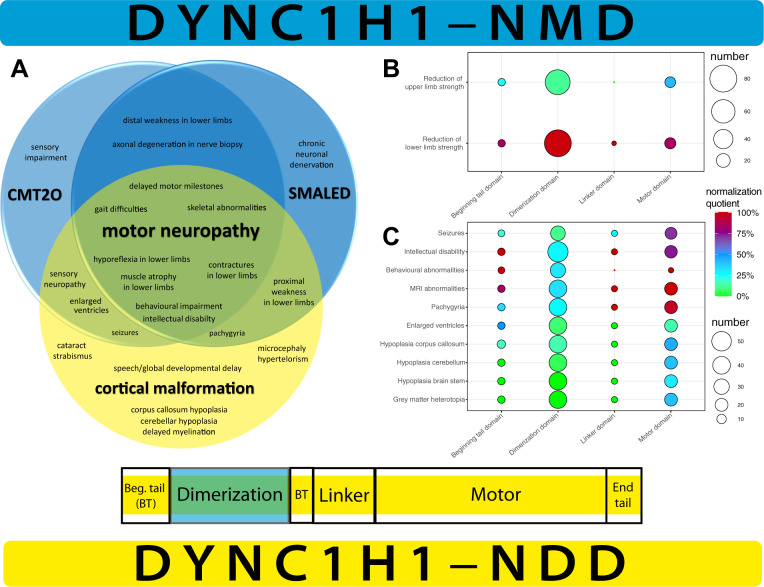
Overview of overlapping clinical disease manifestation of *DYNC1H1*-associated disorders and domain-specific presentation of genotype–phenotype correlation based on literature review. **a** On the left, Venn diagram of the recorded symptoms in patients with each of the three known entities associated with *DYNC1H1* mutations and the overlap of phenotypes in *DYNC1H1*-associated disorders: Charcot-Marie-Tooth disease Type 20 (CMT20), lower extremity-predominant spinal muscular atrophy (SMALED), and cortical malformations. The symptoms were taken from an extensive PubMed literature search (“dync1h1”, with each “motor neuropathy”, “CMT20”, “charcot-marie-tooth”, “SMALED”, “spinal muscular atrophy”, “malformation”, “MRD13”, “mental retardation”). Specifically, neuromuscular symptoms as in CMT20 and SMALED were mostly observed in patients with mutations in the dimerization domain and cortical malformation was mostly observed in motor domain mutations. On the right, a simplified overview of the protein model from Fig. 1b. **b** Balloon plot for symptoms “reduction of upper limb strength” and “reduction of lower limb strength” recorded in the literature search with mutations in the beginning tail, dimerization, linker, and motor domains. The size of patient groups denoted with the size of circles on the right (smallest circle 20, biggest circle 80). The calculated normalization quotient (from green to blue to red, on the right) from Pearson’s chi-square test as described in methods revealed clustering of reductions of lower limb strength with preserved upper limb strength in the dimerization domain. **c** Balloon plot for symptoms “seizures”, “intellectual disability”, “behavioral abnormalities”, “MRI abnormalities” in general, “pachygyria”, “enlarged ventricles”, “hypoplasia corpus callosum”, “hypoplasia cerebellum”, “hypoplasia brain stem”, and “gray matter heterotopia” recorded in the literature search with mutations in the beginning tail, dimerization, linker, and motor domains. The size of patient groups denoted with the size of circles on the right (smallest circle 10, biggest circle 50). The calculated normalization quotient from Pearson’s chi-square test as described in methods revealed clustering of intellectual disability and behavioral abnormalities in patients with mutations in the beginning tail, linker, and motor domains. Seizures and all MRI abnormalities specifically clustered in patients with mutations in the motor domain. Patients with mutations in the dimerization domain were largely spared of these symptoms, thus underlining the hypothesis that *DYNC1H1*–NMD and –NDD can be traced to specific domain mutations

As described in the methods, we performed a review of the literature for prevalent symptoms in *DYNC1H1*-associated disorders and portrayed the results in a balloon plot using R (Fig. 4b, c). The genotype–phenotype analyses on our patients and the patients found in the literature revealed a significant difference between the different domains of *DYNC1H1*. The statistical analyses revealed that specific mutations in the dimerization domain of *DYNC1H1* corresponded to a NMD phenotype in reported patients with reductions in lower limb strength and mostly preserved upper limb strength (*DYNC1H1*–NMD, Fig. 4b). For the detailed results of the statistical tests, please see Supplementary Table 3.

For patients with specific MRI findings and symptoms associated with NDD, e.g., ID, behavioral abnormalities, and seizures, we perceived that the patients reported in the literature were largely spared from mutations in the dimerization domain (Fig. 4c). Instead, seizures were mostly reported in the motor domain, and ID and behavioral abnormalities were largely reported in the beginning tail, linker, and motor domains. MRI abnormalities were largely reported in the motor domain, specifically pachygyria.

### Genomic analysis and profiling

We identified nine heterozygous novel pathogenic or likely pathogenic variants in *DYNC1H1*; P9’s variant was reported previously in ClinVar (https://www.ncbi.nlm.nih.gov/clinvar/) (Table 1, Fig. 1a–e). All variants described were missing in population databases, classified as likely pathogenic according to the ACMG criteria and were evaluated based on gene constraint statistics (Fig. 1, Table 2). Three mutations were in the tail domains (P1, P3, and P10), one mutation was in the linker domains (P6), four mutations were in the motor domains (P4, P5, P7, and P9), and two mutations in the stalk or MTBD (P2 and P8) (Fig. 1b). Two mutations were located inside (P1, P2) and three mutations close to the coiled coil (CC) domains (P3, P6, and P8), four mutations in the AAA domains (AAA2: P4; AAA3: P7, P9; AAA4: P5) and one in a DYNC1LI2 interaction domain (P10).

**Table 2:** Bioinformatic analyses and pathogenicity scores for *DYNC1H1* variants reported in our ten patients

Patient	Chr:Region (build GRCh38/hg38)	Exon	RefSeq NM_001376.4	GERP++ RS	SIFT	PolyPhen	REVEL	MutationTaster	Mutpred2	CADD-Phred	ClinVar	Population databases	ACMG criteria/ACMG Classification	Justification for pathogenicity
P1	14:101979774–101979774	4/78	Het. c.574G > A, p.Gly192Arg	4.89	0.03	0.83	0.448	1	0.471	28.7	Not reported	Not reported	PM1, PM2, PP2, PP3/Likely pathogenic	Distant CC1 domain, MTR below 10th percentile
P2	14:102033994–102033994	55/78	De novo het. c.10432C > T, p.Leu3478Phe	5.72	0.00	1.00	0.603	1	0.431	34	Not reported	Not reported	PM1, PM2, PM6, PP2, PP3/Likely pathogenic	Mutation in CC7 domain (dynactin–dynein–BICD2-binding) and MTBD, MTR below 10th percentile
P3	14:102000331–102000331	18/78	De novo het. c.4006C > T, p.Leu1336Phe	5.86	0.00	0.996	0.437	1	0.547	33	Not reported	Not reported	PM2, PM6, PP2, PP3/Likely pathogenic	Mutation in linker domain (powerstroke)
P4	14:102012336–102012336	34/78	De novo het. c.6880G > A, p.Glu2294Lys	5.57	0.00	0.999	0.733	1	0.614	34	Not reported	Not reported	PS2, PM2, PP2, PP3/Likely pathogenic	Mutation in motor domain (AAA2)
P5	14:102027537–102027537	46/78	De novo het. c.9041A > G, p.Asn3014Ser	5.67	0.11	0.422	0.278	1	0.264	23.7	Not reported	Not reported	PS2, PM1, PM2, PP2, PP3/Pathogenic	Mutation in motor domain (AAA4), MTR below 25th percentile
P6	14:102002603–102002603	22/78	De novo het. c.4609T > C, p.Trp1537Arg	5.7	0.00	1.0	0.825	1	0.872	27.6	Not reported	Not reported	PM2, PM6, PP2, PP3/Likely pathogenic	Mutation in linker domain (powerstroke)
P7	14:102018507–102018507	41/78	De novo het. c.8234C > A, p.Thr2745Lys	5.26	1.00	0.003	0.332	1	0.523	21	Not reported	Not reported	PM1, PM2, PM6, PP2, PP3/Likely pathogenic	Mutation in motor domain (AAA3)
P8	14:102029588–102029588	49/78	De novo het. c.9518C > G, p.Pro3173Arg	5.37	0.00	1.00	0.694	1	0.628	29.6	Not reported	Not reported	PM2, PM6, PP2, PP3/Likely pathogenic	Microtubule-binding domain (MTBD), MTR on 25th percentile
P9	14:102016944–102016944	38/78	De novo het. c.7793G > T, p.Gly2598Val	5.16	0.00	1.0	0.945	1	0.853	28.8	245840 rs879253 971^a^	Not reported	PM1, PM2, PM6, PP2, PP3, PP5/Pathogenic	Mutation in ATP-binding region of AAA3, MTR below 10th percentile
P10	14:101986223–101986223	8/78	De novo het. c.1998A > T, p.Glu666Asp	−1.11	0.03	0.427	0.314	0.999	0.493	22.8	Not reported	Not reported	PM1, PM2, PP2, PP3/Likely pathogenic	Mutation in domain known to interact with DYNC1LI2; also in tail domain, likely leads to deficient motor ensemble for cortical dynein

*GERP++ RS* Genomic Evolutionary Rate Profiling rejected substitutions score, range from minimum −12.3 to maximum 6.17 [23], *SIFT* Sorting Intolerant from Tolerant Substitutions score, range from 0.00 (deleterious) to 1.00 (tolerated) [34], *PolyPhen* Polymorphism Phenotyping-2 score, range from minimum 0.00 (benign) to maximum 1.00 (damaging) [35], *Revel* REVEL score for predicting the pathogenicity of rare missense variants, range from minimum 0.00 to maximum 1.00 [24], *ClinVar* reports in ClinVar database, *MutationTaster* MutationTaster score, range from minimum 0.00 to maximum 1.00 (http://www.mutationtaster.org/), *MutPred2* MutPred2 score, range from minimum 0.00 to maximum 1.00 (http://mutpred.mutdb.org/), *CADD-Phred* Combined Annotation-Dependent Depletion scores [33], *Population database* reports in Exome Aggregation Consortium (ExAC) database (http://exac.broadinstitute.org), reports in 1000 Genomes Project database (http://www.internationalgenome.org/); reports in genome aggregation database (https://gnomad.broadinstitute.org/), reports in Greater Middle Eastern Database (http://igm.ucsd.edu/gme/data-browser.php), *ACMG criteria* variant classification according to guidelines from the American College of Medical Genetics and Genomics—Association for Molecular Pathology [19], *Justification for pathogenicity* based on reports of mutation pathogenicity in well-described domains [2, 8] (CC: coiled coil domains; AAA: ATPase associated with various cellular activities domains; Fig. 1b), Missense Tolerance Ratio (MTR given in percentiles, demonstrated in Fig. 1c, http://mtr-viewer.mdhs.unimelb.edu.au/)

^a^1 case reported as likely pathogenic without clinical description

We performed additional statistical analyses for variant pathogenicity with multiple prediction tools as described in the methods, which projected the variants in our patient collective to have a highly damaging impact (Fig. 1c–e, Table 2, Supplementary Table 2).

In the comparison between subjects with a two-factor ANOVA, we observed significant differences in CADD-Phred values between the reports “healthy subjects” (mean: 23.809; standard error of mean: 0.25) and “patients” (mean: 29.05; standard error of mean: 0.612) (*p* < 0.001). When differentiating the patient groups in a post hoc test (Bonferroni), there were significant differences between the groups “gnomAD variants” and “ClinVar variants (likely pathogenic and pathogenic)” (two-factor ANOVA, *p* < 0.01), and “gnomAD variants” and “patients’ variants” from our collective (two-factor ANOVA, *p* < 0.05, Fig. 1d, e).

In-depth interaction analyses for CADD-Phred score values uncovered a significant interaction between the group “reports” (patients vs healthy) and “regions” (above-mentioned protein domains) (two-factor ANOVA, *p* < 0.05). Specifically, in the linker domain, the mutations from patients showed significantly higher mean CADD-Phred score values than the variants in healthy subjects (scores in healthy group 23.1 vs patients 32.3, two-factor ANOVA, *p* < 0.001), while other domains showed less drastic differences in the CADD-Phred values between healthy variants and patient mutations (tail 23.6 vs 27.9, motor 24.8 vs 28.5, MTBD 23.7 vs 27.6, two-factor ANOVA, all *p* < 0.001).

In the next step, we evaluated the intolerance toward missense mutations throughout the regions of *DYNC1H1*. The MTR for our patients’ variants showed that these variants were in regions of high intolerance toward missense variations (Fig. 1c). In the comparison between subjects with a two-factor ANOVA, we tested for differences in MTR score values between healthy subjects and patients and observed a significantly higher MTR score values of 0.63 for variants in the healthy population (0.63) than in the patients mutations (0.46), i.e., patient mutations were at amino acid residues with higher intolerance toward missense mutations (*p* < 0.001).

In-depth interaction analyses for MTR score values also revealed significant differences in MTR scores between protein regions (two-factor ANOVA, *p* < 0.001), while mean MTR scores decreased over protein length (tail 0.63 > linker 0.55 > motor 0.55 > MTBD 0.48). In a post hoc test (Bonferroni), we tested for the interactions between reports (healthy vs patients) and protein regions and observed that variants in the linker domain had the highest mean MTR scores in the healthy population (0.72), but the lowest mean MTR scores in patients (0.39) (two-factor ANOVA, *p* < 0.001). Mean MTR scores for other domains also showed dissociations between healthy subjects and patients (tail 0.68 vs 0.57, motor 0.65 vs 0.45, two-factor ANOVA, all *p* < 0.001), while the calculation for MTBD showed similar values between the groups (0.46 vs 0.44, two-factor ANOVA, *p* < 0.001).

## Discussion

We report ten patients with nine novel mutations in the *DYNC1H1* gene (Table 2). In neurons, *DYNC1H1* as part of the cytoplasmic dynein complex is essential for retrograde transport of cargos in axons and dendrites, thus involved in neuronal development, morphology, and survival [7, 9, 45].

In the literature, an increasing number of phenotype expansions have shown an overlapping phenotype link between motor neuropathies and brain malformations [32, 37, 46, 47]. We propose a novel clinical classification of *DYNC1H1*-related disease entities that follows a holistic approach, focusing on the patients’ individual but complex clinical traits in the center of the classification, rather than the current reductionistic classification (e.g., SMALED or MRD13): *DYNC1H1*-related disorders with an exclusive NMD phenotype, *DYNC1H1*–NMD, and a combined NMD–CNS phenotype, *DYNC1H1*–NDD, on either sides of the spectrum (Fig. 4).

### Genomic profiling of population datasets for the domain-specific impact of genetic variation

All mutations in our ten patients showed high CADD scores, high intolerance toward missense variations (MTR, Fig. 1c) in evolutionary well-conserved domains (Fig. 2), and no reports in population databases (Table 2). When comparing healthy with patient report datasets, we observed that pathogenic mutations with significantly higher CADD scores in patients (Table 2) in all domains due to evolutionary conservation and drastic effects on physicochemical properties cause by amino acid exchanges at the mutational sites (*p* < 0.001). This is also evident from multiple lines of in silico pathogenicity scores (Supplementary Table 2). Next, we looked at domain-specific statistical pathogenicity prediction tools. The significant interaction between reports and protein regions revealed particularly higher CADD-Phred score value increases in the linker domain (healthy 23.1 vs patients 32.2, *p* < 0.001), which hints at an evolutionary well-conserved domain (Fig. 2).

The tail domain itself shows rather low conservation throughout species and variants in the tail domains can be observed quite often in human, thus CADD-Phred score values in the healthy dataset are rather low. The tail domain mutations in patients tend to have rather high CADD-Phred score values and are situated at residues with rather high tolerance toward missense variants. The CC regions in the tail domains are highly conserved are also connoted with high CADD-Phred score values in patient mutations. As the CADD scores also comprise protein impact tools (e.g., Grantham, SIFT, and PolyPhen), the amino acid exchanges in the patients mutations show higher CADD scores and more drastic effects in physicochemical properties than in the healthy dataset. Clustered around the CC regions, the MTR scores show significantly higher intolerance toward missense mutations (*p* < 0.001).

The CADD and MTR score values further support that the motor domain is highly conserved and the mutational effects lead to drastic changes on the protein level. MTBD domain variants in the healthy and patient datasets show rather similar MTR scores, thus a similarly high intolerance toward missense variants. When comparing Fig. 1c, d, we note that there is a much lower number of variants as well as significantly lower mean CADD score value in the MTBD (*p* < 0.001), i.e., MTBD variants are generally scarce in healthy population datasets and the protein level scores do not show drastic effects in physiochemical properties.

Based on this genomic profiling of healthy and patient variant datasets, we underline the domain-specific effects of genetic variation in *DYNC1H1* and we further recommend to interpret *DYNC1H1* variants based on the following model:

1. Region location/ conservation: mutations in the linker, motor, and MTBD region are in highly evolutionarily conserved domains with important functional roles in processive and powerstroke movements.
2. Gene-wide missense intolerance: as a measure of regional intolerance to missense variation, the spatial distribution of observed vs expected variants have to be evaluated concerning the healthy population. Patient mutations are situated at genomic coordinates with significantly lower MTR scores, i.e., are more prone to intolerance from missense variation.
3. Protein level change in physicochemical property: based on multiple lines of pathogenicity prediction scoring tools, the effect of the amino acid exchange in a mutation can be evaluated for missense variations for protein structural and functional properties, including secondary structure, solvent accessibility, functional domains, methylation, phosphorylation, and glycosylation.

#### *DYNC1H1*–NMD

In our cohort, no patient was characterized by exclusive *DYNC1H1*–NMD. In the literature, about half of patients reported an exclusive NMD phenotype, predominantly involving the lower limbs (SMALED, CMT20) [1, 30] and presenting with delay in motor milestones, muscle weakness, atrophy hyporeflexia, and skeletal limb abnormalities. Most affected individuals developed secondary orthopedic symptoms like hyperlordosis and feet deformities [32]. However, patients did not exhibit CNS involvement, e.g., ID or cortical malformations (Fig. 4). *DYNC1H1* mutations in patients with NMD were located in the tail domain of *DYNC1H1* (53AA–1867AA), predominantly within the dimerization domain (300AA–1140AA) (Fig. 1a–d). Previous studies demonstrated that these tail domain mutations show no disruption in the retrograde movement of dynein along microtubules, in contrast to motor domain mutations. They rather exclusively shortened the run length of processive dynein–dynactin–BICD2N complexes, leading to a possible disruption of neuronal cargo delivery [8]. A hypothesis why the muscular atrophy in *DYNC1H1*–NMD affects predominantly the lower extremities is that neuronal transportation distance is longer compared with the upper extremity or the cortex, thus being more sensitive by a deduction in run length [8]. Further studies need to evaluate if extrinsic (environmental) or intrinsic factors (methylation, genome interactions) contribute to phenotypic variability.

#### *DYNC1H1*–NDD

A second group of patients presented *DYNC1H1*–NMD with concomitant CNS involvement. In our cohort, all patients presented with predominant lower extremity muscle atrophy, a variable degree of ID, global developmental delay, and/or brain malformations in MRI.

From a recent molecular biological study, mutations in the linker region were observed with deficient powerstroke movements [2]. CC mutations (P1) displayed altered foci for plus-end dynein (dynactin independent) and cortical dynein (dynactin dependent). Furthermore, these mutations resulted in processive movement reductions of the dynein complex [8]. Mutations in MTBD are known to be associated with a reduction in velocity, displacement, and neck transit success, all of which are essential for the stabilization and advancement of movements [8] leading to a more severe disruption of motor activity like in the other variants associated with *DYNC1H1–*NDD. The fact that MTBD domain mutations do not appear in healthy datasets hints at the possibility of early onset lethal disease courses for MTBD domain mutations. Motor domain mutations, with the six AAA domains forming the core complex, were associated with microtubule gliding defects (AAA5) or inhibition of any movement (AAA1), whereas other protein areas (tail, linker, or MTBD) do not alter glinding [8]. Moreover, mutations lead to static binding to microtubule (AAA1) or diffuse binding behavior (AAA5 and MTBD), both resulting in disturbed motor activity and possibly secondarily in severe disruption of neuronal migration and myelination [8, 45]. We identified four AAA domain mutations with NMD, cognitive-behavioral impairment, and brain malformation (P4, P5, P7, P9, Fig. 1a–d**)**. These findings highlight that motor domain mutations lead to MCD due to a more severe disruption of the dynein movement.

P2 displayed cerebral hypomyelination on brain MRI with a mutation in CC7, which is involved in the sedimentation of microtubules with the MTBD in the context of a fusion protein with a heterologous CC [8].

Patients with *DYNC1H1–*NDD had different degrees of ID, learning, and language impairment, in line with our findings. Some reports described more severely affected individuals with epilepsy or spastic paraplegia and variable MCD including pachygyria and polymicrogyria, frequently in combination with ventricular anomalies, abnormal white matter and corpus callosum, and cerebellar hypoplasia [5, 37]. Almost all of our patients had an overlapping phenotype with PNS and CNS involvement, signifying a clinical continuum (Fig. 4). Of note, all patients exhibited rare signs and symptoms closely linked to neuronal development (e.g., cataracts, syrinx, etc.), even patients with tail domain mutations (Table 1).

We propose a novel clinical classification of *DYNC1H1*-related disease entities that follows a holistic approach, focusing on the patients’ individual but complex clinical traits in the center of the classification, rather than the current reductionistic classification (e.g., SMALED or MRD13). Our new classification of *DYNC1H1*-related disorders involves the leading phenotype characteristics, i.e., *DYNC1H1*–NMD as a NMD phenotype and *DYNC1H1*–NDD with concomitant CNS involvement.

## Supplementary information

Supplementary Table 1

Supplementary Table 2

Supplementary Table 3

## Data Availability

Any data not published within the article is available as anonymized data and will be shared by request from the authors. The next-generation sequencing raw data including either gene panel or WES cannot be fully shared publicly since this may facilitate the de-anonymization of the study subjects and also infringe their privacy. We also do not have ethical permission to share the full and raw next-generation sequencing data or full variant list from it individually.
