# Supplementary material for: The clinical-phenotype continuum in DYNC1H1-related disorders—genomic profiling and proposal for a novel classification
Source: J Hum Genet. 2020 Aug 12;65(11):1003–17. doi: 10.1038/s10038-020-0803-1 (PMC7719554; doi:10.1038/s10038-020-0803-1)
Supplement: Supplementary file 1 — Supplementary Table 1 [file 10038_2020_803_MOESM1_ESM.docx]

**SUPPLEMENTARY MATERIAL**

**The clinical phenotype continuum in *DYNC1H1-*related disorders - genomic profiling and proposal for a novel classification**

**Supplementary Table 1**

| P | ref. | pos. mut. | domain | UL strength | LL  strength | ID | behavioural  abnorm. | seizures | MRI abnorm. | pachygyria | heterotopia | enlarged  ventricles | hypoplasia  CC | hypoplasia  brain stem | hypoplasia cerebellum |
| --- | --- | --- | --- | --- | --- | --- | --- | --- | --- | --- | --- | --- | --- | --- | --- |
| 1 | 1 | 598 | 1 |  | 1 |  |  |  | 0 | 0 | 0 | 0 | 0 | 0 | 0 |
| 2 | 1 | 598 | 1 |  | 1 |  | 0 |  | 0 | 0 | 0 | 0 | 0 | 0 | 0 |
| 3 | 1 | 598 | 1 |  | 1 |  |  |  | 0 | 0 | 0 | 0 | 0 | 0 | 0 |
| 4 | 1 | 598 | 1 |  | 1 |  |  |  | 0 | 0 | 0 | 0 | 0 | 0 | 0 |
| 5 | 1 | 598 | 1 |  | 1 |  |  |  | 0 | 0 | 0 | 0 | 0 | 0 | 0 |
| 6 | 1 | 598 | 1 |  | 1 |  | 1 |  | 0 | 0 | 0 | 0 | 0 | 0 | 0 |
| 7 | 1 | 598 | 1 |  | 1 |  |  |  | 0 | 0 | 0 | 0 | 0 | 0 | 0 |
| 8 | 1 | 598 | 1 |  | 1 |  |  |  | 0 | 0 | 0 | 0 | 0 | 0 | 0 |
| 9 | 1 | 598 | 1 |  | 1 |  |  |  | 0 | 0 | 0 | 0 | 0 | 0 | 0 |
| 10 | 1 | 776 | 1 | 0 | 1 | 0 | 0 | 0 | 0 | 0 | 0 | 0 | 0 | 0 | 0 |
| 11 | 1 | 598 | 1 | 1 | 1 | 0 | 0 |  | 0 | 0 | 0 | 0 | 0 | 0 | 0 |
| 12 | 1 | 598 | 1 | 0 | 1 |  |  |  | 0 | 0 | 0 | 0 | 0 | 0 | 0 |
| 13 | 2 | 807 | 1 | 0 | 1 | 0 | 0 | 0 | 0 | 0 | 0 | 0 | 0 | 0 | 0 |
| 14 | 3 | 776 | 1 | 0 | 1 | 0 | 0 | 0 | 0 | 0 | 0 | 0 | 0 | 0 | 0 |
| 15 | 3 | 776 | 1 | 0 | 1 | 0 | 0 | 0 | 0 | 0 | 0 | 0 | 0 | 0 | 0 |
| 16 | 4 | 1518 | 2 |  | 1 | 1 |  | 0 | 1 | 1 | 0 | 0 | 0 | 0 | 0 |
| 17 | 4 | 3822 | 4 |  | 1 | 1 |  | 1 | 1 | 1 | 0 | 1 | 0 | 0 | 0 |
| 18 | 5 | 3360 | 4 | 0 | 1 | 0 |  | 0 |  |  |  |  |  |  |  |
| 19 | 5 | 3360 | 4 | 0 | 1 | 0 |  | 0 |  |  |  |  |  |  |  |
| 20 | 5 | 3360 | 4 | 0 | 1 | 0 |  | 0 |  |  |  |  |  |  |  |
| 21 | 5 | 598 | 1 | 0 | 1 |  |  |  |  |  |  |  |  |  |  |
| 22 | 5 | 1062 | 1 |  | 1 | 1 | 1 | 1 | 1 | 1 | 0 | 0 | 1 | 0 | 1 |
| 23 | 6 | 3392 | 4 |  |  | 1 |  | 1 |  |  |  |  |  |  |  |
| 24 | 7 | 251 | 0 | 1 | 1 | 1 | 1 | 0 | 1 | 0 | 0 | 1 | 0 | 0 | 0 |
| 25 | 7 | 251 | 0 | 1 | 1 | 1 | 1 | 0 | 1 | 0 | 0 | 1 | 0 | 0 | 0 |
| 26 | 7 | 251 | 0 | 1 | 1 | 1 | 1 | 0 | 1 | 0 | 0 | 1 | 0 | 0 | 0 |
| 27 | 7 | 251 | 0 | 1 | 1 | 1 | 1 | 0 | 0 | 0 | 0 | 0 | 0 | 0 | 0 |
| 28 | 8 | 2332 | 4 |  |  | 1 | 1 | 0 | 1 | 1 | 0 | 0 | 0 | 0 | 0 |
| 29 | 9 | 1132 | 1 | 1 | 1 | 1 |  | 0 | 1 | 0 | 0 | 1 | 0 | 0 | 0 |
| 30 | 9 | 3384 | 4 | 1 | 1 | 1 |  | 1 | 1 | 1 | 0 | 0 | 0 | 0 | 0 |
| 31 | 10 | 2720 | 4 |  |  |  |  |  | 1 | 1 | 1 | 0 | 1 | 1 | 1 |
| 32 | 10 | 3951 | 4 |  |  |  |  |  | 1 | 1 | 1 | 0 | 0 | 0 | 1 |
| 33 | 12 | 3658 | 4 | 1 | 1 | 1 |  | 1 | 1 | 1 | 1 | 1 | 1 | 0 | 0 |
| 34 | 13 | 598 | 1 |  | 1 |  |  |  |  |  |  |  |  |  |  |
| 35 | 13 | 598 | 1 |  | 1 |  |  |  |  |  |  |  |  |  |  |
| 36 | 13 | 598 | 1 | 0 | 1 | 0 | 0 | 0 | 1 | 0 | 0 | 1 | 0 | 0 | 0 |
| 37 | 14 | 306 | 1 | 0 | 1 | 0 | 0 | 0 | 0 | 0 | 0 | 0 | 0 | 0 | 0 |
| 38 | 14 | 306 | 1 | 0 | 1 | 0 | 0 | 0 | 0 | 0 | 0 | 0 | 0 | 0 | 0 |
| 39 | 14 | 306 | 1 | 0 | 1 | 0 | 0 | 0 |  |  |  |  |  |  |  |
| 40 | 15 | 603 | 1 | 0 | 1 |  |  |  |  |  |  |  |  |  |  |
| 41 | 15 | 603 | 1 | 0 | 1 |  |  |  |  |  |  |  |  |  |  |
| 42 | 15 | 603 | 1 | 0 | 1 |  |  |  |  |  |  |  |  |  |  |
| 43 | 16 | 598 | 1 | 0 | 1 | 0 | 0 | 0 | 0 | 0 | 0 | 0 | 0 | 0 | 0 |
| 44 | 17 | 584 | 1 | 0 | 1 | 0 |  | 0 |  |  |  |  |  |  |  |
| 45 | 17 | 584 | 1 | 0 | 1 | 0 |  | 0 |  |  |  |  |  |  |  |
| 46 | 17 | 584 | 1 | 0 | 1 | 0 |  | 0 |  |  |  |  |  |  |  |
| 47 | 17 | 584 | 1 | 0 | 1 | 0 |  | 0 |  |  |  |  |  |  |  |
| 48 | 17 | 584 | 1 | 0 | 1 | 0 |  | 0 |  |  |  |  |  |  |  |
| 49 | 17 | 584 | 1 | 0 | 1 | 0 |  | 0 |  |  |  |  |  |  |  |
| 50 | 17 | 584 | 1 | 0 | 1 | 0 |  | 0 |  |  |  |  |  |  |  |
| 51 | 17 | 584 | 1 | 0 | 1 | 0 |  | 0 |  |  |  |  |  |  |  |
| 52 | 17 | 584 | 1 | 0 | 1 | 0 |  | 0 |  |  |  |  |  |  |  |
| 53 | 17 | 584 | 1 | 0 | 1 | 0 |  | 0 |  |  |  |  |  |  |  |
| 54 | 17 | 671 | 1 | 0 | 1 | 0 |  | 0 |  |  |  |  |  |  |  |
| 55 | 17 | 671 | 1 | 0 | 1 | 0 |  | 0 |  |  |  |  |  |  |  |
| 56 | 17 | 671 | 1 | 0 | 1 | 0 |  | 0 |  |  |  |  |  |  |  |
| 57 | 17 | 970 | 1 | 0 | 1 | 1 |  | 0 |  |  |  |  |  |  |  |
| 58 | 18 | 659 | 1 | 1 | 1 | 1 |  | 1 | 1 | 1 | 0 | 0 | 1 | 0 | 0 |
| 59 | 18 | 129 | 0 | 0 | 0 | 1 |  | 1 | 1 | 1 | 0 | 0 | 0 | 0 | 0 |
| 60 | 18 | 3336 | 4 | 1 | 1 | 1 |  | 1 | 1 | 1 | 1 | 0 | 1 | 1 | 1 |
| 61 | 18 | 3384 | 4 | 1 | 1 | 1 |  | 1 | 1 | 1 | 0 | 0 | 1 | 1 | 1 |
| 62 | 18 | 1567 | 2 |  |  | 1 |  | 0 | 1 | 1 | 0 | 0 | 0 | 0 | 0 |
| 63 | 18 | 3344 | 4 |  |  | 1 | 1 | 1 | 1 | 1 | 1 | 0 | 1 | 0 | 0 |
| 64 | 18 | 1962 | 4 |  |  | 1 |  | 1 | 1 | 1 | 0 | 0 | 0 | 0 | 0 |
| 65 | 18 | 3241 | 4 | 0 | 0 | 0 |  | 1 | 1 | 1 | 0 | 0 | 0 | 0 | 0 |
| 66 | 18 | 3241 | 4 |  |  | 1 |  | 1 | 1 | 1 | 0 | 0 | 0 | 0 | 0 |
| 67 | 18 | 3241 | 4 | 0 | 0 | 0 |  | 1 | 1 | 1 | 0 | 0 | 0 | 0 | 0 |
| 68 | 18 | 3344 | 4 |  |  | 1 |  | 1 | 1 | 1 | 0 | 0 | 0 | 0 | 1 |
| 69 | 19 | 580 | 1 |  |  |  |  |  | 1 | 1 | 0 | 0 | 0 | 0 | 0 |
| 70 | 19 | 580 | 1 |  |  |  |  |  | 1 | 1 | 0 | 0 | 0 | 0 | 0 |
| 71 | 19 | 2720 | 4 |  |  |  |  |  | 1 | 1 | 1 | 0 | 1 | 1 | 1 |
| 72 | 19 | 2720 | 4 |  |  |  |  |  | 1 | 1 | 1 | 0 | 1 | 1 | 1 |
| 73 | 19 | 2720 | 4 |  |  |  |  |  | 1 | 1 | 1 | 0 | 1 | 1 | 1 |
| 74 | 20 | 1194 | 2 | 0 | 1 | 1 |  | 0 | 1 | 1 | 0 | 0 | 0 | 0 | 0 |
| 75 | 20 | 3048 | 4 | 1 | 1 | 1 | 1 | 0 | 1 | 1 | 0 | 0 | 0 | 0 | 0 |
| 76 | 21 | 306 | 1 | 1 | 1 |  |  |  |  |  |  |  |  |  |  |
| 77 | 21 | 306 | 1 | 0 | 1 |  | 1 |  |  |  |  |  |  |  |  |
| 78 | 21 | 306 | 1 | 0 | 1 |  |  |  |  |  |  |  |  |  |  |
| 79 | 21 | 306 | 1 | 0 | 1 |  |  |  |  |  |  |  |  |  |  |
| 80 | 21 | 306 | 1 | 1 | 1 |  |  |  |  |  |  |  |  |  |  |
| 81 | 21 | 306 | 1 | 1 | 1 |  |  |  |  |  |  |  |  |  |  |
| 82 | 21 | 306 | 1 | 0 | 1 |  |  |  |  |  |  |  |  |  |  |
| 83 | 21 | 306 | 1 | 0 | 1 |  |  |  |  |  |  |  |  |  |  |
| 84 | 21 | 306 | 1 | 1 | 1 |  |  |  |  |  |  |  |  |  |  |
| 85 | 21 | 306 | 1 | 0 | 1 | 1 | 1 |  |  |  |  |  |  |  |  |
| 86 | 21 | 306 | 1 | 0 | 1 | 1 |  |  |  |  |  |  |  |  |  |
| 87 | 21 | 306 | 1 | 0 | 1 | 1 |  |  |  |  |  |  |  |  |  |
| 88 | 21 | 306 | 1 | 0 | 1 |  |  |  |  |  |  |  |  |  |  |
| 89 | 22 | 399 | 1 | 0 | 1 | 0 |  |  |  |  |  |  |  |  |  |
| 90 | 22 | 399 | 1 | 0 | 1 | 1 | 1 |  |  |  |  |  |  |  |  |
| 91 | 22 | 264 | 0 | 0 | 1 | 1 | 1 |  | 1 | 1 | 0 | 0 | 1 | 0 | 0 |
| 92 | 22 | 970 | 1 | 0 | 1 | 1 | 1 |  | 1 | 1 | 0 | 0 | 0 | 0 | 1 |
| 93 | 22 | 970 | 1 | 0 | 1 | 1 | 1 |  | 1 | 1 | 0 | 0 | 1 | 0 | 0 |
| 94 | 22 | 598 | 1 | 0 | 1 | 0 | 0 |  | 1 | 1 | 0 | 0 | 0 | 0 | 0 |
| 95 | 22 | 598 | 1 | 0 | 1 | 0 | 0 |  |  |  |  |  |  |  |  |
| 96 | 22 | 581 | 1 | 0 | 1 | 0 | 0 |  | 0 | 0 | 0 | 0 | 0 | 0 | 0 |
| 97 | 22 | 673 | 1 | 0 | 1 | 0 | 0 |  |  |  |  |  |  |  |  |
| 98 | 22 | 603 | 1 | 0 | 1 | 0 | 0 |  | 0 | 0 | 0 | 0 | 0 | 0 | 0 |
| 99 | 22 | 1603 | 2 | 0 | 1 | 1 | 1 | 1 | 1 | 1 | 0 | 0 | 0 | 0 | 0 |
| 100 | 22 | 338 | 1 | 1 | 1 | 1 | 1 |  | 0 | 0 | 0 | 0 | 0 | 0 | 0 |
| 101 | 22 | 612 | 1 | 0 | 1 | 0 | 0 |  | 0 | 0 | 0 | 0 | 0 | 0 | 0 |
| 102 | 22 | 612 | 1 | 0 | 1 | 0 | 0 |  |  |  |  |  |  |  |  |
| 103 | 22 | 612 | 1 | 0 | 1 | 0 | 0 |  | 0 | 0 | 0 | 0 | 0 | 0 | 0 |
| 104 | 22 | 612 | 1 | 0 | 0 | 0 | 0 |  | 0 | 0 | 0 | 0 | 0 | 0 | 0 |
| 105 | 22 | 612 | 1 | 0 | 1 | 0 | 1 |  | 0 | 0 | 0 | 0 | 0 | 0 | 0 |
| 106 | 22 | 584 | 1 | 0 | 1 | 1 |  |  | 0 | 0 | 0 | 0 | 0 | 0 | 0 |
| 107 | 22 | 584 | 1 | 0 | 1 | 1 |  | 1 | 1 | 0 | 0 | 1 | 0 | 0 | 0 |
| 108 | 22 | 612 | 1 | 0 | 1 | 1 |  |  | 0 | 0 | 0 | 0 | 0 | 0 | 0 |
| 109 | 22 | 612 | 1 | 0 | 1 | 0 |  |  |  |  |  |  |  |  |  |
| 110 | 22 | 612 | 1 | 0 | 1 | 0 |  |  |  |  |  |  |  |  |  |
| 111 | 22 | 612 | 1 | 0 | 1 | 0 |  |  |  |  |  |  |  |  |  |
| 112 | 22 | 612 | 1 | 0 | 1 | 0 |  |  |  |  |  |  |  |  |  |
| 113 | 22 | 598 | 1 | 0 | 1 | 0 |  |  |  |  |  |  |  |  |  |
| 114 | 22 | 598 | 1 | 0 | 1 | 0 |  |  |  |  |  |  |  |  |  |
| 115 | 22 | 2616 | 4 | 0 | 1 | 1 |  |  |  |  |  |  |  |  |  |
| 116 | 22 | 2616 | 4 | 0 | 1 | 1 |  |  | 1 | 0 | 0 | 1 | 0 | 0 | 0 |
| 117 | 23 | 561 | 1 |  |  |  |  |  | 1 | 1 | 0 | 0 | 1 | 0 | 0 |
| 118 | 23 | 3355 | 4 |  |  |  |  |  | 1 | 1 | 0 | 0 | 1 | 0 | 0 |
| 119 | 23 | 569 | 1 |  |  |  |  |  | 1 | 1 | 0 | 0 | 1 | 0 | 0 |
| 120 | 23 | 309 | 1 |  |  |  |  |  | 1 | 1 | 0 | 0 | 1 | 0 | 0 |
| 121 | 24 | 192 |  | 1 | 1 | 1 | 1 | 0 | 0 | 0 | 0 | 0 | 0 | 0 | 0 |
| 122 | 24 | 3478 |  | 0 | 1 | 1 | 0 | 1 | 1 | 0 | 0 | 1 | 1 | 0 | 0 |
| 123 | 24 | 1336 |  | 0 | 1 | 1 | 0 | 0 | 1 | 0 | 0 | 1 | 0 | 0 | 0 |
| 124 | 24 | 2294 |  | 0 | 1 | 1 | 0 | 1 | 1 | 1 | 0 | 0 | 0 | 0 | 0 |
| 125 | 24 | 3014 |  | 0 | 1 | 1 | 0 | 0 | 1 | 0 | 0 | 0 | 0 | 0 | 0 |
| 126 | 24 | 1537 |  | 1 | 1 | 0 | 0 | 0 | 0 | 0 | 0 | 0 | 0 | 0 | 0 |
| 127 | 24 | 2745 |  | 1 | 1 | 0 | 1 | 0 | 1 | 0 | 0 | 0 | 0 | 0 | 1 |
| 128 | 24 | 3173 |  | 0 | 1 | 1 | 0 | 1 | 1 | 1 | 0 | 1 | 0 | 0 | 0 |
| 129 | 24 | 2598 | 1 | 0 | 0 | 1 | 1 | 0 | 1 | 0 | 0 | 0 | 1 | 0 | 0 |
| 130 | 24 | 666 | 1 | 0 | 0 | 1 | 0 | 1 | 1 | 1 | 1 | 0 | 0 | 1 | 0 |

**Supplementary Table 1: Overview of patients included in phenotype-genotype analyses.** For UL and LL strength “0” is normal and “1” reduced, for ID “0” is not applicated and “1” is applicated, for behavior “0” is normal and “1” is abnormal, for MRI abnormalities “0” is not applicated and “1” is applicated. P, patient; Ref.; Reference: 1^1^, 2^2^, 3^3^, 4^4^, 5^5^, 6^6^, 7^7^, 8^8^, 9^9^, 10^10^, 11^11^, 12^12^, 13^13^, 14^14^, 15^15^, 16^16^, 17^17,18^, 18^19^, 19^20^, 20^21^, 21^22^, 22^23^, 23^24^, 24- from this publication; pos. mut., position of the mutations in the DYNC1H1 protein; Domain: 1-beginning tai domainl , 2-dimerization domain , 3-linker, 4-motor domain; UL, upper limb, LL, lower limb.

**References:**

1. Beecroft SJ, McLean CA, Delatycki MB, et al. Expanding the phenotypic spectrum associated with mutations of DYNC1H1. *Neuromuscul Disord.* 2017;27(7):607-615.

2. Niu Q, Wang X, Shi M, Jin Q. A novel DYNC1H1 mutation causing spinal muscular atrophy with lower extremity predominance. *Neurol Genet.* 2015;1(2):e20.

3. Ding D, Chen Z, Li K, et al. Identification of a de novo DYNC1H1 mutation via WES according to published guidelines. *Sci Rep.* 2016;6:20423.

4. Willemsen MH, Vissers LE, Willemsen MA, et al. Mutations in DYNC1H1 cause severe intellectual disability with neuronal migration defects. *J Med Genet.* 2012;49(3):179-183.

5. Strickland AV, Schabhuttl M, Offenbacher H, et al. Mutation screen reveals novel variants and expands the phenotypes associated with DYNC1H1. *J Neurol.* 2015;262(9):2124-2134.

6. Lin Z, Liu Z, Li X, et al. Whole-exome sequencing identifies a novel de novo mutation in DYNC1H1 in epileptic encephalopathies. *Sci Rep.* 2017;7(1):258.

7. Chan SHS, van Alfen N, Thuestad IJ, et al. A recurrent de novo DYNC1H1 tail domain mutation causes spinal muscular atrophy with lower extremity predominance, learning difficulties and mild brain abnormality. *Neuromuscul Disord.* 2018;28(9):750-756.

8. Gelineau-Morel R, Lukacs M, Weaver KN, Hufnagel RB, Gilbert DL, Stottmann RW. Congenital Cataracts and Gut Dysmotility in a DYNC1H1 Dyneinopathy Patient. *Genes (Basel).* 2016;7(10).

9. Chen Y, Xu Y, Li G, et al. Exome Sequencing Identifies De Novo DYNC1H1 Mutations Associated With Distal Spinal Muscular Atrophy and Malformations of Cortical Development. *J Child Neurol.* 2017;32(4):379-386.

10. Laquerriere A, Maillard C, Cavallin M, et al. Neuropathological Hallmarks of Brain Malformations in Extreme Phenotypes Related to DYNC1H1 Mutations. *J Neuropathol Exp Neurol.* 2017;76(3):195-205.

11. de Ligt J, Willemsen MH, van Bon BW, et al. Diagnostic exome sequencing in persons with severe intellectual disability. *N Engl J Med.* 2012;367(20):1921-1929.

12. Hertecant J, Komara M, Nagi A, Suleiman J, Al-Gazali L, Ali BR. A novel de novo mutation in DYNC1H1 gene underlying malformation of cortical development and cataract. *Meta Gene.* 2016;9:124-127.

13. Peeters K, Bervoets S, Chamova T, et al. Novel mutations in the DYNC1H1 tail domain refine the genetic and clinical spectrum of dyneinopathies. *Hum Mutat.* 2015;36(3):287-291.

14. Tsurusaki Y, Saitoh S, Tomizawa K, et al. A DYNC1H1 mutation causes a dominant spinal muscular atrophy with lower extremity predominance. *Neurogenetics.* 2012;13(4):327-332.

15. Das J, Lilleker JB, Jabbal K, Ealing J. A missense mutation in DYNC1H1 gene causing spinal muscular atrophy - Lower extremity, dominant. *Neurol Neurochir Pol.* 2018;52(2):293-297.

16. Punetha J, Monges S, Franchi ME, Hoffman EP, Cirak S, Tesi-Rocha C. Exome Sequencing Identifies DYNC1H1 Variant Associated With Vertebral Abnormality and Spinal Muscular Atrophy With Lower Extremity Predominance. *Pediatr Neurol.* 2015;52(2):239-244.

17. Harms MB, Allred P, Gardner R, Jr., et al. Dominant spinal muscular atrophy with lower extremity predominance: linkage to 14q32. *Neurology.* 2010;75(6):539-546.

18. Harms MB, Ori-McKenney KM, Scoto M, et al. Mutations in the tail domain of DYNC1H1 cause dominant spinal muscular atrophy. *Neurology.* 2012;78(22):1714-1720.

19. Poirier K, Lebrun N, Broix L, et al. Mutations in TUBG1, DYNC1H1, KIF5C and KIF2A cause malformations of cortical development and microcephaly. *Nat Genet.* 2013;45(6):639-647.

20. Zillhardt JL, Poirier K, Broix L, et al. Mosaic parental germline mutations causing recurrent forms of malformations of cortical development. *Eur J Hum Genet.* 2016;24(4):611-614.

21. Fiorillo C, Moro F, Yi J, et al. Novel dynein DYNC1H1 neck and motor domain mutations link distal spinal muscular atrophy and abnormal cortical development. *Hum Mutat.* 2014;35(3):298-302.

22. Weedon MN, Hastings R, Caswell R, et al. Exome sequencing identifies a DYNC1H1 mutation in a large pedigree with dominant axonal Charcot-Marie-Tooth disease. *Am J Hum Genet.* 2011;89(2):308-312.

23. Scoto M, Rossor AM, Harms MB, et al. Novel mutations expand the clinical spectrum of DYNC1H1-associated spinal muscular atrophy. *Neurology.* 2015;84(7):668-679.

24. Jamuar SS, Lam AT, Kircher M, et al. Somatic mutations in cerebral cortical malformations. *N Engl J Med.* 2014;371(8):733-743.
