# Supplementary material for: The clinical-phenotype continuum in DYNC1H1-related disorders—genomic profiling and proposal for a novel classification
Source: J Hum Genet. 2020 Aug 12;65(11):1003–17. doi: 10.1038/s10038-020-0803-1 (PMC7719554; doi:10.1038/s10038-020-0803-1)
Supplement: Supplementary file 2 — Supplementary Table 2 [file 10038_2020_803_MOESM2_ESM.docx]

**SUPPLEMENTARY MATERIAL**

**The clinical phenotype continuum in *DYNC1H1-*related disorders - genomic profiling and proposal for a novel classification**

**Supplementary Table 2**

| Chr. | Pos.  (GRCh38) | Ref | Alt | Variation  type | gnomAD AF | Exon | AA  residue | AA  exchange | report | region | CADD-Phred | GERP  RS | Mut  Pred | Mutation  Taster | REVEL | SIFT | Poly  phen | MTR  Obs. | MTR exp. | MTR score | MTR  centile |
| --- | --- | --- | --- | --- | --- | --- | --- | --- | --- | --- | --- | --- | --- | --- | --- | --- | --- | --- | --- | --- | --- |
| 14 | 101964710 | G | A | missense | 0,000004600 | 01/78 | 7 | G/S | healthy | tail domain | 22,90 | 4,24 | 0,15 | 1,00 | 0,21 | 0,50 | 0,06 | 0,44 | 0,72 | 0,62 | 56,12 |
| 14 | 101964710 | G | C | missense | 0,000004600 | 01/78 | 7 | G/R | healthy | tail domain | 23,50 | 4,24 | 0,21 | 1,00 | 0,12 | 0,19 | 0,27 | 0,44 | 0,72 | 0,62 | 56,12 |
| 14 | 101964713 | G | A | missense | 0,000045600 | 01/78 | 8 | G/S | healthy | tail domain | 24,30 | 4,24 | 0,15 | 1,00 | 0,19 | 0,97 | 0,99 | 0,44 | 0,72 | 0,62 | 56,49 |
| 14 | 101964774 | C | T | missense | 0,000082000 | 01/78 | 28 | S/L | healthy | tail domain | 23,30 | 4,23 | 0,29 | 1,00 | 0,06 | 0,11 | 0,00 | 0,27 | 0,71 | 0,38 | 17,39 |
| 14 | 101964783 | A | G | missense | 0,000004480 | 01/78 | 31 | Q/R | healthy | tail domain | 23,50 | 4,23 | 0,30 | 0,99 | 0,07 | 0,52 | 0,02 | 0,39 | 0,71 | 0,54 | 41,94 |
| 14 | 101964825 | G | C | missense | 0,000004570 | 01/78 | 45 | G/A | healthy | tail domain | 23,30 | 4,23 | 0,23 | 1,00 | 0,07 | 0,22 | 0,00 | 0,59 | 0,72 | 0,81 | 89,54 |
| 14 | 101964824 | G | C | missense | 0,000004570 | 01/78 | 45 | G/R | healthy | tail domain | 24,00 | 4,23 | 0,25 | 1,00 | 0,07 | 0,14 | 0,07 | 0,59 | 0,72 | 0,81 | 89,54 |
| 14 | 101964832 | G | C | missense | 0,000004630 | 01/78 | 47 | E/D | healthy | tail domain | 22,40 | 2,15 | 0,19 | 0,86 | 0,04 | 0,40 | 0,00 | NA | NA |  | NA |
| 14 | 101964833 | G | A | missense | 0,000004650 | 01/78 | 48 | A/T | healthy | tail domain | 23,10 | 4,23 | 0,18 | 1,00 | 0,06 | 0,49 | 0,00 | 0,50 | 0,73 | 0,69 | 70,09 |
| 14 | 101964836 | C | T | missense | 0,000004630 | 01/78 | 49 | P/S | healthy | tail domain | 22,50 | 3,33 | 0,33 | 1,00 | 0,07 | 0,47 | 0,05 | 0,50 | 0,73 | 0,69 | 70,09 |
| 14 | 101964836 | C | G | missense | 0,000004630 | 01/78 | 49 | P/A | healthy | tail domain | 22,60 | 3,33 | 0,24 | 1,00 | 0,07 | 0,35 | 0,00 | 0,50 | 0,73 | 0,69 | 70,09 |
| 14 | 101964837 | C | T | missense | 0,000004640 | 01/78 | 49 | P/L | healthy | tail domain | 23,20 | 4,23 | 0,32 | 1,00 | 0,08 | 0,16 | 0,42 | 0,50 | 0,73 | 0,69 | 70,09 |
| 14 | 101964837 | C | G | missense | 0,000004640 | 01/78 | 49 | P/R | healthy | tail domain | 23,20 | 4,23 | 0,38 | 1,00 | 0,09 | 0,18 | 0,15 | 0,50 | 0,73 | 0,69 | 70,09 |
| 14 | 101964850 | G | C | missense | 0,000004660 | 01/78 | 53 | E/D | healthy | tail domain | 22,70 | 4,23 | 0,28 | 1,00 | 0,06 | 0,62 | 0,00 | 0,71 | 0,73 | 0,97 | 97,48 |
| 14 | 101964886 | G | A | missense | 0,000004490 | 01/78 | 65 | M/I | healthy | tail domain | 23,80 | 4,23 | 0,36 | 1,00 | 0,19 | 0,76 | 0,00 | 0,55 | 0,74 | 0,74 | 79,58 |
| 14 | 101964891 | A | G | missense | 0,000004470 | 01/78 | 67 | K/R | healthy | tail domain | 23,50 | 4,23 | 0,26 | 1,00 | 0,06 | 1,00 | 0,03 | 0,50 | 0,75 | 0,67 | 67,29 |
| 14 | 101964904 | C | G | missense | 0,000004490 | 01/78 | 71 | D/E | healthy | tail domain | 19,49 | 2,35 | 0,40 | 1,00 | 0,14 | 0,41 | 0,08 | 0,46 | 0,75 | 0,60 | 53,91 |
| 14 | 101964930 | A | G | missense | 0,000269300 | 01/78 | 80 | E/G | healthy | tail domain | 24,50 | 4,08 | 0,47 | 1,00 | 0,22 | 0,05 | 0,05 | 0,58 | 0,77 | 0,76 | 81,82 |
| 14 | 101975718 | T | C | missense | 0,000003980 | 02/78 | 88 | V/A | healthy | tail domain | 21,20 | 5,44 | 0,24 | 1,00 | 0,17 | 0,77 | 0,01 | 0,36 | 0,78 | 0,47 | 29,01 |
| 14 | 101975720 | G | A | missense | 0,000027900 | 02/78 | 89 | G/S | healthy | tail domain | 22,80 | 5,44 | 0,00 | 1,00 | 0,22 | 0,47 | 0,00 | 0,36 | 0,78 | 0,47 | 29,05 |
| 14 | 101975749 | C | A | missense | 0,000007960 | 02/78 | 98 | F/L | healthy | tail domain | 14,62 | 0,71 | 0,28 | 1,00 | 0,29 | 0,76 | 0,00 | 0,42 | 0,79 | 0,53 | 39,38 |
| 14 | 101975768 | A | G | missense | 0,000003980 | 02/78 | 105 | I/V | healthy | tail domain | 21,40 | 5,44 | 0,49 | 1,00 | 0,13 | 0,37 | 0,00 | 0,40 | 0,80 | 0,50 | 35,08 |
| 14 | 101975790 | A | G | missense | 0,000007960 | 02/78 | 112 | K/R | healthy | tail domain | 23,00 | 5,35 | 0,00 | 1,00 | 0,17 | 0,35 | 0,01 | 0,41 | 0,79 | 0,52 | 38,69 |
| 14 | 101975796 | A | G | missense | 0,000055800 | 02/78 | 114 | N/S | healthy | tail domain | 19,05 | 4,17 | 0,37 | 1,00 | 0,05 | 0,67 | 0,00 | 0,38 | 0,79 | 0,48 | 30,82 |
| 14 | 101979339 | C | T | missense | 0,000007960 | 03/78 | 122 | T/I | healthy | tail domain | 19,33 | 5,68 | 0,46 | 1,00 | 0,14 | 0,19 | 0,14 | 0,53 | 0,76 | 0,70 | 71,57 |
| 14 | 101979344 | G | A | missense | 0,000003980 | 03/78 | 124 | V/M | healthy | tail domain | 19,61 | 5,68 | 0,36 | 1,00 | 0,10 | 0,19 | 0,04 | 0,47 | 0,76 | 0,63 | 57,22 |
| 14 | 101979344 | G | T | missense | 0,000007950 | 03/78 | 124 | V/L | healthy | tail domain | 21,20 | 5,68 | 0,33 | 1,00 | 0,13 | 0,08 | 0,15 | 0,47 | 0,76 | 0,63 | 57,22 |
| 14 | 101979365 | G | A | missense | 0,000003980 | 03/78 | 131 | V/M | healthy | tail domain | 23,00 | 5,68 | 0,35 | 1,00 | 0,17 | 0,03 | 0,20 | 0,45 | 0,75 | 0,60 | 53,30 |
| 14 | 101979381 | G | A | missense | 0,000007950 | 03/78 | 136 | R/Q | healthy | tail domain | 21,20 | 5,68 | 0,38 | 1,00 | 0,17 | 1,00 | 0,01 | 0,43 | 0,75 | 0,57 | 47,30 |
| 14 | 101979380 | C | T | missense | 0,000015900 | 03/78 | 136 | R/W | healthy | tail domain | 25,40 | 5,68 | 0,49 | 1,00 | 0,34 | 0,04 | 0,63 | 0,43 | 0,75 | 0,57 | 47,30 |
| 14 | 101979383 | G | A | missense | 0,000194900 | 03/78 | 137 | V/I | healthy | tail domain | 20,60 | 4,79 | 0,00 | 1,00 | 0,13 | 0,44 | 0,01 | 0,48 | 0,75 | 0,64 | 59,50 |
| 14 | 101979396 | G | A | missense | 0,000047700 | 03/78 | 141 | S/N | healthy | tail domain | 21,90 | 5,36 | 0,00 | 1,00 | 0,11 | 0,26 | 0,01 | 0,53 | 0,75 | 0,70 | 72,82 |
| 14 | 101979411 | A | G | missense | 0,000003980 | 03/78 | 146 | Y/C | healthy | tail domain | 24,90 | 5,36 | 0,46 | 1,00 | 0,54 | 0,00 | 0,78 | 0,59 | 0,76 | 0,78 | 84,57 |
| 14 | 101979429 | T | A | missense | 0,000015900 | 03/78 | 152 | F/Y | healthy | tail domain | 19,61 | 4,21 | 0,52 | 1,00 | 0,16 | 1,00 | 0,00 | 0,60 | 0,76 | 0,79 | 86,36 |
| 14 | 101979431 | A | C | missense | 0,000007950 | 03/78 | 153 | I/L | healthy | tail domain | 18,97 | 5,25 | 0,00 | 1,00 | 0,20 | 1,00 | 0,00 | 0,60 | 0,76 | 0,79 | 86,03 |
| 14 | 101979431 | A | G | missense | 0,000230600 | 03/78 | 153 | I/V | healthy | tail domain | 22,80 | 5,25 | 0,44 | 1,00 | 0,20 | 0,27 | 0,07 | 0,60 | 0,76 | 0,79 | 86,03 |
| 14 | 101979438 | A | G | missense | 0,000003980 | 03/78 | 155 | N/S | healthy | tail domain | 21,30 | 5,36 | 0,39 | 1,00 | 0,09 | 0,40 | 0,03 | 0,69 | 0,76 | 0,91 | 95,55 |
| 14 | 101979467 | A | G | missense | 0,000007950 | 03/78 | 165 | I/V | healthy | tail domain | 17,00 | 4,20 | 0,32 | 1,00 | 0,11 | 1,00 | 0,00 | 0,88 | 0,78 | 1,12 | 99,72 |
| 14 | 101979474 | A | G | missense | 0,000003980 | 03/78 | 167 | E/G | healthy | tail domain | 24,10 | 5,36 | 0,33 | 1,00 | 0,28 | 0,22 | 0,08 | 0,70 | 0,78 | 0,90 | 95,07 |
| 14 | 101979477 | C | G | missense | 0,000003980 | 03/78 | 168 | S/C | healthy | tail domain | 26,80 | 5,36 | 0,00 | 1,00 | 0,20 | 0,01 | 0,70 | 0,75 | 0,78 | 0,96 | 97,31 |
| 14 | 101979764 | A | T | missense | 0,000007960 | 04/78 | 188 | E/D | healthy | tail domain | 18,11 | -2,20 | 0,41 | 1,00 | 0,17 | 0,14 | 0,17 | NA | NA |  | NA |
| 14 | 101979774 | G | A | missense | 0,000000000 | 04/78 | 192 | G/R | study patient | tail domain | 30,00 | 4,89 | 0,47 | 1,00 | 0,45 | 0,03 | 0,83 | NA | NA |  | NA |
| 14 | 101979793 | A | G | missense | 0,000003980 | 04/78 | 198 | Q/R | healthy | tail domain | 27,50 | 4,89 | 0,31 | 1,00 | 0,32 | 0,00 | 0,98 | 0,46 | 0,78 | 0,58 | 50,03 |
| 14 | 101979823 | C | T | missense | 0,000075600 | 04/78 | 208 | P/L | healthy | tail domain | 21,70 | 4,89 | 0,00 | 1,00 | 0,07 | 0,23 | 0,00 | 0,55 | 0,79 | 0,69 | 70,78 |
| 14 | 101979825 | A | G | missense | 0,000011900 | 04/78 | 209 | I/V | healthy | tail domain | 18,39 | 1,11 | 0,40 | 1,00 | 0,09 | 0,30 | 0,02 | 0,60 | 0,78 | 0,77 | 83,22 |
| 14 | 101979834 | A | G | missense | 0,000047700 | 04/78 | 212 | M/V | healthy | tail domain | 17,72 | 2,21 | 0,00 | 1,00 | 0,13 | 1,00 | 0,00 | 0,70 | 0,78 | 0,90 | 94,90 |
| 14 | 101979868 | G | A | missense | 0,000011900 | 04/78 | 223 | R/H | healthy | tail domain | 23,30 | 5,08 | 0,41 | 1,00 | 0,14 | 0,11 | 0,24 | 0,69 | 0,78 | 0,89 | 94,60 |
| 14 | 101979867 | C | T | missense | 0,000111300 | 04/78 | 223 | R/C | healthy | tail domain | 27,00 | 5,08 | 0,43 | 1,00 | 0,19 | 0,01 | 0,54 | 0,69 | 0,78 | 0,89 | 94,60 |
| 14 | 101979879 | C | A | missense | 0,000007950 | 04/78 | 227 | P/T | healthy | tail domain | 24,70 | 5,08 | 0,33 | 1,00 | 0,20 | 0,02 | 0,45 | 0,73 | 0,78 | 0,93 | 96,73 |
| 14 | 101979891 | G | A | missense | 0,000003980 | 04/78 | 231 | D/N | healthy | tail domain | 25,70 | 5,08 | 0,40 | 1,00 | 0,44 | 0,15 | 1,00 | 0,64 | 0,78 | 0,82 | 90,04 |
| 14 | 101979902 | T | G | missense | 0,000059700 | 04/78 | 234 | D/E | healthy | tail domain | 16,43 | -1,57 | 0,23 | 1,00 | 0,17 | 0,38 | 0,02 | 0,64 | 0,77 | 0,82 | 90,34 |
| 14 | 101979906 | G | A | missense | 0,000023900 | 04/78 | 236 | V/I | healthy | tail domain | 23,20 | 5,08 | 0,00 | 1,00 | 0,17 | 0,18 | 0,44 | 0,70 | 0,78 | 0,90 | 95,29 |
| 14 | 101979918 | A | G | missense | 0,000003980 | 04/78 | 240 | T/A | healthy | tail domain | 20,60 | 5,08 | 0,38 | 1,00 | 0,11 | 0,59 | 0,07 | 0,56 | 0,78 | 0,72 | 75,00 |
| 14 | 101980380 | G | A | missense | 0,000000000 | 03/78 | 264 | R/L | patient | tail domain | 34,00 | 5,54 | 0,51 | 1,00 | 0,78 | 0,02 | 0,99 | 0,22 | 0,75 | 0,30 | 9,66 |
| 14 | 101980389 | C | A | missense | 0,000007950 | 05/78 | 267 | A/E | healthy | tail domain | 22,40 | 5,54 | 0,56 | 1,00 | 0,16 | 0,50 | 0,02 | 0,20 | 0,75 | 0,27 | 7,68 |
| 14 | 101980400 | G | A | missense | 0,000007950 | 05/78 | 271 | A/T | healthy | tail domain | 25,80 | 5,54 | 0,54 | 1,00 | 0,32 | 0,07 | 0,67 | 0,25 | 0,76 | 0,33 | 12,85 |
| 14 | 101980418 | T | G | missense | 0,000000000 | 03/78 | 277 | F/V | patient | tail domain | 29,90 | 5,54 | 0,88 | 1,00 | 0,86 | 0,00 | 1,00 | 0,33 | 0,76 | 0,44 | 25,20 |
| 14 | 101980440 | C | T | missense | 0,000007950 | 05/78 | 284 | A/V | healthy | tail domain | 24,80 | 5,24 | 0,65 | 1,00 | 0,38 | 0,03 | 0,99 | 0,33 | 0,75 | 0,45 | 27,16 |
| 14 | 101980448 | C | T | missense | 0,000003980 | 05/78 | 287 | R/C | healthy | tail domain | 26,30 | 5,24 | 0,00 | 1,00 | 0,36 | 0,04 | 0,91 | 0,38 | 0,76 | 0,50 | 34,30 |
| 14 | 101980473 | C | T | missense | 0,000007950 | 05/78 | 295 | P/L | healthy | tail domain | 23,00 | 5,24 | 0,53 | 1,00 | 0,26 | 0,28 | 0,02 | 0,54 | 0,77 | 0,70 | 72,37 |
| 14 | 101980478 | G | A | missense | 0,000003980 | 05/78 | 297 | V/I | healthy | tail domain | 23,40 | 5,24 | 0,74 | 1,00 | 0,20 | 0,18 | 0,13 | 0,58 | 0,77 | 0,76 | 81,97 |
| 14 | 101980494 | A | G | missense | 0,000003980 | 05/78 | 302 | D/G | healthy | tail domain | 24,10 | 5,24 | 0,57 | 1,00 | 0,30 | 0,05 | 0,06 | 0,64 | 0,76 | 0,84 | 91,76 |
| 14 | 101980493 | G | A | missense | 0,000003980 | 05/78 | 302 | D/N | healthy | tail domain | 24,30 | 5,24 | 0,55 | 1,00 | 0,25 | 0,08 | 0,08 | 0,64 | 0,76 | 0,84 | 91,76 |
| 14 | 101980505 | C | A | missense | 0,000003980 | 05/78 | 306 | H/N | healthy | tail domain | 24,10 | 5,24 | 0,46 | 1,00 | 0,27 | 0,07 | 0,66 | 0,50 | 0,76 | 0,66 | 64,73 |
| 14 | 101980515 | G | A | missense | 0,000000000 | 03/78 | 309 | R/H | patient | tail domain | 33,00 | 5,24 | 0,58 | 1,00 | 0,56 | 0,00 | 1,00 | 0,50 | 0,76 | 0,66 | 65,25 |
| 14 | 101980529 | G | A | missense | 0,000011900 | 05/78 | 314 | V/I | healthy | tail domain | 26,60 | 5,24 | 0,53 | 1,00 | 0,23 | 0,04 | 0,75 | 0,56 | 0,76 | 0,73 | 77,51 |
| 14 | 101983030 | G | A | missense | 0,000016000 | 06/78 | 325 | A/T | healthy | tail domain | 24,10 | 5,88 | 0,78 | 1,00 | 0,36 | 0,11 | 0,26 | 0,44 | 0,78 | 0,57 | 47,62 |
| 14 | 101983051 | T | C | missense | 0,000016000 | 06/78 | 332 | Y/H | healthy | tail domain | 28,80 | 5,88 | 0,82 | 1,00 | 0,67 | 0,02 | 1,00 | 0,42 | 0,76 | 0,55 | 42,59 |
| 14 | 101983070 | A | G | missense | 0,000000000 | 06/78 | 338 | D/G, D/V | patient | tail domain | 24,20 | 5,88 | 0,55 | 1,00 | 0,58 | 0,05 | 0,39 | 0,57 | 0,76 | 0,76 | 81,82 |
| 14 | 101983075 | C | T | missense | 0,000003980 | 06/78 | 340 | P/S | healthy | tail domain | 26,00 | 5,88 | 0,62 | 1,00 | 0,56 | 0,05 | 1,00 | 0,53 | 0,75 | 0,71 | 73,66 |
| 14 | 101983079 | T | C | missense | 0,000003980 | 06/78 | 341 | L/P | healthy | tail domain | 29,90 | 5,88 | 0,69 | 1,00 | 0,58 | 0,00 | 0,96 | 0,47 | 0,76 | 0,62 | 55,78 |
| 14 | 101983085 | A | G | missense | 0,000007960 | 06/78 | 343 | D/G | healthy | tail domain | 28,30 | 5,88 | 0,63 | 1,00 | 0,65 | 0,01 | 0,68 | 0,50 | 0,77 | 0,65 | 63,35 |
| 14 | 101983100 | C | T | missense | 0,000007960 | 06/78 | 348 | T/M | healthy | tail domain | 25,60 | 5,88 | 0,00 | 1,00 | 0,43 | 0,02 | 0,89 | 0,43 | 0,76 | 0,57 | 46,37 |
| 14 | 101983104 | G | T | missense | 0,000003980 | 06/78 | 349 | E/D | healthy | tail domain | 15,90 | 3,06 | 0,43 | 1,00 | 0,19 | 1,00 | 0,03 | 0,43 | 0,77 | 0,56 | 45,43 |
| 14 | 101983112 | A | G | missense | 0,000003980 | 06/78 | 352 | K/R | healthy | tail domain | 23,20 | 5,88 | 0,41 | 1,00 | 0,08 | 0,28 | 0,01 | 0,40 | 0,76 | 0,52 | 38,69 |
| 14 | 101983163 | A | G | missense | 0,000007950 | 06/78 | 369 | N/S | healthy | tail domain | 22,20 | 5,88 | 0,43 | 1,00 | 0,11 | 0,51 | 0,04 | 0,42 | 0,76 | 0,55 | 44,07 |
| 14 | 101983177 | A | G | missense | 0,000015900 | 06/78 | 374 | I/V | healthy | tail domain | 22,60 | 5,88 | 0,00 | 1,00 | 0,18 | 0,15 | 0,08 | 0,42 | 0,75 | 0,56 | 44,61 |
| 14 | 101983193 | G | T | missense | 0,000003980 | 06/78 | 379 | R/L | healthy | tail domain | 24,00 | 5,88 | 0,57 | 1,00 | 0,42 | 0,30 | 0,11 | 0,43 | 0,75 | 0,57 | 47,97 |
| 14 | 101983192 | C | T | missense | 0,000015900 | 06/78 | 379 | R/C | healthy | tail domain | 31,00 | 5,88 | 0,59 | 1,00 | 0,61 | 0,04 | 0,94 | 0,43 | 0,75 | 0,57 | 47,97 |
| 14 | 101983207 | A | G | missense | 0,000003980 | 06/78 | 384 | I/V | healthy | tail domain | 23,00 | 5,88 | 0,73 | 1,00 | 0,23 | 0,12 | 0,31 | 0,43 | 0,76 | 0,57 | 46,85 |
| 14 | 101983234 | C | T | missense | 0,000003980 | 06/78 | 393 | L/F | healthy | tail domain | 22,20 | 5,88 | 0,46 | 1,00 | 0,23 | 0,31 | 0,13 | 0,56 | 0,77 | 0,73 | 77,99 |
| 14 | 101983265 | A | G | missense | 0,000003980 | 06/78 | 403 | H/R | healthy | tail domain | 23,00 | 5,88 | 0,48 | 1,00 | 0,39 | 0,07 | 0,15 | 0,50 | 0,79 | 0,63 | 59,09 |
| 14 | 101983274 | A | T | missense | 0,000003980 | 06/78 | 406 | Y/F | healthy | tail domain | 23,10 | 5,88 | 0,61 | 1,00 | 0,15 | 0,41 | 0,02 | 0,54 | 0,81 | 0,67 | 67,29 |
| 14 | 101983273 | T | C | missense | 0,000003980 | 06/78 | 406 | Y/H | healthy | tail domain | 24,00 | 5,88 | 0,00 | 1,00 | 0,26 | 0,11 | 0,04 | 0,54 | 0,81 | 0,67 | 67,29 |
| 14 | 101983274 | A | G | missense | 0,000003980 | 06/78 | 406 | Y/C | healthy | tail domain | 32,00 | 5,88 | 0,69 | 1,00 | 0,55 | 0,00 | 0,94 | 0,54 | 0,81 | 0,67 | 67,29 |
| 14 | 101983280 | A | G | missense | 0,000003980 | 06/78 | 408 | E/G | healthy | tail domain | 31,00 | 5,88 | 0,55 | 1,00 | 0,57 | 0,00 | 0,96 | 0,57 | 0,81 | 0,71 | 73,92 |
| 14 | 101983408 | T | G | missense | 0,000007960 | 07/78 | 420 | F/L | healthy | tail domain | 19,30 | -6,26 | 0,62 | 1,00 | 0,39 | 0,02 | 0,39 | 0,55 | 0,81 | 0,67 | 67,44 |
| 14 | 101983412 | A | G | missense | 0,000003980 | 07/78 | 422 | T/A | healthy | tail domain | 22,20 | 5,58 | 0,50 | 1,00 | 0,19 | 0,33 | 0,02 | 0,38 | 0,82 | 0,46 | 28,51 |
| 14 | 101983460 | G | A | missense | 0,000003980 | 07/78 | 438 | V/I | healthy | tail domain | 23,10 | 5,58 | 0,46 | 1,00 | 0,12 | 0,26 | 0,03 | 0,25 | 0,81 | 0,31 | 11,17 |
| 14 | 101983511 | G | A | missense | 0,000003980 | 07/78 | 455 | A/T | healthy | tail domain | 23,30 | 5,58 | 0,00 | 1,00 | 0,20 | 0,52 | 0,25 | 0,60 | 0,80 | 0,75 | 80,59 |
| 14 | 101983538 | G | A | missense | 0,000019900 | 07/78 | 464 | D/N | healthy | tail domain | 25,40 | 5,58 | 0,46 | 1,00 | 0,14 | 0,28 | 0,50 | 0,50 | 0,77 | 0,65 | 62,77 |
| 14 | 101983542 | A | G | missense | 0,000003980 | 07/78 | 465 | Q/R | healthy | tail domain | 23,20 | 5,58 | 0,44 | 1,00 | 0,15 | 0,21 | 0,08 | 0,44 | 0,77 | 0,58 | 48,57 |
| 14 | 101983560 | G | A | missense | 0,000007960 | 07/78 | 471 | R/H | healthy | tail domain | 24,20 | 5,58 | 0,39 | 1,00 | 0,22 | 0,24 | 0,09 | 0,46 | 0,75 | 0,61 | 54,12 |
| 14 | 101983589 | G | C | missense | 0,000003980 | 07/78 | 481 | V/L | healthy | tail domain | 23,00 | 5,58 | 0,41 | 1,00 | 0,18 | 0,34 | 0,01 | 0,39 | 0,76 | 0,51 | 36,37 |
| 14 | 101983590 | T | A | missense | 0,000039800 | 07/78 | 481 | V/D | healthy | tail domain | 24,20 | 5,58 | 0,48 | 1,00 | 0,40 | 0,09 | 0,17 | 0,39 | 0,76 | 0,51 | 36,37 |
| 14 | 101985691 | C | T | missense | 0,000055700 | 08/78 | 489 | T/M | healthy | tail domain | 22,90 | 5,77 | 0,39 | 1,00 | 0,11 | 0,07 | 0,15 | 0,56 | 0,76 | 0,73 | 78,22 |
| 14 | 101985702 | C | A | missense | 0,000003980 | 08/78 | 493 | Q/K | healthy | tail domain | 20,70 | 5,86 | 0,51 | 1,00 | 0,23 | 0,91 | 0,04 | 0,61 | 0,76 | 0,81 | 88,60 |
| 14 | 101985714 | G | A | missense | 0,000003980 | 08/78 | 497 | G/R | healthy | tail domain | 25,40 | 5,86 | 0,46 | 1,00 | 0,24 | 0,05 | 0,24 | 0,59 | 0,75 | 0,78 | 85,43 |
| 14 | 101985719 | G | C | missense | 0,000011900 | 08/78 | 498 | E/D | healthy | tail domain | 13,14 | -1,16 | 0,00 | 0,99 | 0,06 | 0,59 | 0,00 | 0,59 | 0,76 | 0,78 | 84,92 |
| 14 | 101985721 | T | C | missense | 0,000003980 | 08/78 | 499 | V/A | healthy | tail domain | 11,48 | 3,53 | 0,50 | 0,58 | 0,06 | 0,92 | 0,00 | 0,56 | 0,76 | 0,74 | 78,91 |
| 14 | 101985729 | C | A | missense | 0,000003980 | 08/78 | 502 | P/T | healthy | tail domain | 19,34 | 5,86 | 0,34 | 1,00 | 0,13 | 0,43 | 0,01 | 0,53 | 0,77 | 0,69 | 70,20 |
| 14 | 101985730 | C | T | missense | 0,000003980 | 08/78 | 502 | P/L | healthy | tail domain | 22,30 | 5,86 | 0,41 | 1,00 | 0,22 | 0,12 | 0,00 | 0,53 | 0,77 | 0,69 | 70,20 |
| 14 | 101985734 | A | T | missense | 0,000003980 | 08/78 | 503 | Q/H | healthy | tail domain | 16,38 | -9,83 | 0,53 | 0,98 | 0,15 | 0,12 | 0,45 | 0,53 | 0,76 | 0,69 | 70,78 |
| 14 | 101985739 | T | C | missense | 0,000003980 | 08/78 | 505 | M/T | healthy | tail domain | 21,90 | 5,86 | 0,35 | 1,00 | 0,27 | 0,66 | 0,00 | 0,53 | 0,77 | 0,69 | 70,09 |
| 14 | 101985761 | T | G | missense | 0,000003980 | 08/78 | 512 | F/L | healthy | tail domain | 15,86 | -2,19 | 0,40 | 1,00 | 0,18 | 0,82 | 0,01 | 0,59 | 0,79 | 0,75 | 80,16 |
| 14 | 101985796 | A | T | missense | 0,000003980 | 08/78 | 524 | N/I | healthy | tail domain | 28,30 | 5,75 | 0,61 | 1,00 | 0,46 | 0,03 | 0,99 | 0,23 | 0,78 | 0,30 | 10,05 |
| 14 | 101985798 | C | A | missense | 0,000003980 | 08/78 | 525 | L/I | healthy | tail domain | 21,90 | 5,75 | 0,35 | 1,00 | 0,10 | 0,20 | 0,16 | 0,23 | 0,77 | 0,30 | 10,24 |
| 14 | 101985853 | C | T | missense | 0,000011900 | 08/78 | 543 | T/M | healthy | tail domain | 20,60 | 5,79 | 0,45 | 1,00 | 0,15 | 0,09 | 0,03 | 0,17 | 0,78 | 0,22 | 4,13 |
| 14 | 101985892 | G | A | missense | 0,000007960 | 08/78 | 556 | R/K | healthy | tail domain | 23,90 | 5,67 | 0,54 | 1,00 | 0,18 | 0,09 | 0,39 | 0,40 | 0,76 | 0,53 | 39,57 |
| 14 | 101985925 | G | A | missense | 0,000003980 | 08/78 | 567 | R/H | patient | tail domain | 24,20 | 5,85 | 0,68 | 1,00 | 0,19 | 0,14 | 0,08 | 0,55 | 0,77 | 0,70 | 72,82 |
| 14 | 101985927 | C | A | missense | 0,000000000 | 08/78 | 568 | L/I | patient | tail domain | 25,00 | 5,85 | 0,67 | 1,00 | 0,38 | 0,00 | 0,99 | 0,55 | 0,78 | 0,70 | 72,82 |
| 14 | 101985931 | G | A | missense | 0,000003980 | 08/78 | 569 | R/Q | healthy | tail domain | 31,00 | 5,85 | 0,58 | 1,00 | 0,44 | 0,00 | 0,99 | 0,60 | 0,78 | 0,77 | 83,82 |
| 14 | 101985946 | C | T | missense | 0,000003980 | 08/78 | 574 | T/I | healthy | tail domain | 24,00 | 5,85 | 0,54 | 1,00 | 0,27 | 0,02 | 0,06 | 0,55 | 0,77 | 0,71 | 74,41 |
| 14 | 101985955 | A | T | missense | 0,000003990 | 08/78 | 577 | N/I | healthy | tail domain | 29,50 | 5,85 | 0,68 | 1,00 | 0,68 | 0,00 | 1,00 | 0,55 | 0,76 | 0,72 | 75,25 |
| 14 | 101985961 | A | G | missense | 0,000003990 | 08/78 | 579 | N/S | healthy | tail domain | 24,30 | 5,85 | 0,46 | 1,00 | 0,38 | 0,19 | 0,58 | 0,50 | 0,75 | 0,66 | 66,02 |
| 14 | 101985963 | G | A | missense | 0,000000000 | 08/78 | 580 | E/K | patient | tail domain | 33,00 | 5,85 | 0,59 | 1,00 | 0,63 | 0,00 | 0,99 | 0,50 | 0,75 | 0,67 | 66,50 |
| 14 | 101985966 | A | T | missense | 0,000000000 | 08/78 | 581 | M/L | patient | tail domain | 27,30 | 5,85 | 0,75 | 1,00 | 0,80 | 0,00 | 1,00 | 0,55 | 0,75 | 0,72 | 76,85 |
| 14 | 101985985 | G | A | missense | 0,000003990 | 08/78 | 587 | R/K | healthy | tail domain | 24,00 | 5,85 | 0,52 | 1,00 | 0,14 | 1,00 | 0,62 | 0,50 | 0,77 | 0,65 | 63,70 |
| 14 | 101986018 | G | A | missense | 0,000000000 | 08/78 | 598 | R/H, R/L | patient | tail domain | 33,00 | 5,85 | 0,77 | 1,00 | 0,74 | 0,00 | 0,99 | 0,39 | 0,78 | 0,50 | 33,94 |
| 14 | 101986027 | T | A | missense | 0,000000000 | 08/78 | 601 | I/N | patient | tail domain | 29,90 | 5,85 | 0,85 | 1,00 | 0,78 | 0,00 | 1,00 | 0,39 | 0,78 | 0,49 | 33,74 |
| 14 | 101986030 | G | A | missense | 0,000003990 | 08/78 | 602 | R/H | healthy | tail domain | 24,50 | 5,85 | 0,61 | 1,00 | 0,28 | 0,17 | 0,39 | 0,39 | 0,78 | 0,50 | 34,11 |
| 14 | 101986029 | C | T | missense | 0,000015900 | 08/78 | 602 | R/C | healthy | tail domain | 29,30 | 5,85 | 0,67 | 1,00 | 0,47 | 0,01 | 0,98 | 0,39 | 0,78 | 0,50 | 34,11 |
| 14 | 101986057 | G | A | missense | 0,000003980 | 08/78 | 611 | R/H | healthy | tail domain | 27,30 | 5,85 | 0,60 | 1,00 | 0,38 | 0,15 | 0,98 | 0,36 | 0,78 | 0,46 | 28,15 |
| 14 | 101986086 | G | A | missense | 0,000027900 | 08/78 | 621 | D/N | healthy | tail domain | 25,70 | 5,85 | 0,45 | 1,00 | 0,17 | 0,03 | 0,07 | 0,31 | 0,80 | 0,38 | 17,50 |
| 14 | 101986094 | C | G | missense | 0,000000000 | 08/78 | 623 | F/L | patient | tail domain | 25,20 | 4,96 | 0,86 | 1,00 | 0,53 | 0,04 | 1,00 | NA | NA |  | NA |
| 14 | 101986112 | G | T | missense | 0,000035800 | 08/78 | 629 | Q/H | healthy | tail domain | 14,25 | -0,95 | 0,47 | 1,00 | 0,29 | 1,00 | 0,01 | NA | NA |  | NA |
| 14 | 101986137 | G | A | missense | 0,000003980 | 08/78 | 638 | V/I | healthy | tail domain | 23,30 | 5,85 | 0,48 | 1,00 | 0,17 | 0,07 | 0,07 | 0,44 | 0,79 | 0,57 | 46,37 |
| 14 | 101986152 | C | G | missense | 0,000003980 | 08/78 | 643 | P/A | healthy | tail domain | 24,40 | 5,85 | 0,61 | 1,00 | 0,41 | 0,04 | 0,72 | 0,47 | 0,79 | 0,60 | 53,30 |
| 14 | 101986158 | T | A | missense | 0,000003980 | 08/78 | 645 | S/T | healthy | tail domain | 26,40 | 5,85 | 0,59 | 1,00 | 0,35 | 0,04 | 0,91 | 0,47 | 0,78 | 0,61 | 55,03 |
| 14 | 101986164 | T | G | missense | 0,000003980 | 08/78 | 647 | S/A | healthy | tail domain | 21,30 | 5,85 | 0,51 | 1,00 | 0,13 | 1,00 | 0,12 | 0,47 | 0,78 | 0,61 | 54,53 |
| 14 | 101986167 | A | G | missense | 0,000019900 | 08/78 | 648 | I/V | healthy | tail domain | 24,30 | 5,85 | 0,69 | 1,00 | 0,34 | 0,15 | 0,86 | 0,47 | 0,78 | 0,61 | 54,81 |
| 14 | 101986172 | C | G | missense | 0,000003980 | 08/78 | 649 | I/M | healthy | tail domain | 23,10 | 4,05 | 0,63 | 1,00 | 0,33 | 0,07 | 0,67 | 0,50 | 0,78 | 0,64 | 61,67 |
| 14 | 101986179 | A | G | missense | 0,000003980 | 08/78 | 652 | K/E | healthy | tail domain | 25,50 | 5,85 | 0,56 | 1,00 | 0,47 | 0,00 | 0,34 | NA | NA |  | NA |
| 14 | 101986188 | G | A | missense | 0,000015900 | 08/78 | 655 | D/N | healthy | tail domain | 31,00 | 5,85 | 0,50 | 1,00 | 0,30 | 0,01 | 0,89 | 0,50 | 0,77 | 0,65 | 62,62 |
| 14 | 101986201 | C | T | missense | 0,000011900 | 08/78 | 659 | T/M | healthy | tail domain | 25,60 | 4,72 | 0,00 | 1,00 | 0,31 | 0,03 | 0,91 | 0,47 | 0,76 | 0,62 | 55,93 |
| 14 | 101986223 | A | T | missense | 0,000000000 | 08/78 | 666 | E/D | study patient | tail domain | 21,40 | -1,11 | 0,49 | 1,00 | 0,31 | 0,03 | 0,43 | 0,35 | 0,78 | 0,45 | 27,57 |
| 14 | 101986227 | G | A | missense | 0,000003980 | 08/78 | 668 | V/I | healthy | tail domain | 25,10 | 5,61 | 0,42 | 1,00 | 0,31 | 0,11 | 1,00 | 0,33 | 0,79 | 0,42 | 22,27 |
| 14 | 101986236 | A | G | missense | 0,000000000 | 08/78 | 671 | K/E | patient | tail domain | 23,30 | 5,61 | 0,48 | 1,00 | 0,28 | 0,35 | 0,06 | 0,43 | 0,79 | 0,54 | 41,58 |
| 14 | 101986254 | G | A | missense | 0,000027900 | 08/78 | 677 | V/M | healthy | tail domain | 28,90 | 5,61 | 0,40 | 1,00 | 0,22 | 0,04 | 0,80 | 0,50 | 0,79 | 0,63 | 58,55 |
| 14 | 101986267 | A | T | missense | 0,000003990 | 08/78 | 681 | K/M | healthy | tail domain | 31,00 | 5,61 | 0,47 | 1,00 | 0,60 | 0,00 | 0,96 | 0,44 | 0,81 | 0,54 | 41,36 |
| 14 | 101986279 | A | T | missense | 0,000003990 | 08/78 | 685 | D/V | healthy | tail domain | 25,60 | 5,71 | 0,00 | 1,00 | 0,65 | 0,16 | 0,68 | 0,47 | 0,81 | 0,58 | 48,40 |
| 14 | 101986279 | A | G | missense | 0,000003990 | 08/78 | 685 | D/G | healthy | tail domain | 30,00 | 5,71 | 0,57 | 1,00 | 0,67 | 0,03 | 0,91 | 0,47 | 0,81 | 0,58 | 48,40 |
| 14 | 101986293 | C | T | missense | 0,000003990 | 08/78 | 690 | R/C | healthy | tail domain | 28,60 | 5,71 | 0,66 | 1,00 | 0,40 | 0,00 | 0,99 | 0,50 | 0,82 | 0,61 | 55,54 |
| 14 | 101986296 | A | C | missense | 0,000019900 | 08/78 | 691 | M/L | healthy | tail domain | 22,70 | 5,71 | 0,46 | 1,00 | 0,23 | 0,26 | 0,00 | 0,54 | 0,82 | 0,66 | 65,59 |
| 14 | 101986315 | A | C | missense | 0,000003980 | 08/78 | 697 | E/A | healthy | tail domain | 23,20 | 5,71 | 0,48 | 1,00 | 0,22 | 0,55 | 0,05 | 0,64 | 0,79 | 0,81 | 88,75 |
| 14 | 101986359 | G | A | missense | 0,000003980 | 08/78 | 712 | G/S | healthy | tail domain | 22,40 | 5,71 | 0,44 | 1,00 | 0,17 | 1,00 | 0,05 | 0,63 | 0,75 | 0,84 | 91,59 |
| 14 | 101986360 | G | C | missense | 0,000007950 | 08/78 | 712 | G/A | healthy | tail domain | 22,90 | 5,71 | 0,50 | 1,00 | 0,15 | 0,43 | 0,05 | 0,63 | 0,75 | 0,84 | 91,59 |
| 14 | 101986366 | C | T | missense | 0,000003980 | 08/78 | 714 | S/L | healthy | tail domain | 25,50 | 5,71 | 0,62 | 1,00 | 0,34 | 0,01 | 0,45 | 0,61 | 0,75 | 0,81 | 89,54 |
| 14 | 101986372 | G | A | missense | 0,000023900 | 08/78 | 716 | R/H | healthy | tail domain | 25,20 | 5,71 | 0,56 | 1,00 | 0,25 | 0,01 | 0,09 | 0,63 | 0,75 | 0,85 | 92,00 |
| 14 | 101986374 | A | G | missense | 0,000003980 | 08/78 | 717 | I/V | healthy | tail domain | 23,10 | 5,71 | 0,69 | 1,00 | 0,18 | 0,09 | 0,32 | 0,63 | 0,74 | 0,85 | 92,43 |
| 14 | 101986378 | T | G | missense | 0,000003980 | 08/78 | 718 | F/C | healthy | tail domain | 29,80 | 5,71 | 0,78 | 1,00 | 0,77 | 0,00 | 0,97 | 0,63 | 0,74 | 0,85 | 92,34 |
| 14 | 101986381 | C | T | missense | 0,000003980 | 08/78 | 719 | T/I | healthy | tail domain | 22,40 | 5,71 | 0,51 | 1,00 | 0,18 | 0,11 | 0,11 | 0,60 | 0,75 | 0,80 | 88,27 |
| 14 | 101986383 | A | G | missense | 0,000003980 | 08/78 | 720 | I/V | healthy | tail domain | 24,00 | 5,71 | 0,41 | 1,00 | 0,18 | 0,21 | 0,61 | 0,60 | 0,74 | 0,81 | 89,22 |
| 14 | 101986393 | C | T | missense | 0,000003980 | 08/78 | 723 | T/I | healthy | tail domain | 19,02 | 5,71 | 0,51 | 1,00 | 0,13 | 0,41 | 0,01 | 0,57 | 0,73 | 0,78 | 85,43 |
| 14 | 101986398 | G | A | missense | 0,000003980 | 08/78 | 725 | V/I | healthy | tail domain | 22,60 | 5,71 | 0,45 | 1,00 | 0,10 | 0,34 | 0,01 | 0,57 | 0,73 | 0,78 | 85,43 |
| 14 | 101986401 | C | T | missense | 0,000007950 | 08/78 | 726 | R/W | healthy | tail domain | 26,80 | 4,75 | 0,00 | 1,00 | 0,24 | 0,00 | 0,96 | NA | NA |  | NA |
| 14 | 101986417 | A | G | missense | 0,000003980 | 08/78 | 731 | N/S | healthy | tail domain | 19,18 | 4,57 | 0,50 | 1,00 | 0,12 | 0,14 | 0,01 | 0,58 | 0,71 | 0,81 | 89,22 |
| 14 | 101986452 | A | G | missense | 0,000003980 | 08/78 | 743 | I/V | healthy | tail domain | 22,20 | 5,71 | 0,63 | 1,00 | 0,15 | 0,49 | 0,13 | 0,44 | 0,71 | 0,62 | 56,85 |
| 14 | 101986458 | A | G | missense | 0,000003980 | 08/78 | 745 | T/A | healthy | tail domain | 24,30 | 5,71 | 0,41 | 1,00 | 0,34 | 0,38 | 0,87 | 0,43 | 0,72 | 0,60 | 52,40 |
| 14 | 101986497 | T | C | missense | 0,000003980 | 08/78 | 758 | F/L | healthy | tail domain | 29,90 | 5,60 | 0,65 | 1,00 | 0,46 | 0,00 | 0,80 | 0,44 | 0,75 | 0,59 | 51,24 |
| 14 | 101986501 | G | A | missense | 0,000003980 | 08/78 | 759 | R/H | healthy | tail domain | 33,00 | 5,60 | 0,62 | 1,00 | 0,54 | 0,02 | 1,00 | 0,41 | 0,75 | 0,55 | 43,08 |
| 14 | 101986503 | G | A | missense | 0,000023900 | 08/78 | 760 | V/I | healthy | tail domain | 23,40 | 5,60 | 0,67 | 1,00 | 0,28 | 0,19 | 0,44 | 0,41 | 0,74 | 0,55 | 44,24 |
| 14 | 101986509 | C | G | missense | 0,000003980 | 08/78 | 762 | L/V | healthy | tail domain | 21,90 | 3,45 | 0,51 | 1,00 | 0,28 | 0,47 | 0,68 | 0,38 | 0,74 | 0,50 | 35,44 |
| 14 | 101986513 | C | T | missense | 0,000003980 | 08/78 | 763 | A/V | healthy | tail domain | 25,90 | 5,60 | 0,55 | 1,00 | 0,22 | 0,05 | 0,45 | 0,33 | 0,74 | 0,45 | 27,27 |
| 14 | 101986530 | C | T | missense | 0,000003980 | 08/78 | 769 | H/Y | healthy | tail domain | 25,40 | 5,60 | 0,43 | 1,00 | 0,50 | 0,02 | 0,93 | 0,32 | 0,75 | 0,42 | 22,68 |
| 14 | 101986552 | C | T | missense | 0,000000000 | 08/78 | 776 | P/L | patient | tail domain | 26,90 | 5,60 | 0,55 | 1,00 | 0,57 | 0,00 | 1,00 | 0,30 | 0,75 | 0,41 | 20,77 |
| 14 | 101986576 | G | A | missense | 0,000007950 | 08/78 | 784 | S/N | healthy | tail domain | 23,90 | 5,60 | 0,65 | 1,00 | 0,32 | 0,05 | 0,32 | 0,39 | 0,76 | 0,51 | 36,13 |
| 14 | 101986578 | G | A | missense | 0,000055700 | 08/78 | 785 | V/I | healthy | tail domain | 21,80 | 5,60 | 0,55 | 1,00 | 0,17 | 0,30 | 0,06 | 0,35 | 0,76 | 0,46 | 28,15 |
| 14 | 101986587 | T | C | missense | 0,000019900 | 08/78 | 788 | Y/H | healthy | tail domain | 25,50 | 5,60 | 0,84 | 1,00 | 0,74 | 0,00 | 0,99 | 0,35 | 0,75 | 0,46 | 28,75 |
| 14 | 101986594 | G | A | missense | 0,000003980 | 08/78 | 790 | R/Q | healthy | tail domain | 21,10 | 5,71 | 0,00 | 1,00 | 0,17 | 1,00 | 0,03 | 0,39 | 0,75 | 0,52 | 38,39 |
| 14 | 101986599 | T | G | missense | 0,000015900 | 08/78 | 792 | C/G | healthy | tail domain | 23,60 | 5,71 | 0,66 | 1,00 | 0,45 | 0,27 | 0,17 | 0,42 | 0,75 | 0,56 | 46,07 |
| 14 | 101986602 | G | A | missense | 0,000003980 | 08/78 | 793 | E/K | healthy | tail domain | 23,90 | 5,71 | 0,48 | 1,00 | 0,18 | 0,48 | 0,27 | NA | NA |  | NA |
| 14 | 101986609 | T | G | missense | 0,000003980 | 08/78 | 795 | V/G | healthy | tail domain | 29,10 | 5,71 | 0,85 | 1,00 | 0,70 | 0,00 | 0,83 | 0,48 | 0,74 | 0,64 | 61,67 |
| 14 | 101986618 | G | A | missense | 0,000003980 | 08/78 | 798 | R/Q | healthy | tail domain | 24,00 | 5,82 | 0,60 | 1,00 | 0,20 | 0,57 | 0,35 | 0,54 | 0,74 | 0,73 | 78,22 |
| 14 | 101986617 | C | T | missense | 0,000007960 | 08/78 | 798 | R/W | healthy | tail domain | 27,70 | 5,82 | 0,66 | 1,00 | 0,38 | 0,03 | 0,96 | NA | NA |  | NA |
| 14 | 101986636 | T | C | missense | 0,000003980 | 08/78 | 804 | L/S | healthy | tail domain | 28,50 | 5,52 | 0,89 | 1,00 | 0,91 | 0,00 | 1,00 | NA | NA |  | NA |
| 14 | 101986639 | T | C | missense | 0,000003980 | 08/78 | 805 | V/A | healthy | tail domain | 24,60 | 5,52 | 0,68 | 1,00 | 0,32 | 0,02 | 0,19 | 0,62 | 0,75 | 0,83 | 90,98 |
| 14 | 101986660 | T | C | missense | 0,000003980 | 08/78 | 812 | V/A | healthy | tail domain | 23,60 | 5,52 | 0,77 | 1,00 | 0,27 | 0,17 | 0,11 | 0,63 | 0,76 | 0,82 | 90,42 |
| 14 | 101986663 | A | G | missense | 0,000003980 | 08/78 | 813 | Q/R | healthy | tail domain | 25,10 | 5,52 | 0,50 | 1,00 | 0,30 | 0,12 | 0,90 | 0,67 | 0,75 | 0,89 | 94,56 |
| 14 | 101986666 | C | G | missense | 0,000007970 | 08/78 | 814 | A/G | healthy | tail domain | 21,60 | 5,41 | 0,00 | 1,00 | 0,08 | 0,35 | 0,00 | 0,57 | 0,75 | 0,76 | 82,14 |
| 14 | 101986671 | A | G | missense | 0,000003990 | 08/78 | 816 | I/V | healthy | tail domain | 19,52 | 4,37 | 0,65 | 1,00 | 0,11 | 0,17 | 0,02 | 0,60 | 0,75 | 0,80 | 87,69 |
| 14 | 101986674 | G | A | missense | 0,000019900 | 08/78 | 817 | A/T | healthy | tail domain | 22,90 | 5,52 | 0,34 | 1,00 | 0,08 | 0,39 | 0,01 | 0,56 | 0,75 | 0,76 | 81,54 |
| 14 | 101986687 | C | T | missense | 0,000003990 | 08/78 | 821 | A/V | healthy | tail domain | 22,90 | 5,52 | 0,00 | 1,00 | 0,14 | 0,09 | 0,05 | 0,53 | 0,75 | 0,71 | 74,52 |
| 14 | 101986717 | C | A | missense | 0,000004000 | 08/78 | 831 | P/Q | healthy | tail domain | 21,80 | 5,52 | 0,45 | 1,00 | 0,26 | 0,56 | 0,06 | 0,44 | 0,76 | 0,58 | 50,03 |
| 14 | 101986727 | G | T | missense | 0,000004000 | 08/78 | 834 | Q/H | healthy | tail domain | 23,70 | 3,68 | 0,42 | 1,00 | 0,19 | 0,07 | 0,75 | NA | NA |  | NA |
| 14 | 101986741 | C | G | missense | 0,000004010 | 08/78 | 839 | T/S | healthy | tail domain | 18,60 | 4,63 | 0,37 | 1,00 | 0,06 | 0,55 | 0,00 | 0,50 | 0,77 | 0,65 | 62,26 |
| 14 | 101986743 | G | A | missense | 0,000004010 | 08/78 | 840 | V/I | healthy | tail domain | 23,00 | 5,52 | 0,30 | 1,00 | 0,15 | 0,18 | 0,04 | 0,50 | 0,78 | 0,64 | 61,09 |
| 14 | 101986762 | A | T | missense | 0,000004010 | 08/78 | 846 | K/M | healthy | tail domain | 34,00 | 5,52 | 0,44 | 1,00 | 0,41 | 0,00 | 0,96 | 0,53 | 0,77 | 0,69 | 71,12 |
| 14 | 101987471 | A | G | missense | 0,000003980 | 09/78 | 853 | I/V | healthy | tail domain | 22,30 | 5,78 | 0,37 | 1,00 | 0,19 | 0,47 | 0,00 | 0,58 | 0,78 | 0,74 | 79,97 |
| 14 | 101987472 | T | C | missense | 0,000007960 | 09/78 | 853 | I/T | healthy | tail domain | 23,60 | 5,78 | 0,48 | 1,00 | 0,15 | 0,07 | 0,01 | 0,58 | 0,78 | 0,74 | 79,97 |
| 14 | 101987492 | G | C | missense | 0,000003980 | 09/78 | 860 | E/Q | healthy | tail domain | 23,20 | 5,78 | 0,34 | 1,00 | 0,24 | 0,33 | 0,01 | NA | NA |  | NA |
| 14 | 101987516 | A | G | missense | 0,000003980 | 09/78 | 868 | M/V | healthy | tail domain | 16,14 | 3,41 | 0,30 | 1,00 | 0,09 | 0,06 | 0,00 | 0,60 | 0,79 | 0,76 | 82,89 |
| 14 | 101987526 | A | G | missense | 0,000007960 | 09/78 | 871 | H/R | healthy | tail domain | 20,90 | 4,62 | 0,00 | 1,00 | 0,16 | 0,45 | 0,03 | 0,56 | 0,79 | 0,71 | 73,38 |
| 14 | 101987538 | C | T | missense | 0,000015900 | 09/78 | 875 | S/L | healthy | tail domain | 21,10 | 5,78 | 0,00 | 1,00 | 0,13 | 0,25 | 0,00 | 0,50 | 0,79 | 0,63 | 59,26 |
| 14 | 101987564 | G | T | missense | 0,000003980 | 09/78 | 884 | A/S | healthy | tail domain | 23,20 | 5,78 | 0,39 | 1,00 | 0,27 | 0,21 | 0,01 | 0,55 | 0,80 | 0,69 | 69,64 |
| 14 | 101987586 | A | G | missense | 0,000003980 | 09/78 | 891 | H/R | healthy | tail domain | 22,60 | 5,78 | 0,00 | 1,00 | 0,22 | 0,23 | 0,00 | 0,50 | 0,79 | 0,63 | 57,99 |
| 14 | 101987585 | C | T | missense | 0,000023900 | 09/78 | 891 | H/Y | healthy | tail domain | 23,10 | 5,78 | 0,36 | 1,00 | 0,11 | 0,05 | 0,00 | 0,50 | 0,79 | 0,63 | 57,99 |
| 14 | 101987598 | A | G | missense | 0,000003980 | 09/78 | 895 | N/S | healthy | tail domain | 26,20 | 5,78 | 0,39 | 1,00 | 0,41 | 0,00 | 0,98 | 0,44 | 0,78 | 0,57 | 48,10 |
| 14 | 101988721 | G | A | missense | 0,000043800 | 10/78 | 913 | V/I | healthy | tail domain | 23,40 | 5,68 | 0,00 | 1,00 | 0,10 | 0,19 | 0,01 | 0,43 | 0,76 | 0,56 | 45,90 |
| 14 | 101988752 | C | T | missense | 0,000031800 | 10/78 | 923 | T/M | healthy | tail domain | 25,90 | 5,68 | 0,51 | 1,00 | 0,32 | 0,04 | 0,83 | 0,67 | 0,76 | 0,88 | 94,23 |
| 14 | 101988757 | G | C | missense | 0,000015900 | 10/78 | 925 | V/L | healthy | tail domain | 23,50 | 5,68 | 0,46 | 1,00 | 0,18 | 0,16 | 0,00 | 0,70 | 0,76 | 0,92 | 95,91 |
| 14 | 101988769 | C | A | missense | 0,000003980 | 10/78 | 929 | Q/K | healthy | tail domain | 21,40 | 5,68 | 0,48 | 1,00 | 0,27 | 0,71 | 0,00 | 0,60 | 0,76 | 0,80 | 87,00 |
| 14 | 101988779 | A | T | missense | 0,000003980 | 10/78 | 932 | D/V | healthy | tail domain | 29,70 | 5,68 | 0,45 | 1,00 | 0,40 | 0,03 | 0,99 | 0,60 | 0,75 | 0,80 | 87,41 |
| 14 | 101988784 | G | A | missense | 0,000003980 | 10/78 | 934 | A/T | healthy | tail domain | 22,50 | 4,78 | 0,35 | 1,00 | 0,22 | 0,55 | 0,08 | 0,67 | 0,76 | 0,88 | 94,41 |
| 14 | 101988802 | A | C | missense | 0,000003980 | 10/78 | 940 | T/P | healthy | tail domain | 22,50 | 5,68 | 0,23 | 1,00 | 0,29 | 0,16 | 0,00 | 0,63 | 0,78 | 0,81 | 88,40 |
| 14 | 101991533 | G | A | missense | 0,000039800 | 11/78 | 959 | V/I | healthy | tail domain | 21,60 | 3,17 | 0,00 | 1,00 | 0,19 | 0,29 | 0,00 | 0,20 | 0,78 | 0,26 | 6,56 |
| 14 | 101991567 | A | G | missense | 0,000000000 | 11/78 | 970 | Y/C | patient | tail domain | 28,90 | 4,71 | 0,46 | 1,00 | 0,72 | 0,01 | 0,78 | 0,30 | 0,79 | 0,38 | 17,09 |
| 14 | 101991583 | T | G | missense | 0,000003980 | 11/78 | 975 | I/M | healthy | tail domain | 11,76 | -5,23 | 0,46 | 1,00 | 0,49 | 0,09 | 0,05 | 0,22 | 0,79 | 0,28 | 9,02 |
| 14 | 101991584 | G | A | missense | 0,000003980 | 11/78 | 976 | E/K | healthy | tail domain | 31,00 | 5,85 | 0,54 | 1,00 | 0,69 | 0,01 | 0,52 | 0,30 | 0,78 | 0,38 | 17,39 |
| 14 | 101991630 | T | C | missense | 0,000003980 | 11/78 | 991 | M/T | healthy | tail domain | 21,30 | 3,16 | 0,55 | 0,97 | 0,14 | 0,09 | 0,00 | 0,46 | 0,79 | 0,58 | 50,03 |
| 14 | 101991635 | G | A | missense | 0,000047700 | 11/78 | 993 | V/I | healthy | tail domain | 17,96 | 3,86 | 0,33 | 0,86 | 0,19 | 0,54 | 0,01 | 0,50 | 0,79 | 0,64 | 59,72 |
| 14 | 101991641 | T | C | missense | 0,000003980 | 11/78 | 995 | S/P | healthy | tail domain | 25,00 | 5,85 | 0,55 | 1,00 | 0,66 | 0,07 | 0,55 | 0,60 | 0,80 | 0,75 | 81,26 |
| 14 | 101991648 | C | T | missense | 0,000000000 | 11/78 | 997 | P/L | healthy | tail domain | 23,40 | 5,85 | 0,45 | 1,00 | 0,48 | 0,07 | 0,07 | 0,54 | 0,80 | 0,67 | 68,20 |
| 14 | 101991657 | A | G | missense | 0,000003980 | 11/78 | 1000 | Q/R | healthy | tail domain | 23,50 | 5,85 | 0,30 | 1,00 | 0,46 | 0,27 | 0,01 | 0,50 | 0,79 | 0,63 | 58,23 |
| 14 | 101994194 | A | C | missense | 0,000003980 | 12/78 | 1009 | H/P | healthy | tail domain | 23,40 | 5,78 | 0,49 | 1,00 | 0,42 | 0,23 | 0,00 | 0,47 | 0,78 | 0,60 | 53,30 |
| 14 | 101994203 | T | C | missense | 0,000003980 | 12/78 | 1012 | L/S | healthy | tail domain | 23,40 | 5,78 | 0,61 | 1,00 | 0,28 | 0,74 | 0,01 | 0,53 | 0,76 | 0,70 | 72,37 |
| 14 | 101994206 | C | T | missense | 0,000003980 | 12/78 | 1013 | T/I | healthy | tail domain | 23,40 | 4,87 | 0,37 | 1,00 | 0,22 | 0,04 | 0,01 | 0,53 | 0,75 | 0,70 | 72,65 |
| 14 | 101994214 | G | C | missense | 0,000067600 | 12/78 | 1016 | E/Q | healthy | tail domain | 23,50 | 5,78 | 0,33 | 1,00 | 0,26 | 0,34 | 0,01 | NA | NA |  | NA |
| 14 | 101994242 | G | A | missense | 0,000007950 | 12/78 | 1025 | R/Q | healthy | tail domain | 23,80 | 5,78 | 0,48 | 1,00 | 0,16 | 0,26 | 0,02 | 0,50 | 0,75 | 0,67 | 66,30 |
| 14 | 101994241 | C | T | missense | 0,000083500 | 12/78 | 1025 | R/W | healthy | tail domain | 25,20 | 4,88 | 0,53 | 1,00 | 0,29 | 0,01 | 0,72 | NA | NA |  | NA |
| 14 | 101994247 | C | T | missense | 0,000007950 | 12/78 | 1027 | P/S | healthy | tail domain | 24,40 | 5,78 | 0,00 | 1,00 | 0,44 | 0,13 | 0,87 | 0,56 | 0,75 | 0,74 | 79,45 |
| 14 | 101994252 | T | G | missense | 0,000015900 | 12/78 | 1028 | D/E | healthy | tail domain | 8,62 | -1,94 | 0,24 | 1,00 | 0,37 | 0,45 | 0,00 | NA | NA |  | NA |
| 14 | 101994256 | C | T | missense | 0,000003980 | 12/78 | 1030 | P/S | healthy | tail domain | 19,66 | 5,78 | 0,30 | 1,00 | 0,30 | 0,96 | 0,00 | 0,62 | 0,76 | 0,81 | 88,98 |
| 14 | 101994259 | G | T | missense | 0,000003980 | 12/78 | 1031 | V/F | healthy | tail domain | 21,50 | 4,83 | 0,29 | 1,00 | 0,17 | 0,04 | 0,02 | 0,57 | 0,76 | 0,76 | 81,54 |
| 14 | 101994289 | A | C | missense | 0,000003980 | 12/78 | 1041 | M/L | healthy | tail domain | 22,70 | 4,44 | 0,42 | 0,62 | 0,22 | 0,06 | 0,00 | 0,57 | 0,76 | 0,75 | 80,81 |
| 14 | 101994310 | G | A | missense | 0,000003980 | 12/78 | 1048 | E/K | healthy | tail domain | 23,20 | 5,78 | 0,46 | 1,00 | 0,32 | 0,48 | 0,00 | NA | NA |  | NA |
| 14 | 101994314 | A | G | missense | 0,000003980 | 12/78 | 1049 | Q/R | healthy | tail domain | 22,20 | 5,78 | 0,33 | 1,00 | 0,30 | 0,35 | 0,00 | 0,42 | 0,78 | 0,53 | 40,13 |
| 14 | 101994323 | A | G | missense | 0,000003980 | 12/78 | 1052 | K/R | healthy | tail domain | 24,50 | 5,78 | 0,44 | 1,00 | 0,37 | 0,19 | 0,16 | 0,50 | 0,79 | 0,63 | 59,09 |
| 14 | 101994701 | A | T | missense | 0,000003980 | 13/78 | 1062 | D/V | healthy | tail domain | 28,60 | 5,73 | 0,54 | 1,00 | 0,81 | 0,02 | 0,79 | 0,62 | 0,80 | 0,77 | 83,39 |
| 14 | 101994710 | C | T | missense | 0,000003980 | 13/78 | 1065 | A/V | healthy | tail domain | 23,00 | 5,73 | 0,37 | 1,00 | 0,29 | 0,11 | 0,02 | 0,60 | 0,80 | 0,75 | 80,16 |
| 14 | 101994734 | G | A | missense | 0,000019900 | 13/78 | 1073 | G/E | healthy | tail domain | 24,90 | 5,73 | 0,41 | 1,00 | 0,44 | 0,04 | 0,13 | 0,50 | 0,80 | 0,63 | 57,65 |
| 14 | 101994736 | G | A | missense | 0,000003980 | 13/78 | 1074 | E/K | healthy | tail domain | 24,40 | 5,73 | 0,42 | 1,00 | 0,44 | 0,08 | 0,00 | NA | NA |  | NA |
| 14 | 101994746 | A | G | missense | 0,000015900 | 13/78 | 1077 | N/S | healthy | tail domain | 15,85 | -0,81 | 0,28 | 1,00 | 0,16 | 1,00 | 0,00 | 0,50 | 0,80 | 0,63 | 57,22 |
| 14 | 101994757 | G | T | missense | 0,000003980 | 13/78 | 1081 | A/S | healthy | tail domain | 23,30 | 5,73 | 0,31 | 1,00 | 0,24 | 0,05 | 0,02 | 0,40 | 0,80 | 0,50 | 34,93 |
| 14 | 101994758 | C | T | missense | 0,000003980 | 13/78 | 1081 | A/V | healthy | tail domain | 23,70 | 5,73 | 0,40 | 1,00 | 0,26 | 0,02 | 0,03 | 0,40 | 0,80 | 0,50 | 34,93 |
| 14 | 101994772 | A | G | missense | 0,000003980 | 13/78 | 1086 | I/V | healthy | tail domain | 24,30 | 5,73 | 0,47 | 1,00 | 0,41 | 0,06 | 0,60 | 0,50 | 0,78 | 0,64 | 61,24 |
| 14 | 101994794 | T | C | missense | 0,000000000 | 13/78 | 1093 | F/S | patient | tail domain | 33,00 | 5,73 | 0,62 | 1,00 | 0,82 | 0,00 | 0,98 | 0,43 | 0,77 | 0,55 | 44,44 |
| 14 | 101994812 | A | G | missense | 0,000015900 | 13/78 | 1099 | K/R | healthy | tail domain | 19,80 | 5,73 | 0,31 | 1,00 | 0,26 | 0,96 | 0,00 | 0,36 | 0,77 | 0,47 | 29,68 |
| 14 | 101994818 | A | C | missense | 0,000003980 | 13/78 | 1101 | E/A | healthy | tail domain | 23,20 | 5,73 | 0,33 | 1,00 | 0,38 | 0,55 | 0,00 | 0,44 | 0,77 | 0,58 | 48,72 |
| 14 | 101994829 | G | A | missense | 0,000003990 | 13/78 | 1105 | V/I | healthy | tail domain | 22,00 | 5,73 | 0,39 | 1,00 | 0,34 | 0,83 | 0,00 | 0,38 | 0,79 | 0,48 | 30,41 |
| 14 | 101995022 | C | T | missense | 0,000000000 | 14/78 | 1124 | H/Y | patient | tail domain | 26,10 | 5,84 | 0,41 | 1,00 | 0,64 | 0,00 | 0,98 | 0,46 | 0,79 | 0,57 | 48,10 |
| 14 | 101995061 | T | A | missense | 0,000003980 | 14/78 | 1137 | S/T | healthy | tail domain | 17,78 | 3,36 | 0,29 | 0,66 | 0,14 | 0,66 | 0,00 | 0,61 | 0,80 | 0,76 | 82,72 |
| 14 | 101995062 | C | T | missense | 0,000003980 | 14/78 | 1137 | S/L | healthy | tail domain | 22,70 | 4,90 | 0,38 | 0,99 | 0,20 | 0,13 | 0,01 | NA | NA |  | NA |
| 14 | 101995065 | A | G | missense | 0,000003980 | 14/78 | 1138 | N/S | healthy | tail domain | 20,70 | 3,50 | 0,31 | 1,00 | 0,20 | 0,32 | 0,00 | 0,61 | 0,80 | 0,77 | 83,58 |
| 14 | 101995067 | A | G | missense | 0,000003980 | 14/78 | 1139 | M/V | healthy | tail domain | 24,00 | 5,84 | 0,26 | 1,00 | 0,61 | 0,07 | 0,46 | 0,61 | 0,80 | 0,76 | 83,00 |
| 14 | 101995068 | T | C | missense | 0,000007950 | 14/78 | 1139 | M/T | healthy | tail domain | 26,90 | 5,84 | 0,43 | 1,00 | 0,78 | 0,02 | 0,91 | 0,61 | 0,80 | 0,76 | 83,00 |
| 14 | 101995071 | C | T | missense | 0,000039800 | 14/78 | 1140 | T/M | healthy | tail domain | 23,70 | 5,84 | 0,27 | 1,00 | 0,36 | 0,03 | 0,12 | 0,63 | 0,80 | 0,80 | 87,00 |
| 14 | 101995081 | T | G | missense | 0,000003980 | 14/78 | 1143 | H/Q | healthy | tail domain | 19,74 | -4,51 | 0,30 | 1,00 | 0,35 | 0,02 | 0,54 | 0,63 | 0,79 | 0,80 | 87,56 |
| 14 | 101995082 | T | A | missense | 0,000003980 | 14/78 | 1144 | S/T | healthy | tail domain | 20,60 | 3,39 | 0,22 | 1,00 | 0,06 | 0,59 | 0,00 | 0,63 | 0,78 | 0,81 | 88,75 |
| 14 | 101995087 | G | T | missense | 0,000003980 | 14/78 | 1145 | Q/H | healthy | tail domain | 23,50 | 4,94 | 0,33 | 1,00 | 0,19 | 0,05 | 0,00 | NA | NA |  | NA |
| 14 | 101995094 | A | G | missense | 0,000003980 | 14/78 | 1148 | K/E | healthy | tail domain | 24,60 | 5,84 | 0,22 | 1,00 | 0,30 | 0,13 | 0,06 | 0,65 | 0,77 | 0,84 | 91,59 |
| 14 | 101995184 | C | T | missense | 0,000007950 | 15/78 | 1150 | R/C | healthy | tail domain | 28,00 | 5,13 | 0,37 | 1,00 | 0,60 | 0,00 | 1,00 | 0,59 | 0,78 | 0,76 | 82,72 |
| 14 | 101995200 | A | G | missense | 0,000003980 | 15/78 | 1155 | Q/R | healthy | tail domain | 23,30 | 6,03 | 0,18 | 1,00 | 0,28 | 0,52 | 0,12 | 0,50 | 0,76 | 0,66 | 64,73 |
| 14 | 101995215 | C | T | missense | 0,000003980 | 15/78 | 1160 | T/M | healthy | tail domain | 25,50 | 6,03 | 0,20 | 1,00 | 0,32 | 0,00 | 0,56 | 0,46 | 0,76 | 0,60 | 53,91 |
| 14 | 101995251 | A | G | missense | 0,000003980 | 15/78 | 1172 | Y/C | healthy | tail domain | 27,70 | 5,85 | 0,43 | 1,00 | 0,39 | 0,05 | 0,78 | 0,27 | 0,78 | 0,35 | 14,20 |
| 14 | 101995269 | G | A | missense | 0,000003980 | 15/78 | 1178 | R/Q | healthy | tail domain | 33,00 | 5,85 | 0,32 | 1,00 | 0,51 | 0,03 | 0,66 | 0,42 | 0,76 | 0,55 | 42,59 |
| 14 | 101997041 | C | T | missense | 0,000020000 | 16/78 | 1191 | R/C | healthy | tail domain | 29,80 | 6,03 | 0,48 | 1,00 | 0,56 | 0,00 | 0,96 | 0,42 | 0,78 | 0,53 | 40,13 |
| 14 | 101997045 | A | G | missense | 0,000003990 | 16/78 | 1192 | N/S | healthy | tail domain | 22,00 | 6,03 | 0,28 | 1,00 | 0,21 | 0,62 | 0,01 | 0,42 | 0,79 | 0,53 | 39,38 |
| 14 | 101997044 | A | C | missense | 0,000003990 | 16/78 | 1192 | N/H | healthy | tail domain | 23,20 | 6,03 | 0,27 | 1,00 | 0,35 | 0,09 | 0,02 | 0,42 | 0,79 | 0,53 | 39,38 |
| 14 | 101997053 | C | T | missense | 0,000003980 | 16/78 | 1195 | R/C | healthy | tail domain | 33,00 | 6,03 | 0,48 | 1,00 | 0,52 | 0,00 | 0,94 | 0,36 | 0,79 | 0,46 | 28,34 |
| 14 | 101997073 | G | T | missense | 0,000000000 | 16/78 | 1201 | R/S | patient | tail domain | 23,50 | 1,14 | 0,00 | 1,00 | 0,51 | 0,00 | 1,00 | NA | NA |  | NA |
| 14 | 101997110 | A | G | missense | 0,000003980 | 16/78 | 1214 | I/V | healthy | tail domain | 20,70 | 4,89 | 0,34 | 1,00 | 0,24 | 1,00 | 0,03 | 0,38 | 0,79 | 0,48 | 30,41 |
| 14 | 101997115 | G | T | missense | 0,000003980 | 16/78 | 1215 | E/D | healthy | tail domain | 17,78 | 5,05 | 0,40 | 1,00 | 0,38 | 0,57 | 0,21 | 0,38 | 0,79 | 0,48 | 30,41 |
| 14 | 101997125 | G | A | missense | 0,000003980 | 16/78 | 1219 | G/R | healthy | tail domain | 26,50 | 6,03 | 0,40 | 1,00 | 0,43 | 0,20 | 0,78 | 0,46 | 0,79 | 0,59 | 50,38 |
| 14 | 101997140 | A | G | missense | 0,000003980 | 16/78 | 1224 | I/V | healthy | tail domain | 24,70 | 6,03 | 0,26 | 1,00 | 0,27 | 0,08 | 0,96 | 0,57 | 0,79 | 0,73 | 77,21 |
| 14 | 101997147 | G | A | missense | 0,000007950 | 16/78 | 1226 | R/Q | healthy | tail domain | 23,80 | 6,03 | 0,39 | 1,00 | 0,11 | 0,22 | 0,05 | 0,57 | 0,79 | 0,72 | 76,50 |
| 14 | 101997146 | C | T | missense | 0,000015900 | 16/78 | 1226 | R/W | healthy | tail domain | 27,40 | 5,14 | 0,00 | 1,00 | 0,31 | 0,00 | 0,77 | 0,57 | 0,79 | 0,72 | 76,50 |
| 14 | 101997174 | A | G | missense | 0,000003980 | 16/78 | 1235 | Q/R | healthy | tail domain | 28,60 | 6,03 | 0,29 | 1,00 | 0,42 | 0,01 | 0,97 | 0,71 | 0,78 | 0,92 | 95,87 |
| 14 | 101997179 | G | A | missense | 0,000003980 | 16/78 | 1237 | A/T | healthy | tail domain | 23,70 | 6,03 | 0,25 | 1,00 | 0,30 | 0,48 | 0,11 | 0,71 | 0,78 | 0,92 | 95,93 |
| 14 | 101997213 | G | A | missense | 0,000003980 | 16/78 | 1248 | R/Q | healthy | tail domain | 24,30 | 6,03 | 0,27 | 1,00 | 0,12 | 0,08 | 0,01 | 0,63 | 0,77 | 0,82 | 89,82 |
| 14 | 101997212 | C | T | missense | 0,000007960 | 16/78 | 1248 | R/W | healthy | tail domain | 27,70 | 4,03 | 0,48 | 0,99 | 0,30 | 0,00 | 0,79 | NA | NA |  | NA |
| 14 | 101997218 | G | T | missense | 0,000003980 | 16/78 | 1250 | V/L | healthy | tail domain | 23,70 | 6,03 | 0,45 | 1,00 | 0,35 | 0,21 | 0,26 | 0,63 | 0,76 | 0,82 | 90,30 |
| 14 | 101997219 | T | C | missense | 0,000003980 | 16/78 | 1250 | V/A | healthy | tail domain | 28,70 | 6,03 | 0,51 | 1,00 | 0,54 | 0,00 | 0,78 | 0,63 | 0,76 | 0,82 | 90,30 |
| 14 | 101997218 | G | A | missense | 0,000007960 | 16/78 | 1250 | V/M | healthy | tail domain | 29,20 | 6,03 | 0,00 | 1,00 | 0,42 | 0,03 | 0,72 | 0,63 | 0,76 | 0,82 | 90,30 |
| 14 | 101997236 | G | A | missense | 0,000003980 | 16/78 | 1256 | D/N | healthy | tail domain | 24,10 | 6,03 | 0,26 | 1,00 | 0,31 | 0,05 | 0,09 | 0,59 | 0,75 | 0,78 | 85,56 |
| 14 | 101997246 | C | G | missense | 0,000003990 | 16/78 | 1259 | T/S | healthy | tail domain | 9,87 | 3,03 | 0,21 | 1,00 | 0,08 | 0,60 | 0,00 | 0,62 | 0,75 | 0,82 | 89,89 |
| 14 | 101997257 | A | G | missense | 0,000016000 | 16/78 | 1263 | K/E | healthy | tail domain | 23,80 | 6,03 | 0,39 | 1,00 | 0,23 | 0,75 | 0,54 | NA | NA |  | NA |
| 14 | 101997269 | G | A | missense | 0,000004000 | 16/78 | 1267 | V/I | healthy | tail domain | 23,30 | 6,03 | 0,31 | 1,00 | 0,25 | 0,19 | 0,08 | 0,55 | 0,75 | 0,74 | 78,91 |
| 14 | 101997273 | C | T | missense | 0,000004000 | 16/78 | 1268 | T/M | healthy | tail domain | 22,00 | 5,14 | 0,46 | 0,99 | 0,07 | 0,18 | 0,00 | 0,52 | 0,74 | 0,71 | 73,14 |
| 14 | 101997273 | C | A | missense | 0,000008000 | 16/78 | 1268 | T/K | healthy | tail domain | 24,20 | 5,14 | 0,36 | 0,99 | 0,16 | 0,02 | 0,34 | 0,52 | 0,74 | 0,71 | 73,14 |
| 14 | 101999994 | C | A | missense | 0,000003980 | 17/78 | 1270 | N/K | healthy | tail domain | 10,19 | -1,07 | 0,37 | 0,92 | 0,17 | 0,27 | 0,01 | 0,55 | 0,74 | 0,74 | 79,45 |
| 14 | 101999993 | A | G | missense | 0,000007960 | 17/78 | 1270 | N/S | healthy | tail domain | 17,40 | 4,27 | 0,29 | 1,00 | 0,11 | 1,00 | 0,00 | 0,55 | 0,74 | 0,74 | 79,45 |
| 14 | 101999999 | G | T | missense | 0,000003980 | 17/78 | 1272 | R/L | healthy | tail domain | 24,60 | 5,43 | 0,39 | 1,00 | 0,38 | 0,12 | 0,19 | 0,58 | 0,75 | 0,77 | 84,12 |
| 14 | 102000001 | C | T | missense | 0,000003980 | 17/78 | 1273 | P/S | healthy | tail domain | 26,20 | 5,43 | 0,26 | 1,00 | 0,41 | 0,03 | 0,82 | 0,58 | 0,75 | 0,77 | 84,12 |
| 14 | 102000002 | C | T | missense | 0,000003980 | 17/78 | 1273 | P/L | healthy | tail domain | 27,90 | 5,43 | 0,38 | 1,00 | 0,57 | 0,00 | 0,99 | 0,58 | 0,75 | 0,77 | 84,12 |
| 14 | 102000043 | T | C | missense | 0,000003980 | 17/78 | 1287 | F/L | healthy | tail domain | 22,70 | 5,43 | 0,47 | 1,00 | 0,27 | 1,00 | 0,05 | 0,64 | 0,76 | 0,85 | 92,19 |
| 14 | 102000061 | G | A | missense | 0,000011900 | 17/78 | 1293 | D/N | healthy | tail domain | 29,20 | 5,43 | 0,00 | 1,00 | 0,25 | 0,01 | 0,46 | 0,64 | 0,76 | 0,84 | 91,59 |
| 14 | 102000067 | G | A | missense | 0,000003980 | 17/78 | 1295 | E/K | healthy | tail domain | 26,10 | 5,43 | 0,33 | 1,00 | 0,19 | 0,21 | 0,52 | NA | NA |  | NA |
| 14 | 102000074 | G | A | missense | 0,000003980 | 17/78 | 1297 | C/Y | healthy | tail domain | 25,80 | 5,43 | 0,39 | 1,00 | 0,41 | 0,00 | 0,36 | 0,71 | 0,77 | 0,93 | 96,36 |
| 14 | 102000076 | G | A | missense | 0,000003980 | 17/78 | 1298 | A/T | healthy | tail domain | 23,70 | 5,43 | 0,39 | 1,00 | 0,18 | 0,21 | 0,03 | 0,71 | 0,77 | 0,93 | 96,36 |
| 14 | 102000086 | A | G | missense | 0,000003980 | 17/78 | 1301 | K/R | healthy | tail domain | 23,80 | 5,43 | 0,28 | 1,00 | 0,30 | 0,08 | 0,27 | 0,79 | 0,77 | 1,02 | 98,73 |
| 14 | 102000092 | C | G | missense | 0,000003980 | 17/78 | 1303 | A/G | healthy | tail domain | 26,90 | 5,43 | 0,31 | 1,00 | 0,45 | 0,00 | 1,00 | 0,75 | 0,77 | 0,98 | 97,61 |
| 14 | 102000091 | G | A | missense | 0,000003980 | 17/78 | 1303 | A/T | healthy | tail domain | 31,00 | 5,43 | 0,28 | 1,00 | 0,47 | 0,00 | 1,00 | 0,75 | 0,77 | 0,98 | 97,61 |
| 14 | 102000106 | G | A | missense | 0,000003980 | 17/78 | 1308 | D/N | healthy | tail domain | 24,00 | 5,43 | 0,29 | 1,00 | 0,22 | 0,27 | 0,35 | 0,82 | 0,77 | 1,07 | 99,46 |
| 14 | 102000110 | C | T | missense | 0,000003980 | 17/78 | 1309 | T/I | healthy | tail domain | 19,88 | 4,53 | 0,27 | 1,00 | 0,14 | 0,26 | 0,01 | 0,81 | 0,77 | 1,05 | 99,35 |
| 14 | 102000125 | G | A | missense | 0,000003980 | 17/78 | 1314 | G/D | healthy | tail domain | 23,50 | 5,56 | 0,27 | 1,00 | 0,19 | 0,41 | 0,00 | 0,79 | 0,77 | 1,03 | 98,84 |
| 14 | 102000136 | C | T | missense | 0,000003980 | 17/78 | 1318 | R/C | healthy | tail domain | 27,20 | 5,56 | 0,64 | 1,00 | 0,50 | 0,01 | 0,75 | 0,67 | 0,76 | 0,88 | 94,34 |
| 14 | 102000137 | G | A | missense | 0,000011900 | 17/78 | 1318 | R/H | healthy | tail domain | 28,10 | 5,56 | 0,00 | 1,00 | 0,49 | 0,02 | 0,58 | 0,67 | 0,76 | 0,88 | 94,34 |
| 14 | 102000139 | G | A | missense | 0,000007960 | 17/78 | 1319 | V/M | healthy | tail domain | 24,30 | 5,56 | 0,00 | 1,00 | 0,24 | 0,14 | 0,76 | 0,67 | 0,76 | 0,88 | 94,34 |
| 14 | 102000143 | A | C | missense | 0,000003980 | 17/78 | 1320 | Q/P | healthy | tail domain | 23,90 | 5,56 | 0,55 | 1,00 | 0,37 | 0,30 | 0,01 | 0,67 | 0,76 | 0,88 | 94,23 |
| 14 | 102000305 | A | C | missense | 0,000000000 | 18/78 | 1327 | Q/P | healthy | tail domain | 23,20 | 5,76 | 0,33 | 1,00 | 0,15 | 0,47 | 0,01 | 0,64 | 0,78 | 0,81 | 89,33 |
| 14 | 102000305 | A | G | missense | 0,000011900 | 18/78 | 1327 | Q/R | patient | tail domain | 25,60 | 5,76 | 0,54 | 1,00 | 0,45 | 0,02 | 0,32 | 0,64 | 0,78 | 0,81 | 89,33 |
| 14 | 102000331 | C | T | missense | 0,000000000 | 18/78 | 1336 | L/F | study patient | tail domain | 26,30 | 5,86 | 0,55 | 1,00 | 0,44 | 0,00 | 1,00 | 0,50 | 0,79 | 0,64 | 59,50 |
| 14 | 102000340 | G | T | missense | 0,000003980 | 18/78 | 1339 | V/F | healthy | tail domain | 25,70 | 4,96 | 0,74 | 1,00 | 0,29 | 0,03 | 0,58 | 0,63 | 0,78 | 0,80 | 88,16 |
| 14 | 102000357 | T | A | missense | 0,000011900 | 18/78 | 1344 | D/E | healthy | tail domain | 15,93 | -5,22 | 0,44 | 1,00 | 0,21 | 0,15 | 0,15 | 0,57 | 0,79 | 0,73 | 77,21 |
| 14 | 102000367 | G | A | missense | 0,000000000 | 18/78 | 1348 | E/K | patient | tail domain | 31,00 | 5,64 | 0,42 | 1,00 | 0,39 | 0,00 | 0,62 | NA | NA |  | NA |
| 14 | 102000373 | C | T | missense | 0,000003980 | 18/78 | 1350 | P/S | healthy | tail domain | 23,00 | 5,54 | 0,52 | 1,00 | 0,33 | 0,70 | 0,62 | 0,63 | 0,77 | 0,81 | 89,33 |
| 14 | 102000379 | G | A | missense | 0,000003980 | 18/78 | 1352 | V/I | healthy | tail domain | 22,80 | 5,64 | 0,37 | 1,00 | 0,13 | 0,26 | 0,17 | 0,67 | 0,78 | 0,86 | 93,05 |
| 14 | 102000972 | G | T | missense | 0,000003980 | 19/78 | 1365 | A/S | healthy | tail domain | 23,80 | 5,85 | 0,28 | 1,00 | 0,17 | 0,29 | 0,01 | 0,50 | 0,76 | 0,66 | 65,07 |
| 14 | 102000976 | T | C | missense | 0,000003980 | 19/78 | 1366 | L/P | healthy | tail domain | 32,00 | 5,85 | 0,84 | 1,00 | 0,79 | 0,00 | 0,97 | 0,42 | 0,75 | 0,55 | 44,07 |
| 14 | 102001006 | G | A | missense | 0,000015900 | 19/78 | 1376 | R/Q | healthy | linker domain | 27,30 | 5,85 | 0,48 | 1,00 | 0,41 | 0,04 | 0,95 | 0,27 | 0,75 | 0,36 | 14,81 |
| 14 | 102001018 | A | G | missense | 0,000003980 | 19/78 | 1380 | Y/C | healthy | linker domain | 28,50 | 5,85 | 0,75 | 1,00 | 0,75 | 0,00 | 1,00 | 0,27 | 0,76 | 0,35 | 14,20 |
| 14 | 102001153 | G | T | missense | 0,000003980 | 20/78 | 1398 | M/I | healthy | linker domain | 23,50 | 5,85 | 0,00 | 1,00 | 0,25 | 0,21 | 0,11 | 0,13 | 0,79 | 0,16 | 1,74 |
| 14 | 102001194 | A | G | missense | 0,000000000 | 20/78 | 1412 | H/R | patient | linker domain | 27,10 | 5,85 | 0,84 | 1,00 | 0,89 | 0,00 | 1,00 | 0,50 | 0,78 | 0,64 | 60,94 |
| 14 | 102001223 | G | A | missense | 0,000007950 | 20/78 | 1422 | V/I | healthy | linker domain | 24,90 | 5,85 | 0,61 | 1,00 | 0,30 | 0,21 | 0,85 | 0,58 | 0,79 | 0,74 | 79,45 |
| 14 | 102001226 | A | G | missense | 0,000003980 | 20/78 | 1423 | N/D | healthy | linker domain | 23,10 | 5,85 | 0,59 | 1,00 | 0,19 | 0,19 | 0,06 | 0,64 | 0,79 | 0,81 | 88,60 |
| 14 | 102001242 | A | T | missense | 0,000003980 | 20/78 | 1428 | E/V | healthy | linker domain | 30,00 | 5,85 | 0,74 | 1,00 | 0,64 | 0,04 | 0,80 | 0,58 | 0,80 | 0,73 | 78,50 |
| 14 | 102001248 | C | A | missense | 0,000003980 | 20/78 | 1430 | T/N | healthy | linker domain | 25,20 | 5,85 | 0,75 | 1,00 | 0,52 | 0,01 | 0,75 | 0,60 | 0,79 | 0,76 | 82,72 |
| 14 | 102001253 | G | A | missense | 0,000003980 | 20/78 | 1432 | G/S | healthy | linker domain | 31,00 | 5,85 | 0,58 | 1,00 | 0,58 | 0,02 | 1,00 | 0,59 | 0,78 | 0,75 | 81,13 |
| 14 | 102001259 | A | C | missense | 0,000043700 | 20/78 | 1434 | I/L | healthy | linker domain | 22,40 | 5,85 | 0,62 | 1,00 | 0,18 | 0,23 | 0,02 | 0,61 | 0,77 | 0,79 | 86,23 |
| 14 | 102001269 | T | C | missense | 0,000003980 | 20/78 | 1437 | V/A | healthy | linker domain | 23,10 | 5,85 | 0,56 | 1,00 | 0,15 | 0,29 | 0,06 | 0,65 | 0,78 | 0,83 | 91,13 |
| 14 | 102001290 | C | T | missense | 0,000019900 | 20/78 | 1444 | A/V | healthy | linker domain | 21,40 | 5,85 | 0,00 | 1,00 | 0,10 | 0,41 | 0,01 | 0,47 | 0,78 | 0,61 | 54,53 |
| 14 | 102001292 | A | G | missense | 0,000003980 | 20/78 | 1445 | I/V | healthy | linker domain | 1,44 | -6,88 | 0,49 | 1,00 | 0,17 | 0,84 | 0,01 | 0,47 | 0,77 | 0,61 | 54,70 |
| 14 | 102001295 | G | A | missense | 0,000007950 | 20/78 | 1446 | V/I | healthy | linker domain | 21,70 | 5,85 | 0,00 | 1,00 | 0,25 | 1,00 | 0,06 | 0,47 | 0,78 | 0,60 | 53,60 |
| 14 | 102001304 | G | A | missense | 0,000007950 | 20/78 | 1449 | V/I | healthy | linker domain | 22,50 | 5,85 | 0,52 | 1,00 | 0,23 | 0,73 | 0,08 | 0,50 | 0,78 | 0,64 | 61,24 |
| 14 | 102001593 | G | A | missense | 0,000003980 | 21/78 | 1485 | R/H | healthy | linker domain | 26,30 | 5,88 | 0,58 | 1,00 | 0,38 | 0,02 | 0,15 | 0,33 | 0,81 | 0,41 | 21,54 |
| 14 | 102001600 | C | G | missense | 0,000003980 | 21/78 | 1487 | I/M | healthy | linker domain | 23,30 | 1,30 | 0,65 | 1,00 | 0,59 | 0,01 | 1,00 | NA | NA |  | NA |
| 14 | 102001623 | A | G | missense | 0,000035800 | 21/78 | 1495 | N/S | healthy | linker domain | 22,10 | 5,88 | 0,46 | 1,00 | 0,16 | 0,66 | 0,10 | 0,64 | 0,79 | 0,80 | 87,86 |
| 14 | 102001640 | A | C | missense | 0,000000000 | 21/78 | 1501 | I/L | healthy | linker domain | 21,50 | 5,88 | 0,00 | 1,00 | 0,16 | 1,00 | 0,10 | 0,50 | 0,80 | 0,63 | 57,22 |
| 14 | 102001644 | A | G | missense | 0,000003980 | 21/78 | 1502 | N/S | healthy | linker domain | 26,30 | 5,88 | 0,43 | 1,00 | 0,44 | 0,04 | 0,85 | 0,50 | 0,80 | 0,62 | 56,94 |
| 14 | 102001649 | G | A | missense | 0,000051700 | 21/78 | 1504 | V/I | healthy | linker domain | 23,30 | 5,88 | 0,52 | 1,00 | 0,20 | 0,10 | 0,02 | 0,46 | 0,80 | 0,57 | 46,65 |
| 14 | 102001653 | C | T | missense | 0,000023900 | 21/78 | 1505 | S/L | healthy | linker domain | 23,10 | 5,88 | 0,68 | 1,00 | 0,38 | 0,17 | 0,24 | 0,46 | 0,80 | 0,57 | 46,65 |
| 14 | 102002546 | G | A | missense | 0,000000000 | 22/78 | 1518 | E/K | patient | linker domain | 34,00 | 5,70 | 0,49 | 1,00 | 0,57 | 0,00 | 1,00 | NA | NA |  | NA |
| 14 | 102002591 | C | G | missense | 0,000019900 | 22/78 | 1533 | L/V | healthy | linker domain | 25,90 | 5,70 | 0,59 | 1,00 | 0,24 | 0,00 | 0,75 | 0,29 | 0,79 | 0,36 | 15,47 |
| 14 | 102002603 | T | C | missense | 0,000000000 | 22/78 | 1537 | W/R | study patient | linker domain | 33,00 | 5,70 | 0,87 | 1,00 | 0,83 | 0,00 | 1,00 | 0,38 | 0,79 | 0,47 | 30,17 |
| 14 | 102002621 | C | T | missense | 0,000003980 | 22/78 | 1543 | R/W | healthy | linker domain | 31,00 | 5,76 | 0,00 | 1,00 | 0,63 | 0,00 | 1,00 | NA | NA |  | NA |
| 14 | 102002645 | T | C | missense | 0,000027800 | 22/78 | 1551 | F/L | healthy | linker domain | 32,00 | 5,76 | 0,89 | 1,00 | 0,90 | 0,00 | 1,00 | 0,23 | 0,77 | 0,30 | 10,24 |
| 14 | 102002651 | G | A | missense | 0,000007950 | 22/78 | 1553 | G/S | healthy | linker domain | 32,00 | 5,76 | 0,71 | 1,00 | 0,52 | 0,00 | 0,98 | 0,21 | 0,77 | 0,28 | 8,72 |
| 14 | 102002694 | G | A | missense | 0,000000000 | 22/78 | 1567 | R/L | patient | linker domain | 34,00 | 4,88 | 0,70 | 1,00 | 0,51 | 0,00 | 0,99 | 0,20 | 0,76 | 0,26 | 7,32 |
| 14 | 102002694 | G | T | missense | 0,000000000 | 22/78 | 1567 | R/Q | patient | linker domain | 34,00 | 4,88 | 0,76 | 1,00 | 0,59 | 0,00 | 1,00 | 0,20 | 0,76 | 0,26 | 7,32 |
| 14 | 102002793 | A | G | missense | 0,000003980 | 23/78 | 1571 | I/V | healthy | linker domain | 25,70 | 5,52 | 0,66 | 1,00 | 0,20 | 0,11 | 0,81 | 0,15 | 0,77 | 0,20 | 3,40 |
| 14 | 102002811 | G | A | missense | 0,000015900 | 23/78 | 1577 | A/T | healthy | linker domain | 23,60 | 5,43 | 0,33 | 1,00 | 0,14 | 0,43 | 0,04 | 0,44 | 0,76 | 0,59 | 50,38 |
| 14 | 102002823 | A | G | missense | 0,000000000 | 23/78 | 1581 | K/E | patient | linker domain | 29,50 | 5,43 | 0,51 | 1,00 | 0,57 | 0,00 | 0,99 | 0,44 | 0,75 | 0,59 | 51,24 |
| 14 | 102002847 | A | G | missense | 0,000003980 | 23/78 | 1589 | M/V | healthy | linker domain | 22,80 | 5,43 | 0,00 | 1,00 | 0,29 | 0,06 | 0,01 | 0,50 | 0,75 | 0,66 | 66,02 |
| 14 | 102002848 | T | C | missense | 0,000003980 | 23/78 | 1589 | M/T | healthy | linker domain | 23,10 | 5,43 | 0,71 | 1,00 | 0,47 | 0,06 | 0,03 | 0,50 | 0,75 | 0,66 | 66,02 |
| 14 | 102002881 | C | G | missense | 0,000003980 | 23/78 | 1600 | S/C | healthy | linker domain | 27,20 | 5,43 | 0,00 | 1,00 | 0,48 | 0,00 | 0,94 | 0,38 | 0,75 | 0,50 | 35,21 |
| 14 | 102002933 | A | C | missense | 0,000007960 | 23/78 | 1617 | E/D | healthy | linker domain | 22,80 | -0,50 | 0,42 | 1,00 | 0,28 | 0,05 | 0,87 | 0,10 | 0,75 | 0,13 | 0,95 |
| 14 | 102002950 | G | A | missense | 0,000000000 | 23/78 | 1623 | R/Q | patient | linker domain | 34,00 | 5,43 | 0,81 | 1,00 | 0,88 | 0,00 | 1,00 | 0,08 | 0,76 | 0,11 | 0,67 |
| 14 | 102004555 | A | T | missense | 0,000003990 | 24/78 | 1641 | I/F | healthy | linker domain | 28,50 | 5,73 | 0,71 | 1,00 | 0,75 | 0,00 | 0,98 | 0,22 | 0,79 | 0,28 | 8,91 |
| 14 | 102004576 | G | A | missense | 0,000023900 | 24/78 | 1648 | A/T | healthy | linker domain | 22,70 | 5,73 | 0,36 | 1,00 | 0,26 | 0,63 | 0,01 | 0,43 | 0,80 | 0,54 | 40,48 |
| 14 | 102004604 | T | C | missense | 0,000003980 | 24/78 | 1657 | M/T | healthy | linker domain | 27,30 | 5,73 | 0,84 | 1,00 | 0,77 | 0,00 | 1,00 | 0,67 | 0,78 | 0,85 | 92,55 |
| 14 | 102004610 | C | T | missense | 0,000003980 | 24/78 | 1659 | A/V | healthy | linker domain | 27,20 | 4,94 | 0,57 | 1,00 | 0,52 | 0,00 | 1,00 | 0,67 | 0,78 | 0,86 | 92,94 |
| 14 | 102004613 | G | C | missense | 0,000003980 | 24/78 | 1660 | G/A | healthy | linker domain | 28,60 | 5,83 | 0,79 | 1,00 | 0,70 | 0,00 | 1,00 | 0,67 | 0,78 | 0,86 | 92,94 |
| 14 | 102004619 | C | T | missense | 0,000003980 | 24/78 | 1662 | S/L | healthy | linker domain | 24,50 | 5,83 | 0,58 | 1,00 | 0,41 | 0,00 | 0,27 | 0,63 | 0,78 | 0,82 | 89,67 |
| 14 | 102004635 | C | A | missense | 0,000007950 | 24/78 | 1667 | N/K | healthy | linker domain | 15,73 | -7,57 | 0,59 | 1,00 | 0,33 | 0,01 | 0,59 | 0,72 | 0,78 | 0,93 | 96,47 |
| 14 | 102004636 | G | A | missense | 0,000003980 | 24/78 | 1668 | E/K | healthy | linker domain | 25,90 | 5,83 | 0,61 | 1,00 | 0,31 | 0,03 | 0,18 | 0,72 | 0,78 | 0,93 | 96,47 |
| 14 | 102004636 | G | C | missense | 0,000007950 | 24/78 | 1668 | E/Q | healthy | linker domain | 28,00 | 5,83 | 0,56 | 1,00 | 0,36 | 0,02 | 0,74 | 0,72 | 0,78 | 0,93 | 96,47 |
| 14 | 102004639 | G | T | missense | 0,000011900 | 24/78 | 1669 | D/Y | healthy | linker domain | 29,20 | 5,83 | 0,69 | 1,00 | 0,59 | 0,00 | 0,97 | 0,78 | 0,78 | 1,00 | 98,17 |
| 14 | 102004643 | A | G | missense | 0,000007950 | 24/78 | 1670 | N/S | healthy | linker domain | 16,96 | 2,15 | 0,00 | 1,00 | 0,08 | 0,73 | 0,01 | 0,80 | 0,77 | 1,04 | 99,10 |
| 14 | 102004645 | T | G | missense | 0,000003980 | 24/78 | 1671 | S/A | healthy | linker domain | 21,80 | 5,83 | 0,51 | 1,00 | 0,07 | 0,13 | 0,00 | 0,80 | 0,77 | 1,04 | 99,16 |
| 14 | 102004646 | C | G | missense | 0,000007950 | 24/78 | 1671 | S/C | healthy | linker domain | 24,20 | 5,83 | 0,60 | 1,00 | 0,24 | 0,00 | 0,42 | 0,80 | 0,77 | 1,04 | 99,16 |
| 14 | 102004669 | C | T | missense | 0,000003980 | 24/78 | 1679 | R/W | healthy | linker domain | 33,00 | 5,53 | 0,63 | 1,00 | 0,65 | 0,00 | 0,99 | NA | NA |  | NA |
| 14 | 102004672 | G | C | missense | 0,000003980 | 24/78 | 1680 | E/Q | healthy | linker domain | 31,00 | 5,53 | 0,63 | 1,00 | 0,52 | 0,00 | 1,00 | 0,79 | 0,76 | 1,04 | 99,25 |
| 14 | 102004762 | G | A | missense | 0,000015900 | 25/78 | 1684 | V/I | healthy | linker domain | 24,50 | 5,83 | 0,63 | 1,00 | 0,25 | 0,05 | 0,26 | 0,80 | 0,76 | 1,05 | 99,29 |
| 14 | 102004767 | G | C | missense | 0,000003980 | 25/78 | 1685 | M/I | healthy | linker domain | 20,10 | 4,95 | 0,56 | 1,00 | 0,06 | 0,52 | 0,00 | 0,79 | 0,76 | 1,04 | 99,14 |
| 14 | 102004766 | T | G | missense | 0,000003980 | 25/78 | 1685 | M/R | healthy | linker domain | 21,90 | 5,83 | 0,48 | 0,57 | 0,17 | 0,79 | 0,00 | 0,79 | 0,76 | 1,04 | 99,14 |
| 14 | 102004774 | A | G | missense | 0,000003980 | 25/78 | 1688 | T/A | healthy | linker domain | 23,40 | 4,67 | 0,46 | 1,00 | 0,29 | 0,35 | 0,25 | 0,77 | 0,77 | 1,00 | 98,28 |
| 14 | 102004786 | A | G | missense | 0,000015900 | 25/78 | 1692 | I/V | healthy | linker domain | 22,60 | 5,83 | 0,59 | 1,00 | 0,22 | 0,25 | 0,07 | 0,69 | 0,76 | 0,90 | 95,29 |
| 14 | 102004795 | C | T | missense | 0,000003980 | 25/78 | 1695 | H/Y | healthy | linker domain | 19,71 | 5,85 | 0,45 | 1,00 | 0,22 | 1,00 | 0,01 | 0,71 | 0,76 | 0,94 | 96,79 |
| 14 | 102004815 | G | T | missense | 0,000003980 | 25/78 | 1701 | W/C | healthy | linker domain | 34,00 | 5,85 | 0,87 | 1,00 | 0,82 | 0,00 | 1,00 | NA | NA |  | NA |
| 14 | 102004816 | C | T | missense | 0,000003980 | 25/78 | 1702 | L/F | healthy | linker domain | 26,10 | 5,85 | 0,76 | 1,00 | 0,78 | 0,00 | 1,00 | 0,58 | 0,76 | 0,77 | 83,39 |
| 14 | 102004833 | G | C | missense | 0,000023900 | 25/78 | 1707 | K/N | healthy | linker domain | 20,30 | 4,78 | 0,45 | 1,00 | 0,05 | 0,64 | 0,01 | NA | NA |  | NA |
| 14 | 102004861 | C | G | missense | 0,000003980 | 25/78 | 1717 | L/V | healthy | linker domain | 23,30 | 5,85 | 0,00 | 1,00 | 0,32 | 0,04 | 0,81 | 0,23 | 0,76 | 0,31 | 10,98 |
| 14 | 102004909 | A | G | missense | 0,000019900 | 25/78 | 1733 | I/V | healthy | linker domain | 21,20 | 5,85 | 0,00 | 1,00 | 0,05 | 0,44 | 0,00 | 0,33 | 0,75 | 0,45 | 27,16 |
| 14 | 102004922 | C | A | missense | 0,000007950 | 25/78 | 1737 | T/N | healthy | linker domain | 21,30 | 5,79 | 0,31 | 0,99 | 0,01 | 0,17 | 0,01 | 0,43 | 0,75 | 0,57 | 46,98 |
| 14 | 102004925 | A | T | missense | 0,000003980 | 25/78 | 1738 | Y/F | healthy | linker domain | 22,10 | 5,85 | 0,48 | 1,00 | 0,17 | 0,99 | 0,05 | 0,50 | 0,76 | 0,66 | 64,73 |
| 14 | 102005043 | C | G | missense | 0,000007960 | 26/78 | 1747 | A/G | healthy | linker domain | 26,40 | 5,85 | 0,45 | 1,00 | 0,15 | 0,01 | 0,06 | 0,69 | 0,76 | 0,91 | 95,50 |
| 14 | 102005051 | G | C | missense | 0,000003980 | 26/78 | 1750 | V/L | healthy | linker domain | 25,50 | 5,85 | 0,45 | 1,00 | 0,31 | 0,02 | 0,33 | 0,65 | 0,77 | 0,85 | 92,00 |
| 14 | 102005051 | G | A | missense | 0,000059700 | 26/78 | 1750 | V/M | healthy | linker domain | 31,00 | 5,85 | 0,00 | 1,00 | 0,47 | 0,04 | 0,92 | 0,65 | 0,77 | 0,85 | 92,00 |
| 14 | 102005064 | C | A | missense | 0,000003980 | 26/78 | 1754 | A/D | healthy | linker domain | 26,60 | 5,85 | 0,53 | 1,00 | 0,49 | 0,01 | 0,61 | 0,60 | 0,77 | 0,78 | 85,22 |
| 14 | 102005087 | G | A | missense | 0,000003980 | 26/78 | 1762 | V/M | healthy | linker domain | 23,30 | 5,75 | 0,00 | 1,00 | 0,20 | 0,07 | 0,05 | 0,61 | 0,75 | 0,82 | 90,23 |
| 14 | 102005088 | T | C | missense | 0,000007960 | 26/78 | 1762 | V/A | healthy | linker domain | 24,50 | 5,75 | 0,73 | 1,00 | 0,27 | 0,03 | 0,01 | 0,61 | 0,75 | 0,82 | 90,23 |
| 14 | 102005097 | C | T | missense | 0,000003980 | 26/78 | 1765 | A/V | healthy | linker domain | 22,50 | 5,75 | 0,00 | 1,00 | 0,25 | 0,09 | 0,04 | 0,58 | 0,75 | 0,78 | 84,92 |
| 14 | 102005096 | G | T | missense | 0,000023900 | 26/78 | 1765 | A/S | healthy | linker domain | 23,00 | 5,75 | 0,38 | 1,00 | 0,22 | 0,29 | 0,34 | 0,58 | 0,75 | 0,78 | 84,92 |
| 14 | 102005096 | G | A | missense | 0,000000000 | 26/78 | 1765 | A/T | healthy | linker domain | 23,40 | 5,75 | 0,00 | 1,00 | 0,37 | 0,06 | 0,10 | 0,58 | 0,75 | 0,78 | 84,92 |
| 14 | 102005108 | A | C | missense | 0,000003980 | 26/78 | 1769 | M/L | healthy | linker domain | 9,55 | -5,13 | 0,34 | 1,00 | 0,05 | 0,32 | 0,00 | 0,55 | 0,74 | 0,74 | 79,97 |
| 14 | 102005108 | A | G | missense | 0,000003980 | 26/78 | 1769 | M/V | healthy | linker domain | 11,49 | -5,13 | 0,41 | 1,00 | 0,06 | 0,31 | 0,00 | 0,55 | 0,74 | 0,74 | 79,97 |
| 14 | 102005109 | T | C | missense | 0,000011900 | 26/78 | 1769 | M/T | healthy | linker domain | 15,66 | 4,61 | 0,39 | 1,00 | 0,05 | 0,57 | 0,00 | 0,55 | 0,74 | 0,74 | 79,97 |
| 14 | 102005114 | G | A | missense | 0,000043800 | 26/78 | 1771 | G/R | healthy | linker domain | 23,40 | 5,75 | 0,00 | 1,00 | 0,14 | 0,02 | 0,03 | 0,52 | 0,74 | 0,70 | 71,57 |
| 14 | 102005118 | G | A | missense | 0,000003980 | 26/78 | 1772 | G/D | healthy | linker domain | 17,24 | 4,87 | 0,26 | 1,00 | 0,09 | 0,30 | 0,00 | 0,52 | 0,74 | 0,69 | 71,32 |
| 14 | 102005117 | G | A | missense | 0,000011900 | 26/78 | 1772 | G/S | healthy | linker domain | 17,90 | 4,81 | 0,24 | 1,00 | 0,03 | 0,29 | 0,00 | 0,52 | 0,74 | 0,69 | 71,32 |
| 14 | 102005120 | G | A | missense | 0,000019900 | 26/78 | 1773 | G/R | healthy | linker domain | 22,70 | 5,75 | 0,35 | 1,00 | 0,21 | 0,07 | 0,05 | 0,50 | 0,74 | 0,67 | 68,20 |
| 14 | 102005130 | C | T | missense | 0,000015900 | 26/78 | 1776 | A/V | healthy | linker domain | 12,12 | 4,85 | 0,00 | 1,00 | 0,03 | 0,18 | 0,01 | 0,53 | 0,74 | 0,71 | 74,41 |
| 14 | 102005129 | G | A | missense | 0,000091500 | 26/78 | 1776 | A/T | healthy | linker domain | 13,25 | 5,75 | 0,00 | 1,00 | 0,06 | 0,47 | 0,00 | 0,53 | 0,74 | 0,71 | 74,41 |
| 14 | 102005133 | C | A | missense | 0,000011900 | 26/78 | 1777 | P/H | healthy | linker domain | 17,56 | 5,75 | 0,34 | 1,00 | 0,14 | 0,09 | 0,00 | 0,55 | 0,74 | 0,74 | 78,91 |
| 14 | 102005150 | A | G | missense | 0,000003980 | 26/78 | 1783 | S/G | healthy | linker domain | 21,50 | 5,75 | 0,36 | 0,57 | 0,04 | 0,38 | 0,00 | 0,57 | 0,74 | 0,76 | 82,72 |
| 14 | 102005168 | C | G | missense | 0,000003980 | 26/78 | 1789 | L/V | healthy | linker domain | 24,80 | 4,68 | 0,40 | 1,00 | 0,36 | 0,00 | 0,62 | 0,44 | 0,73 | 0,61 | 54,53 |
| 14 | 102005172 | A | G | missense | 0,000003980 | 26/78 | 1790 | N/S | healthy | linker domain | 21,80 | 4,58 | 0,00 | 1,00 | 0,06 | 0,39 | 0,01 | 0,44 | 0,73 | 0,61 | 54,81 |
| 14 | 102005208 | C | T | missense | 0,000003980 | 26/78 | 1802 | P/L | healthy | linker domain | 23,30 | 5,75 | 0,37 | 1,00 | 0,26 | 0,10 | 0,02 | 0,54 | 0,75 | 0,72 | 76,26 |
| 14 | 102005214 | G | A | missense | 0,000007960 | 26/78 | 1804 | R/Q | healthy | linker domain | 25,80 | 4,82 | 0,37 | 1,00 | 0,32 | 0,35 | 0,92 | 0,54 | 0,75 | 0,72 | 76,26 |
| 14 | 102005225 | C | A | missense | 0,000003980 | 26/78 | 1808 | L/I | healthy | linker domain | 21,10 | 4,72 | 0,39 | 1,00 | 0,12 | 0,23 | 0,06 | 0,58 | 0,75 | 0,78 | 84,70 |
| 14 | 102005226 | T | A | missense | 0,000003980 | 26/78 | 1808 | L/Q | healthy | linker domain | 31,00 | 5,71 | 0,76 | 1,00 | 0,67 | 0,02 | 0,91 | 0,58 | 0,75 | 0,78 | 84,70 |
| 14 | 102005896 | G | C | missense | 0,000003980 | 27/78 | 1814 | E/D | healthy | linker domain | 24,00 | 3,30 | 0,38 | 1,00 | 0,27 | 0,04 | 0,84 | NA | NA |  | NA |
| 14 | 102005913 | A | G | missense | 0,000003980 | 27/78 | 1820 | D/G | healthy | linker domain | 25,30 | 6,07 | 0,64 | 1,00 | 0,54 | 0,01 | 0,11 | 0,70 | 0,77 | 0,91 | 95,42 |
| 14 | 102005918 | A | T | missense | 0,000003980 | 27/78 | 1822 | T/S | healthy | linker domain | 22,60 | 6,07 | 0,51 | 1,00 | 0,28 | 0,07 | 0,10 | 0,70 | 0,78 | 0,90 | 94,90 |
| 14 | 102005932 | C | G | missense | 0,000003980 | 27/78 | 1826 | I/M | healthy | linker domain | 17,71 | 0,88 | 0,45 | 1,00 | 0,04 | 0,23 | 0,03 | NA | NA |  | NA |
| 14 | 102005941 | G | C | missense | 0,000003980 | 27/78 | 1829 | K/N | healthy | linker domain | 20,50 | 3,28 | 0,43 | 1,00 | 0,06 | 0,63 | 0,01 | 0,74 | 0,81 | 0,91 | 95,72 |
| 14 | 102005944 | T | G | missense | 0,000003980 | 27/78 | 1830 | I/M | healthy | linker domain | 14,59 | -6,37 | 0,41 | 1,00 | 0,21 | 0,01 | 0,37 | NA | NA |  | NA |
| 14 | 102005942 | A | G | missense | 0,000003980 | 27/78 | 1830 | I/V | healthy | linker domain | 15,39 | 2,46 | 0,00 | 1,00 | 0,02 | 1,00 | 0,00 | 0,68 | 0,81 | 0,85 | 92,23 |
| 14 | 102005943 | T | C | missense | 0,000003980 | 27/78 | 1830 | I/T | healthy | linker domain | 23,40 | 6,07 | 0,47 | 1,00 | 0,20 | 0,09 | 0,00 | 0,68 | 0,81 | 0,85 | 92,23 |
| 14 | 102005946 | A | T | missense | 0,000007950 | 27/78 | 1831 | D/V | healthy | linker domain | 23,50 | 6,07 | 0,46 | 1,00 | 0,25 | 0,26 | 0,00 | 0,68 | 0,80 | 0,86 | 92,73 |
| 14 | 102005948 | A | G | missense | 0,000003980 | 27/78 | 1832 | N/D | healthy | linker domain | 23,30 | 6,07 | 0,35 | 1,00 | 0,09 | 0,17 | 0,00 | 0,68 | 0,80 | 0,85 | 92,43 |
| 14 | 102005951 | G | A | missense | 0,000023900 | 27/78 | 1833 | A/T | healthy | linker domain | 20,90 | 2,76 | 0,00 | 0,99 | 0,03 | 0,19 | 0,02 | 0,68 | 0,80 | 0,85 | 92,64 |
| 14 | 102005955 | A | G | missense | 0,000003980 | 27/78 | 1834 | K/R | healthy | linker domain | 23,30 | 6,07 | 0,00 | 1,00 | 0,17 | 0,28 | 0,00 | 0,68 | 0,80 | 0,85 | 92,47 |
| 14 | 102005970 | T | C | missense | 0,000003980 | 27/78 | 1839 | L/P | healthy | linker domain | 29,20 | 6,07 | 0,74 | 1,00 | 0,54 | 0,00 | 0,97 | 0,61 | 0,80 | 0,77 | 83,22 |
| 14 | 102005974 | C | A | missense | 0,000011900 | 27/78 | 1840 | S/R | healthy | linker domain | 18,57 | 5,17 | 0,42 | 1,00 | 0,18 | 0,89 | 0,00 | 0,67 | 0,80 | 0,83 | 91,13 |
| 14 | 102006018 | A | G | missense | 0,000003980 | 27/78 | 1855 | Q/R | healthy | linker domain | 22,70 | 6,07 | 0,35 | 1,00 | 0,10 | 0,22 | 0,00 | 0,44 | 0,80 | 0,56 | 44,61 |
| 14 | 102006034 | A | T | missense | 0,000003980 | 27/78 | 1860 | Q/H | healthy | linker domain | 12,68 | -11,50 | 0,48 | 0,95 | 0,36 | 0,67 | 0,00 | 0,43 | 0,80 | 0,54 | 41,21 |
| 14 | 102006048 | A | G | missense | 0,000011900 | 27/78 | 1865 | K/R | healthy | linker domain | 23,10 | 6,07 | 0,00 | 1,00 | 0,17 | 0,21 | 0,00 | 0,38 | 0,78 | 0,48 | 31,70 |
| 14 | 102006113 | C | T | missense | 0,000003980 | 27/78 | 1887 | R/C | healthy | motor domain | 27,40 | 6,07 | 0,50 | 1,00 | 0,54 | 0,00 | 1,00 | 0,29 | 0,75 | 0,40 | 19,09 |
| 14 | 102006113 | C | A | missense | 0,000011900 | 27/78 | 1887 | R/S | healthy | motor domain | 32,00 | 6,07 | 0,60 | 1,00 | 0,54 | 0,00 | 1,00 | 0,29 | 0,75 | 0,40 | 19,09 |
| 14 | 102006114 | G | A | missense | 0,000011900 | 27/78 | 1887 | R/H | healthy | motor domain | 33,00 | 6,07 | 0,46 | 1,00 | 0,57 | 0,00 | 1,00 | 0,29 | 0,75 | 0,40 | 19,09 |
| 14 | 102006120 | A | G | missense | 0,000003980 | 27/78 | 1889 | Y/C | healthy | motor domain | 32,00 | 6,07 | 0,54 | 1,00 | 0,56 | 0,00 | 1,00 | 0,29 | 0,73 | 0,40 | 19,78 |
| 14 | 102006144 | A | T | missense | 0,000003990 | 27/78 | 1897 | E/V | healthy | motor domain | 29,40 | 6,07 | 0,44 | 1,00 | 0,36 | 0,05 | 0,78 | 0,31 | 0,73 | 0,43 | 23,35 |
| 14 | 102007064 | C | T | missense | 0,000003980 | 28/78 | 1925 | R/W | healthy | motor domain | 32,00 | 4,88 | 0,60 | 1,00 | 0,33 | 0,00 | 1,00 | NA | NA |  | NA |
| 14 | 102007107 | A | T | missense | 0,000000000 | 28/78 | 1939 | Q/L | healthy | motor domain | 34,00 | 5,79 | 0,49 | 1,00 | 0,32 | 0,00 | 0,98 | 0,57 | 0,78 | 0,73 | 78,67 |
| 14 | 102008188 | G | A | missense | 0,000003980 | 29/78 | 1943 | R/Q | healthy | motor domain | 33,00 | 5,24 | 0,00 | 1,00 | 0,54 | 0,00 | 1,00 | 0,43 | 0,78 | 0,55 | 44,07 |
| 14 | 102008217 | G | T | missense | 0,000003980 | 29/78 | 1953 | A/S | healthy | motor domain | 29,20 | 5,32 | 0,55 | 1,00 | 0,28 | 0,00 | 1,00 | 0,30 | 0,78 | 0,39 | 17,86 |
| 14 | 102008244 | C | T | missense | 0,000000000 | 29/78 | 1962 | R/C | patient | motor domain | 28,70 | 4,32 | 0,00 | NA | NA | NA | NA | 0,33 | 0,76 | 0,44 | 25,20 |
| 14 | 102008245 | C | A | missense | 0,000000000 | 29/78 | 1962 | R/H | patient | motor domain | NA | 5,32 | 0,48 | 1,00 | 0,85 | 0,00 | 1,00 | 0,33 | 0,76 | 0,44 | 25,20 |
| 14 | 102008268 | G | T | missense | 0,000003980 | 29/78 | 1970 | A/S | healthy | motor domain | 28,70 | 5,32 | 0,47 | 1,00 | 0,32 | 0,00 | 1,00 | 0,36 | 0,78 | 0,46 | 28,51 |
| 14 | 102008283 | G | C | missense | 0,000003980 | 29/78 | 1975 | V/L | healthy | motor domain | 24,70 | 5,32 | 0,36 | 1,00 | 0,21 | 0,00 | 0,22 | 0,38 | 0,77 | 0,48 | 32,02 |
| 14 | 102008287 | A | G | missense | 0,000007950 | 29/78 | 1976 | Q/R | healthy | motor domain | 29,20 | 5,32 | 0,33 | 1,00 | 0,43 | 0,00 | 0,98 | 0,42 | 0,77 | 0,54 | 42,16 |
| 14 | 102008308 | G | A | missense | 0,000003980 | 29/78 | 1983 | R/H | healthy | motor domain | 33,00 | 5,32 | 0,00 | 1,00 | 0,25 | 0,00 | 0,87 | 0,46 | 0,75 | 0,60 | 53,73 |
| 14 | 102008313 | C | G | missense | 0,000003980 | 29/78 | 1985 | H/D | healthy | motor domain | 22,60 | 5,32 | 0,35 | 1,00 | 0,31 | 0,60 | 0,00 | 0,55 | 0,76 | 0,72 | 76,50 |
| 14 | 102008323 | C | T | missense | 0,000003980 | 29/78 | 1988 | P/L | healthy | motor domain | 23,80 | 5,32 | 0,36 | 1,00 | 0,24 | 0,10 | 0,26 | 0,53 | 0,76 | 0,70 | 71,72 |
| 14 | 102008332 | A | G | missense | 0,000003980 | 29/78 | 1991 | D/G | healthy | motor domain | 23,60 | 5,32 | 0,37 | 1,00 | 0,12 | 0,16 | 0,34 | 0,50 | 0,77 | 0,65 | 63,35 |
| 14 | 102008331 | G | A | missense | 0,000000000 | 29/78 | 1991 | D/N | healthy | motor domain | 25,90 | 5,32 | 0,00 | 1,00 | 0,11 | 0,04 | 0,29 | 0,50 | 0,77 | 0,65 | 63,35 |
| 14 | 102009843 | C | T | missense | 0,000003980 | 30/78 | 1993 | T/I | healthy | motor domain | 20,40 | 5,33 | 0,43 | 1,00 | 0,04 | 0,12 | 0,03 | 0,50 | 0,78 | 0,64 | 61,67 |
| 14 | 102009852 | C | G | missense | 0,000007950 | 30/78 | 1996 | P/R | healthy | motor domain | 23,10 | 5,33 | 0,49 | 1,00 | 0,21 | 0,26 | 0,18 | 0,53 | 0,78 | 0,68 | 68,43 |
| 14 | 102009861 | G | A | missense | 0,000003980 | 30/78 | 1999 | C/Y | healthy | motor domain | 25,40 | 5,33 | 0,42 | 1,00 | 0,32 | 0,00 | 0,00 | 0,50 | 0,78 | 0,64 | 61,09 |
| 14 | 102009894 | C | T | missense | 0,000015900 | 30/78 | 2010 | P/L | healthy | motor domain | 23,20 | 5,33 | 0,71 | 1,00 | 0,29 | 0,08 | 0,25 | 0,36 | 0,76 | 0,47 | 29,25 |
| 14 | 102009896 | G | C | missense | 0,000003980 | 30/78 | 2011 | D/H | healthy | motor domain | 29,70 | 5,33 | 0,50 | 1,00 | 0,33 | 0,05 | 0,86 | 0,39 | 0,77 | 0,50 | 35,21 |
| 14 | 102009933 | C | T | missense | 0,000003980 | 30/78 | 2023 | A/V | healthy | motor domain | 27,30 | 5,33 | 0,34 | 1,00 | 0,49 | 0,00 | 0,98 | 0,36 | 0,78 | 0,47 | 29,01 |
| 14 | 102009978 | G | A | missense | 0,000003980 | 30/78 | 2038 | S/N | healthy | motor domain | 23,60 | 5,55 | 0,43 | 1,00 | 0,22 | 0,07 | 0,08 | 0,39 | 0,76 | 0,51 | 36,13 |
| 14 | 102009987 | T | C | missense | 0,000000000 | 30/78 | 2041 | M/T | patient | motor domain | 28,20 | 5,55 | 0,71 | 1,00 | 0,75 | 0,00 | 1,00 | 0,33 | 0,75 | 0,44 | 26,68 |
| 14 | 102009998 | G | A | missense | 0,000003980 | 30/78 | 2045 | D/N | healthy | motor domain | 31,00 | 5,55 | 0,58 | 1,00 | 0,34 | 0,02 | 0,60 | 0,31 | 0,75 | 0,41 | 21,09 |
| 14 | 102010006 | G | C | missense | 0,000003980 | 30/78 | 2047 | Q/H | healthy | motor domain | 26,50 | 3,69 | 0,39 | 1,00 | 0,28 | 0,00 | 0,99 | 0,33 | 0,76 | 0,44 | 25,20 |
| 14 | 102010014 | C | G | missense | 0,000003980 | 30/78 | 2050 | A/G | healthy | motor domain | 27,60 | 5,85 | 0,51 | 1,00 | 0,35 | 0,00 | 0,96 | 0,33 | 0,76 | 0,44 | 24,90 |
| 14 | 102010046 | A | G | missense | 0,000003980 | 30/78 | 2061 | T/A | healthy | motor domain | 27,40 | 5,85 | 0,36 | 1,00 | 0,32 | 0,02 | 0,76 | 0,35 | 0,77 | 0,46 | 28,15 |
| 14 | 102010066 | C | A | missense | 0,000008000 | 30/78 | 2067 | N/K | healthy | motor domain | 16,81 | 5,85 | 0,45 | 1,00 | 0,13 | 0,84 | 0,00 | 0,33 | 0,78 | 0,43 | 23,78 |
| 14 | 102010073 | G | A | missense | 0,000000000 | 30/78 | 2070 | V/I | healthy | motor domain | 27,40 | 5,85 | 0,38 | 1,00 | 0,24 | 0,03 | 1,00 | 0,30 | 0,77 | 0,39 | 18,25 |
| 14 | 102010077 | C | T | missense | 0,000004010 | 30/78 | 2071 | P/L | healthy | motor domain | 26,10 | 5,85 | 0,48 | 1,00 | 0,47 | 0,04 | 1,00 | 0,29 | 0,77 | 0,37 | 16,42 |
| 14 | 102010081 | T | G | missense | 0,000004020 | 30/78 | 2072 | F/L | healthy | motor domain | 18,37 | 0,92 | 0,42 | 1,00 | 0,29 | 0,41 | 0,02 | 0,30 | 0,76 | 0,39 | 18,94 |
| 14 | 102010283 | G | A | missense | 0,000003990 | 31/78 | 2077 | D/N | healthy | motor domain | 22,80 | 5,61 | 0,35 | 1,00 | 0,23 | 0,47 | 0,13 | 0,26 | 0,77 | 0,34 | 13,71 |
| 14 | 102010314 | A | G | missense | 0,000000000 | 31/78 | 2087 | D/G | patient | motor domain | 29,60 | 5,61 | 0,41 | 1,00 | 0,68 | 0,00 | 0,99 | 0,15 | 0,78 | 0,20 | 3,31 |
| 14 | 102010325 | C | T | missense | 0,000000000 | 31/78 | 2091 | R/W | patient | motor domain | 29,80 | 4,67 | 0,77 | 1,00 | 0,72 | 0,00 | 1,00 | NA | NA |  | NA |
| 14 | 102010392 | G | A | missense | 0,000011900 | 31/78 | 2113 | R/K | healthy | motor domain | 22,20 | 5,61 | 0,39 | 0,97 | 0,05 | 0,55 | 0,00 | 0,13 | 0,78 | 0,16 | 1,89 |
| 14 | 102010755 | G | A | missense | 0,000023900 | 32/78 | 2141 | V/I | healthy | motor domain | 22,90 | 4,72 | 0,44 | 1,00 | 0,16 | 0,75 | 0,02 | 0,27 | 0,76 | 0,36 | 15,26 |
| 14 | 102010765 | C | T | missense | 0,000007960 | 32/78 | 2144 | T/M | healthy | motor domain | 26,90 | 5,62 | 0,39 | 1,00 | 0,35 | 0,00 | 1,00 | 0,30 | 0,76 | 0,40 | 19,20 |
| 14 | 102010798 | C | T | missense | 0,000003980 | 32/78 | 2155 | P/L | healthy | motor domain | 27,80 | 4,59 | 0,50 | 1,00 | 0,49 | 0,03 | 0,91 | 0,39 | 0,76 | 0,51 | 35,79 |
| 14 | 102010819 | C | T | missense | 0,000003980 | 32/78 | 2162 | S/L | healthy | motor domain | 23,30 | 5,49 | 0,50 | 1,00 | 0,33 | 0,16 | 0,01 | 0,30 | 0,75 | 0,40 | 20,08 |
| 14 | 102010824 | G | A | missense | 0,000000000 | 32/78 | 2164 | V/M | patient | motor domain | 31,00 | 5,49 | 0,37 | 1,00 | 0,39 | 0,00 | 0,91 | 0,36 | 0,75 | 0,49 | 32,52 |
| 14 | 102010840 | A | C | missense | 0,000011900 | 32/78 | 2169 | Q/P | healthy | motor domain | 23,40 | 5,49 | 0,42 | 1,00 | 0,17 | 0,13 | 0,01 | 0,42 | 0,76 | 0,55 | 43,56 |
| 14 | 102010870 | G | A | missense | 0,000003980 | 32/78 | 2179 | R/Q | healthy | motor domain | 24,50 | 5,49 | 0,41 | 1,00 | 0,28 | 0,05 | 0,42 | 0,40 | 0,77 | 0,52 | 37,94 |
| 14 | 102010873 | A | C | missense | 0,000003980 | 32/78 | 2180 | E/A | healthy | motor domain | 23,40 | 5,49 | 0,00 | 1,00 | 0,26 | 0,44 | 0,00 | 0,46 | 0,76 | 0,61 | 54,12 |
| 14 | 102010909 | C | T | missense | 0,000023900 | 32/78 | 2192 | T/I | healthy | motor domain | 22,70 | 5,49 | 0,46 | 1,00 | 0,20 | 0,20 | 0,01 | 0,40 | 0,78 | 0,51 | 36,93 |
| 14 | 102010918 | A | C | missense | 0,000007950 | 32/78 | 2195 | D/A | healthy | motor domain | 24,80 | 5,49 | 0,43 | 1,00 | 0,31 | 0,00 | 0,08 | 0,40 | 0,78 | 0,51 | 36,93 |
| 14 | 102010918 | A | G | missense | 0,000007950 | 32/78 | 2195 | D/G | healthy | motor domain | 24,80 | 5,49 | 0,47 | 1,00 | 0,32 | 0,00 | 0,08 | 0,40 | 0,78 | 0,51 | 36,93 |
| 14 | 102010938 | A | G | missense | 0,000003980 | 32/78 | 2202 | M/V | healthy | motor domain | 22,70 | 5,49 | 0,45 | 1,00 | 0,21 | 0,40 | 0,03 | 0,40 | 0,79 | 0,50 | 35,44 |
| 14 | 102011884 | C | A | missense | 0,000003990 | 33/78 | 2210 | L/I | healthy | motor domain | 25,70 | 4,92 | 0,46 | 1,00 | 0,27 | 0,00 | 0,85 | 0,39 | 0,77 | 0,50 | 34,47 |
| 14 | 102011888 | A | G | missense | 0,000003990 | 33/78 | 2211 | Y/C | healthy | motor domain | 26,10 | 5,80 | 0,63 | 1,00 | 0,55 | 0,00 | 0,34 | 0,25 | 0,77 | 0,32 | 12,01 |
| 14 | 102011960 | G | A | missense | 0,000015900 | 33/78 | 2235 | R/H | healthy | motor domain | 34,00 | 5,06 | 0,62 | 1,00 | 0,32 | 0,00 | 0,89 | 0,24 | 0,76 | 0,31 | 11,10 |
| 14 | 102011977 | T | A | missense | 0,000003980 | 33/78 | 2241 | L/M | healthy | motor domain | 22,60 | -0,94 | 0,67 | 1,00 | 0,37 | 0,09 | 0,84 | 0,37 | 0,76 | 0,48 | 31,70 |
| 14 | 102011986 | C | T | missense | 0,000003980 | 33/78 | 2244 | L/F | healthy | motor domain | 20,90 | 4,86 | 0,50 | 1,00 | 0,46 | 0,26 | 0,04 | 0,44 | 0,76 | 0,58 | 48,57 |
| 14 | 102011995 | G | A | missense | 0,000003980 | 33/78 | 2247 | V/M | healthy | motor domain | 24,20 | 5,95 | 0,42 | 1,00 | 0,35 | 0,04 | 0,32 | 0,54 | 0,76 | 0,71 | 73,14 |
| 14 | 102012010 | C | T | missense | 0,000003980 | 33/78 | 2252 | H/Y | healthy | motor domain | 22,30 | 5,06 | 0,53 | 1,00 | 0,29 | 0,48 | 0,08 | 0,55 | 0,76 | 0,72 | 76,50 |
| 14 | 102012016 | A | G | missense | 0,000003980 | 33/78 | 2254 | I/V | healthy | motor domain | 26,20 | 5,95 | 0,49 | 1,00 | 0,47 | 0,04 | 0,67 | 0,55 | 0,76 | 0,72 | 76,63 |
| 14 | 102012019 | G | A | missense | 0,000039800 | 33/78 | 2255 | D/N | healthy | motor domain | 32,00 | 5,07 | 0,00 | 1,00 | 0,51 | 0,01 | 0,60 | 0,60 | 0,76 | 0,80 | 87,00 |
| 14 | 102012083 | C | T | missense | 0,000003980 | 33/78 | 2276 | T/I | healthy | motor domain | 27,10 | 5,96 | 0,61 | 1,00 | 0,51 | 0,00 | 1,00 | 0,36 | 0,77 | 0,47 | 30,17 |
| 14 | 102012103 | G | T | missense | 0,000003980 | 33/78 | 2283 | V/L | healthy | motor domain | 23,30 | 5,08 | 0,65 | 1,00 | 0,17 | 0,09 | 0,01 | 0,38 | 0,77 | 0,49 | 32,69 |
| 14 | 102012103 | G | A | missense | 0,000023900 | 33/78 | 2283 | V/M | healthy | motor domain | 25,10 | 5,08 | 0,00 | 1,00 | 0,23 | 0,00 | 0,40 | 0,38 | 0,77 | 0,49 | 32,69 |
| 14 | 102012327 | G | A | missense | 0,000003980 | 34/78 | 2291 | V/M | healthy | motor domain | 26,30 | 5,59 | 0,43 | 1,00 | 0,26 | 0,00 | 0,99 | 0,43 | 0,79 | 0,55 | 42,59 |
| 14 | 102012336 | G | A | missense | 0,000000000 | 34/78 | 2294 | E/K | study patient | motor domain | 26,80 | 5,57 | 0,61 | 1,00 | 0,73 | 0,00 | 1,00 | NA | NA | NA | NA |
| 14 | 102012339 | C | G | missense | 0,000003980 | 34/78 | 2295 | L/V | healthy | motor domain | 14,17 | 2,76 | 0,48 | 1,00 | 0,07 | 0,28 | 0,17 | NA | NA | NA | NA |
| 14 | 102012340 | T | G | missense | 0,000003980 | 34/78 | 2295 | L/R | healthy | motor domain | 21,70 | 5,57 | 0,73 | 1,00 | 0,22 | 0,54 | 0,03 | 0,40 | 0,79 | 0,51 | 36,76 |
| 14 | 102012357 | A | G | missense | 0,000003980 | 34/78 | 2301 | I/V | healthy | motor domain | 23,70 | 3,33 | 0,58 | 1,00 | 0,14 | 0,04 | 0,51 | 0,33 | 0,78 | 0,43 | 23,48 |
| 14 | 102012360 | G | A | missense | 0,000000000 | 34/78 | 2302 | V/I | healthy | motor domain | 6,16 | -1,22 | 0,59 | 0,85 | 0,07 | 1,00 | 0,00 | 0,33 | 0,79 | 0,43 | 23,03 |
| 14 | 102012372 | G | A | missense | 0,000003980 | 34/78 | 2306 | D/N | healthy | motor domain | 28,20 | 4,80 | 0,64 | 1,00 | 0,61 | 0,00 | 1,00 | 0,35 | 0,78 | 0,45 | 27,48 |
| 14 | 102012442 | A | G | missense | 0,000003980 | 34/78 | 2329 | N/S | healthy | motor domain | 24,70 | 5,70 | 0,00 | 1,00 | 0,47 | 0,08 | 0,99 | 0,30 | 0,77 | 0,39 | 18,08 |
| 14 | 102012445 | G | A | missense | 0,000000000 | 34/78 | 2330 | G/E | patient | motor domain | 32,00 | 5,70 | 0,71 | 1,00 | 0,85 | 0,00 | 1,00 | 0,30 | 0,78 | 0,39 | 17,73 |
| 14 | 102012450 | C | T | missense | 0,000000000 | 34/78 | 2332 | R/C | patient | motor domain | 33,00 | 5,70 | 0,58 | 1,00 | 0,95 | 0,00 | 1,00 | 0,30 | 0,77 | 0,39 | 18,77 |
| 14 | 102012465 | C | T | missense | 0,000015900 | 34/78 | 2337 | P/S | healthy | motor domain | 23,00 | 5,70 | 0,60 | 1,00 | 0,61 | 0,25 | 0,31 | 0,27 | 0,75 | 0,36 | 16,08 |
| 14 | 102015105 | G | T | missense | 0,000003980 | 35/78 | 2339 | V/L | healthy | motor domain | 33,00 | 5,56 | 0,56 | 1,00 | 0,21 | 0,02 | 0,58 | 0,27 | 0,76 | 0,36 | 15,71 |
| 14 | 102015154 | C | T | missense | 0,000000000 | 35/78 | 2355 | T/I | patient | motor domain | 24,50 | 5,56 | 0,59 | 1,00 | 0,92 | 0,00 | 1,00 | 0,30 | 0,78 | 0,38 | 17,50 |
| 14 | 102015162 | C | T | missense | 0,000000000 | 35/78 | 2358 | R/C | patient | motor domain | 25,90 | 4,35 | 0,86 | 1,00 | 0,65 | 0,00 | 1,00 | 0,31 | 0,80 | 0,38 | 17,60 |
| 14 | 102015169 | G | C | missense | 0,000003980 | 35/78 | 2360 | G/A | healthy | motor domain | 28,60 | 5,32 | 0,00 | 1,00 | 0,80 | 0,00 | 1,00 | 0,33 | 0,79 | 0,42 | 22,27 |
| 14 | 102015191 | T | A | missense | 0,000003980 | 35/78 | 2367 | D/E | healthy | motor domain | 16,58 | -5,00 | 0,43 | 1,00 | 0,20 | 0,23 | 0,07 | 0,41 | 0,77 | 0,54 | 40,65 |
| 14 | 102015192 | G | A | missense | 0,000003980 | 35/78 | 2368 | V/M | healthy | motor domain | 25,70 | 5,32 | 0,49 | 1,00 | 0,20 | 0,03 | 0,21 | 0,47 | 0,77 | 0,62 | 55,93 |
| 14 | 102015206 | C | G | missense | 0,000003980 | 35/78 | 2372 | D/E | healthy | motor domain | 20,40 | 5,32 | 0,43 | 1,00 | 0,17 | 1,00 | 0,00 | 0,50 | 0,77 | 0,65 | 62,62 |
| 14 | 102015210 | A | G | missense | 0,000003980 | 35/78 | 2374 | I/V | healthy | motor domain | 22,00 | 5,32 | 0,43 | 1,00 | 0,07 | 0,46 | 0,02 | 0,52 | 0,78 | 0,67 | 67,94 |
| 14 | 102015217 | A | G | missense | 0,000003980 | 35/78 | 2376 | N/S | healthy | motor domain | 22,60 | 5,32 | 0,32 | 1,00 | 0,10 | 0,54 | 0,01 | 0,52 | 0,78 | 0,67 | 67,94 |
| 14 | 102015228 | G | A | missense | 0,000015900 | 35/78 | 2380 | A/T | healthy | motor domain | 23,30 | 5,32 | 0,00 | 1,00 | 0,08 | 0,45 | 0,00 | 0,57 | 0,78 | 0,73 | 77,69 |
| 14 | 102015238 | G | A | missense | 0,000007950 | 35/78 | 2383 | R/H | healthy | motor domain | 25,90 | 5,32 | 0,45 | 1,00 | 0,13 | 0,03 | 0,17 | 0,58 | 0,78 | 0,74 | 79,08 |
| 14 | 102015237 | C | T | missense | 0,000015900 | 35/78 | 2383 | R/C | healthy | motor domain | 28,90 | 5,32 | 0,52 | 1,00 | 0,21 | 0,00 | 0,86 | 0,58 | 0,78 | 0,74 | 79,08 |
| 14 | 102015241 | G | A | missense | 0,000003980 | 35/78 | 2384 | S/N | healthy | motor domain | 21,70 | 5,32 | 0,27 | 1,00 | 0,09 | 0,87 | 0,00 | 0,56 | 0,78 | 0,72 | 75,25 |
| 14 | 102015246 | C | T | missense | 0,000003980 | 35/78 | 2386 | P/S | healthy | motor domain | 22,90 | 5,32 | 0,33 | 1,00 | 0,23 | 0,14 | 0,14 | 0,57 | 0,77 | 0,74 | 79,21 |
| 14 | 102015250 | T | C | missense | 0,000003980 | 35/78 | 2387 | L/P | healthy | motor domain | 32,00 | 5,32 | 0,46 | 1,00 | 0,45 | 0,00 | 0,94 | 0,62 | 0,77 | 0,80 | 87,00 |
| 14 | 102015271 | C | G | missense | 0,000003980 | 35/78 | 2394 | A/G | healthy | motor domain | 22,90 | 5,32 | 0,30 | 1,00 | 0,08 | 0,23 | 0,00 | 0,62 | 0,76 | 0,81 | 88,75 |
| 14 | 102015275 | G | T | missense | 0,000003980 | 35/78 | 2395 | Q/H | healthy | motor domain | 21,50 | 4,42 | 0,34 | 1,00 | 0,05 | 0,30 | 0,02 | 0,62 | 0,76 | 0,81 | 88,75 |
| 14 | 102015280 | G | A | missense | 0,000003980 | 35/78 | 2397 | R/Q | healthy | motor domain | 22,20 | 5,32 | 0,23 | 1,00 | 0,06 | 0,61 | 0,00 | 0,64 | 0,76 | 0,85 | 92,19 |
| 14 | 102015283 | G | A | missense | 0,000023900 | 35/78 | 2398 | R/H | healthy | motor domain | 23,50 | 4,43 | 0,00 | 1,00 | 0,14 | 0,17 | 0,03 | 0,64 | 0,76 | 0,85 | 92,19 |
| 14 | 102015282 | C | T | missense | 0,000000000 | 35/78 | 2398 | R/C | healthy | motor domain | 23,70 | 5,32 | 0,00 | 1,00 | 0,16 | 0,06 | 0,08 | 0,64 | 0,76 | 0,85 | 92,19 |
| 14 | 102015288 | G | A | missense | 0,000007970 | 35/78 | 2400 | G/S | healthy | motor domain | 22,40 | 4,43 | 0,29 | 1,00 | 0,05 | 0,80 | 0,00 | 0,60 | 0,76 | 0,79 | 86,74 |
| 14 | 102015293 | A | C | missense | 0,000000000 | 35/78 | 2401 | K/N | healthy | motor domain | 11,89 | -3,31 | 0,00 | 1,00 | 0,22 | 0,48 | 0,00 | 0,60 | 0,76 | 0,79 | 86,74 |
| 14 | 102015297 | G | A | missense | 0,000031900 | 35/78 | 2403 | D/N | healthy | motor domain | 23,80 | 5,32 | 0,18 | 1,00 | 0,11 | 0,30 | 0,00 | 0,59 | 0,77 | 0,77 | 83,99 |
| 14 | 102015302 | G | C | missense | 0,000003990 | 35/78 | 2404 | E/D | healthy | motor domain | 15,27 | 0,75 | 0,30 | 1,00 | 0,33 | 0,58 | 0,00 | NA | NA | NA | NA |
| 14 | 102015315 | G | A | missense | 0,000020200 | 35/78 | 2409 | A/T | healthy | motor domain | 22,30 | 5,59 | 0,37 | 1,00 | 0,09 | 0,32 | 0,00 | 0,56 | 0,77 | 0,72 | 76,26 |
| 14 | 102015324 | A | G | missense | 0,000004060 | 35/78 | 2412 | M/V | healthy | motor domain | 20,60 | 5,59 | 0,38 | 1,00 | 0,24 | 0,55 | 0,00 | 0,54 | 0,76 | 0,71 | 73,06 |
| 14 | 102015325 | T | A | missense | 0,000004070 | 35/78 | 2412 | M/K | healthy | motor domain | 22,60 | 5,59 | 0,53 | 1,00 | 0,23 | 0,43 | 0,02 | 0,54 | 0,76 | 0,71 | 73,06 |
| 14 | 102015326 | G | A | missense | 0,000004080 | 35/78 | 2412 | M/I | healthy | motor domain | 22,80 | 5,59 | 0,39 | 1,00 | 0,18 | 0,29 | 0,01 | 0,54 | 0,76 | 0,71 | 73,06 |
| 14 | 102015869 | C | T | missense | 0,000003980 | 36/78 | 2419 | A/V | healthy | motor domain | 18,54 | 5,26 | 0,42 | 1,00 | 0,21 | 1,00 | 0,00 | 0,44 | 0,77 | 0,58 | 49,17 |
| 14 | 102015875 | C | T | missense | 0,000011900 | 36/78 | 2421 | T/M | healthy | motor domain | 20,20 | 5,26 | 0,35 | 0,86 | 0,12 | 0,06 | 0,06 | 0,46 | 0,77 | 0,61 | 54,12 |
| 14 | 102015887 | C | T | missense | 0,000003980 | 36/78 | 2425 | P/L | healthy | motor domain | 23,60 | 5,26 | 0,44 | 1,00 | 0,28 | 0,03 | 0,02 | 0,50 | 0,76 | 0,66 | 65,25 |
| 14 | 102015896 | C | T | missense | 0,000019900 | 36/78 | 2428 | T/M | healthy | motor domain | 24,10 | 5,50 | 0,34 | 1,00 | 0,20 | 0,04 | 0,44 | 0,44 | 0,77 | 0,58 | 49,17 |
| 14 | 102015899 | C | G | missense | 0,000003980 | 36/78 | 2429 | S/C | healthy | motor domain | 23,20 | 3,62 | 0,50 | 1,00 | 0,20 | 0,03 | 0,02 | 0,44 | 0,76 | 0,58 | 49,69 |
| 14 | 102015904 | G | A | missense | 0,000007960 | 36/78 | 2431 | G/S | healthy | motor domain | 25,60 | 5,50 | 0,39 | 1,00 | 0,24 | 0,07 | 0,88 | 0,48 | 0,76 | 0,63 | 58,55 |
| 14 | 102015910 | G | A | missense | 0,000007950 | 36/78 | 2433 | V/I | healthy | motor domain | 23,40 | 5,50 | 0,32 | 1,00 | 0,19 | 0,29 | 0,38 | 0,52 | 0,75 | 0,69 | 70,43 |
| 14 | 102015920 | C | T | missense | 0,000003980 | 36/78 | 2436 | A/V | healthy | motor domain | 23,10 | 5,50 | 0,42 | 1,00 | 0,14 | 0,08 | 0,05 | 0,54 | 0,75 | 0,72 | 75,60 |
| 14 | 102015919 | G | A | missense | 0,000007960 | 36/78 | 2436 | A/T | healthy | motor domain | 23,50 | 5,50 | 0,38 | 1,00 | 0,13 | 0,17 | 0,02 | 0,54 | 0,75 | 0,72 | 75,60 |
| 14 | 102015929 | A | G | missense | 0,000003980 | 36/78 | 2439 | H/R | healthy | motor domain | 22,80 | 5,50 | 0,43 | 1,00 | 0,30 | 0,24 | 0,05 | 0,54 | 0,74 | 0,73 | 77,99 |
| 14 | 102015931 | G | A | missense | 0,000003980 | 36/78 | 2440 | A/T | healthy | motor domain | 28,70 | 5,50 | 0,37 | 1,00 | 0,27 | 0,01 | 0,91 | 0,57 | 0,74 | 0,76 | 82,72 |
| 14 | 102015931 | G | T | missense | 0,000007960 | 36/78 | 2440 | A/S | healthy | motor domain | 32,00 | 5,50 | 0,37 | 1,00 | 0,35 | 0,00 | 0,96 | 0,57 | 0,74 | 0,76 | 82,72 |
| 14 | 102015949 | A | T | missense | 0,000003980 | 36/78 | 2446 | I/F | healthy | motor domain | 25,30 | 5,50 | 0,42 | 1,00 | 0,48 | 0,00 | 0,14 | 0,58 | 0,75 | 0,78 | 84,70 |
| 14 | 102015953 | T | C | missense | 0,000003980 | 36/78 | 2447 | M/T | healthy | motor domain | 28,20 | 5,50 | 0,46 | 1,00 | 0,60 | 0,00 | 0,97 | 0,56 | 0,74 | 0,75 | 80,35 |
| 14 | 102015958 | C | G | missense | 0,000003980 | 36/78 | 2449 | L/V | healthy | motor domain | 22,20 | 4,59 | 0,41 | 0,98 | 0,03 | 0,07 | 0,01 | 0,56 | 0,74 | 0,76 | 82,14 |
| 14 | 102015980 | G | A | missense | 0,000003990 | 36/78 | 2456 | G/D | healthy | motor domain | 24,10 | 5,54 | 0,49 | 1,00 | 0,32 | 0,14 | 0,11 | 0,50 | 0,76 | 0,66 | 65,07 |
| 14 | 102016007 | C | T | missense | 0,000003990 | 36/78 | 2465 | A/V | healthy | motor domain | 23,50 | 5,54 | 0,44 | 1,00 | 0,22 | 0,06 | 0,08 | 0,50 | 0,78 | 0,64 | 61,24 |
| 14 | 102016013 | G | T | missense | 0,000012000 | 36/78 | 2467 | R/L | healthy | motor domain | 33,00 | 5,54 | 0,47 | 1,00 | 0,63 | 0,00 | 0,78 | 0,55 | 0,77 | 0,71 | 74,52 |
| 14 | 102016016 | A | T | missense | 0,000016000 | 36/78 | 2468 | N/I | healthy | motor domain | 27,50 | 5,54 | 0,47 | 1,00 | 0,36 | 0,00 | 0,55 | 0,52 | 0,76 | 0,68 | 69,55 |
| 14 | 102016022 | C | G | missense | 0,000004000 | 36/78 | 2470 | A/G | healthy | motor domain | 21,60 | 5,54 | 0,38 | 1,00 | 0,19 | 0,28 | 0,00 | 0,56 | 0,77 | 0,73 | 77,99 |
| 14 | 102016022 | C | T | missense | 0,000012000 | 36/78 | 2470 | A/V | healthy | motor domain | 23,30 | 5,54 | 0,38 | 1,00 | 0,18 | 0,01 | 0,00 | 0,56 | 0,77 | 0,73 | 77,99 |
| 14 | 102016034 | C | T | missense | 0,000004000 | 36/78 | 2474 | A/V | healthy | motor domain | 21,30 | 5,54 | 0,37 | 1,00 | 0,10 | 0,62 | 0,00 | 0,54 | 0,77 | 0,70 | 71,81 |
| 14 | 102016033 | G | A | missense | 0,000020000 | 36/78 | 2474 | A/T | healthy | motor domain | 22,20 | 4,58 | 0,31 | 1,00 | 0,05 | 0,59 | 0,01 | 0,54 | 0,77 | 0,70 | 71,81 |
| 14 | 102016037 | A | G | missense | 0,000008000 | 36/78 | 2475 | N/S | healthy | motor domain | 21,80 | 5,54 | 0,00 | 1,00 | 0,14 | 0,54 | 0,00 | 0,56 | 0,77 | 0,73 | 77,69 |
| 14 | 102016039 | C | T | missense | 0,000004000 | 36/78 | 2476 | H/Y | healthy | motor domain | 25,20 | 5,54 | 0,43 | 1,00 | 0,57 | 0,01 | 0,78 | 0,56 | 0,78 | 0,72 | 76,63 |
| 14 | 102016054 | A | G | missense | 0,000012100 | 36/78 | 2481 | M/V | healthy | motor domain | 22,70 | 5,54 | 0,30 | 1,00 | 0,24 | 0,07 | 0,01 | 0,52 | 0,78 | 0,67 | 66,30 |
| 14 | 102016061 | T | C | missense | 0,000012100 | 36/78 | 2483 | I/T | healthy | motor domain | 20,90 | 4,37 | 0,46 | 1,00 | 0,08 | 0,52 | 0,02 | 0,54 | 0,77 | 0,70 | 71,94 |
| 14 | 102016063 | G | A | missense | 0,000004090 | 36/78 | 2484 | E/K | healthy | motor domain | 25,80 | 5,54 | 0,00 | 1,00 | 0,15 | 0,02 | 0,01 | 0,52 | 0,77 | 0,68 | 69,08 |
| 14 | 102016066 | C | G | missense | 0,000004080 | 36/78 | 2485 | Q/E | healthy | motor domain | 23,70 | 5,54 | 0,27 | 1,00 | 0,15 | 0,02 | 0,37 | 0,52 | 0,77 | 0,68 | 68,93 |
| 14 | 102016076 | G | A | missense | 0,000000000 | 36/78 | 2488 | R/H | healthy | motor domain | 23,50 | 5,54 | 0,00 | 1,00 | 0,10 | 0,10 | 0,00 | 0,52 | 0,76 | 0,69 | 70,43 |
| 14 | 102016350 | G | A | missense | 0,000007950 | 37/78 | 2492 | R/Q | healthy | motor domain | 25,90 | 5,54 | 0,00 | 1,00 | 0,10 | 0,00 | 0,08 | 0,47 | 0,77 | 0,62 | 55,78 |
| 14 | 102016367 | A | G | missense | 0,000003980 | 37/78 | 2498 | I/V | healthy | motor domain | 22,90 | 5,54 | 0,40 | 1,00 | 0,11 | 0,16 | 0,00 | 0,47 | 0,76 | 0,62 | 56,49 |
| 14 | 102016395 | G | A | missense | 0,000003980 | 37/78 | 2507 | R/Q | healthy | motor domain | 24,20 | 4,78 | 0,39 | 1,00 | 0,19 | 0,15 | 0,23 | 0,54 | 0,73 | 0,74 | 79,58 |
| 14 | 102016414 | G | C | missense | 0,000011900 | 37/78 | 2513 | E/D | healthy | motor domain | 20,90 | 5,67 | 0,00 | 1,00 | 0,13 | 0,53 | 0,01 | 0,57 | 0,72 | 0,79 | 86,23 |
| 14 | 102016418 | G | T | missense | 0,000003980 | 37/78 | 2515 | G/C | healthy | motor domain | 32,00 | 5,67 | 0,51 | 1,00 | 0,52 | 0,02 | 0,89 | 0,56 | 0,73 | 0,77 | 84,38 |
| 14 | 102016427 | A | G | missense | 0,000003980 | 37/78 | 2518 | I/V | healthy | motor domain | 21,70 | 5,67 | 0,41 | 1,00 | 0,14 | 0,63 | 0,02 | 0,56 | 0,74 | 0,75 | 81,26 |
| 14 | 102016440 | C | T | missense | 0,000007950 | 37/78 | 2522 | T/M | healthy | motor domain | 26,40 | 4,78 | 0,00 | 1,00 | 0,40 | 0,00 | 0,92 | 0,53 | 0,74 | 0,71 | 74,72 |
| 14 | 102016445 | G | A | missense | 0,000007950 | 37/78 | 2524 | V/M | healthy | motor domain | 24,70 | 4,65 | 0,00 | 1,00 | 0,12 | 0,07 | 0,58 | 0,53 | 0,74 | 0,71 | 74,41 |
| 14 | 102016458 | C | G | missense | 0,000003980 | 37/78 | 2528 | T/S | healthy | motor domain | 16,85 | 3,45 | 0,34 | 1,00 | 0,07 | 0,53 | 0,00 | 0,57 | 0,75 | 0,76 | 82,72 |
| 14 | 102016461 | C | T | missense | 0,000011900 | 37/78 | 2529 | A/V | healthy | motor domain | 21,80 | 5,38 | 0,38 | 1,00 | 0,18 | 0,30 | 0,01 | 0,52 | 0,76 | 0,69 | 70,78 |
| 14 | 102016464 | C | G | missense | 0,000003980 | 37/78 | 2530 | P/R | healthy | motor domain | 22,80 | 5,38 | 0,58 | 1,00 | 0,24 | 0,15 | 0,05 | 0,52 | 0,75 | 0,70 | 71,72 |
| 14 | 102016467 | A | G | missense | 0,000023900 | 37/78 | 2531 | N/S | healthy | motor domain | 19,63 | 5,54 | 0,33 | 1,00 | 0,10 | 0,67 | 0,00 | 0,50 | 0,75 | 0,67 | 66,86 |
| 14 | 102016776 | G | C | missense | 0,000004010 | 38/78 | 2542 | S/T | healthy | motor domain | 18,62 | 4,41 | 0,29 | 1,00 | 0,09 | 0,58 | 0,00 | 0,44 | 0,75 | 0,59 | 51,24 |
| 14 | 102016776 | G | A | missense | 0,000004010 | 38/78 | 2542 | S/N | healthy | motor domain | 19,33 | 4,41 | 0,23 | 1,00 | 0,08 | 0,45 | 0,00 | 0,44 | 0,75 | 0,59 | 51,24 |
| 14 | 102016778 | G | A | missense | 0,000016000 | 38/78 | 2543 | G/R | healthy | motor domain | 27,00 | 5,35 | 0,00 | 1,00 | 0,66 | 0,00 | 0,99 | 0,44 | 0,75 | 0,59 | 51,24 |
| 14 | 102016790 | C | T | missense | 0,000004010 | 38/78 | 2547 | P/S | healthy | motor domain | 18,70 | 4,50 | 0,35 | 1,00 | 0,09 | 0,46 | 0,01 | 0,42 | 0,76 | 0,55 | 42,59 |
| 14 | 102016832 | A | G | missense | 0,000003990 | 38/78 | 2561 | K/E | healthy | motor domain | 28,80 | 5,54 | 0,34 | 1,00 | 0,33 | 0,04 | 0,92 | 0,31 | 0,75 | 0,42 | 21,80 |
| 14 | 102016853 | G | A | missense | 0,000012000 | 38/78 | 2568 | V/I | healthy | motor domain | 24,10 | 5,54 | 0,32 | 1,00 | 0,18 | 0,11 | 0,94 | 0,37 | 0,75 | 0,49 | 33,31 |
| 14 | 102016856 | G | A | missense | 0,000003980 | 38/78 | 2569 | V/M | healthy | motor domain | 26,80 | 5,54 | 0,00 | 1,00 | 0,46 | 0,00 | 1,00 | 0,39 | 0,75 | 0,52 | 38,15 |
| 14 | 102016878 | G | A | missense | 0,000003980 | 38/78 | 2576 | R/H | healthy | motor domain | 33,00 | 5,37 | 0,67 | 1,00 | 0,76 | 0,00 | 1,00 | 0,35 | 0,73 | 0,47 | 30,17 |
| 14 | 102016883 | G | A | missense | 0,000003980 | 38/78 | 2578 | E/K | healthy | motor domain | 32,00 | 5,37 | 0,66 | 1,00 | 0,49 | 0,00 | 0,99 | 0,32 | 0,73 | 0,44 | 24,90 |
| 14 | 102016886 | G | A | missense | 0,000003980 | 38/78 | 2579 | A/T | healthy | motor domain | 23,70 | 5,37 | 0,00 | 1,00 | 0,17 | 0,08 | 0,03 | 0,33 | 0,72 | 0,46 | 28,58 |
| 14 | 102016889 | C | G | missense | 0,000007960 | 38/78 | 2580 | L/V | healthy | motor domain | 21,70 | 5,37 | 0,43 | 1,00 | 0,20 | 1,00 | 0,61 | 0,35 | 0,72 | 0,48 | 32,02 |
| 14 | 102016908 | C | G | missense | 0,000003980 | 38/78 | 2586 | A/G | healthy | motor domain | 25,00 | 5,37 | 0,36 | 1,00 | 0,18 | 0,00 | 0,84 | 0,31 | 0,73 | 0,43 | 23,35 |
| 14 | 102016944 | G | T | missense | 0,000000000 | 38/78 | 2598 | G/V | study patient | motor domain | 29,50 | 5,16 | 0,85 | 1,00 | 0,95 | 0,00 | 1,00 | 0,20 | 0,74 | 0,27 | 8,20 |
| 14 | 102016982 | G | A | missense | 0,000003990 | 38/78 | 2611 | A/T | healthy | motor domain | 27,40 | 5,16 | 0,44 | 1,00 | 0,29 | 0,10 | 0,98 | 0,38 | 0,73 | 0,51 | 37,27 |
| 14 | 102016994 | A | C | missense | 0,000003980 | 38/78 | 2615 | M/L | healthy | motor domain | 23,30 | 5,16 | 0,54 | 1,00 | 0,23 | 0,23 | 0,04 | 0,44 | 0,73 | 0,61 | 54,27 |
| 14 | 102017103 | T | C | missense | 0,000003980 | 39/78 | 2622 | F/L | healthy | motor domain | 32,00 | 4,92 | 0,44 | 1,00 | 0,63 | 0,00 | 1,00 | 0,46 | 0,76 | 0,60 | 52,81 |
| 14 | 102017121 | C | A | missense | 0,000003980 | 39/78 | 2628 | P/T | healthy | motor domain | 24,20 | 5,06 | 0,51 | 1,00 | 0,62 | 0,00 | 1,00 | 0,46 | 0,75 | 0,61 | 54,70 |
| 14 | 102017133 | C | A | missense | 0,000003980 | 39/78 | 2632 | L/M | healthy | motor domain | 23,10 | 4,17 | 0,47 | 1,00 | 0,13 | 0,16 | 0,65 | 0,36 | 0,75 | 0,48 | 30,41 |
| 14 | 102017164 | G | C | missense | 0,000003980 | 39/78 | 2642 | R/T | healthy | motor domain | 26,40 | 5,16 | 0,66 | 1,00 | 0,45 | 0,00 | 0,93 | 0,33 | 0,75 | 0,45 | 27,16 |
| 14 | 102017179 | G | A | missense | 0,000019900 | 39/78 | 2647 | G/E | healthy | motor domain | 25,70 | 5,16 | 0,57 | 1,00 | 0,59 | 0,00 | 1,00 | 0,27 | 0,76 | 0,35 | 14,38 |
| 14 | 102017181 | G | A | missense | 0,000003980 | 39/78 | 2648 | V/M | healthy | motor domain | 22,00 | 5,46 | 0,53 | 1,00 | 0,22 | 0,14 | 0,16 | 0,23 | 0,76 | 0,30 | 10,67 |
| 14 | 102017286 | A | C | missense | 0,000003980 | 39/78 | 2683 | I/L | healthy | motor domain | 21,40 | 5,46 | 0,62 | 1,00 | 0,18 | 1,00 | 0,02 | 0,46 | 0,79 | 0,57 | 48,10 |
| 14 | 102017383 | A | T | missense | 0,000007950 | 40/78 | 2686 | M/L | healthy | motor domain | 21,80 | 5,19 | 0,55 | 1,00 | 0,29 | 1,00 | 0,00 | 0,55 | 0,79 | 0,69 | 71,32 |
| 14 | 102017405 | A | G | missense | 0,000003980 | 40/78 | 2693 | Y/C | healthy | motor domain | 32,00 | 5,19 | 0,64 | 1,00 | 0,73 | 0,00 | 0,98 | 0,46 | 0,78 | 0,59 | 51,97 |
| 14 | 102017407 | C | T | missense | 0,000003980 | 40/78 | 2694 | R/C | healthy | motor domain | 31,00 | 5,19 | 0,60 | 1,00 | 0,48 | 0,00 | 1,00 | 0,46 | 0,77 | 0,60 | 52,81 |
| 14 | 102017408 | G | T | missense | 0,000003980 | 40/78 | 2694 | R/L | healthy | motor domain | 33,00 | 5,19 | 0,55 | 1,00 | 0,55 | 0,00 | 0,98 | 0,46 | 0,77 | 0,60 | 52,81 |
| 14 | 102017428 | G | T | missense | 0,000003980 | 40/78 | 2701 | V/L | healthy | motor domain | 27,80 | 5,19 | 0,44 | 1,00 | 0,19 | 0,00 | 0,73 | 0,43 | 0,77 | 0,56 | 45,02 |
| 14 | 102017467 | C | T | missense | 0,000003980 | 40/78 | 2714 | P/S | healthy | motor domain | 25,50 | 5,19 | 0,57 | 1,00 | 0,55 | 0,00 | 1,00 | 0,50 | 0,74 | 0,68 | 68,43 |
| 14 | 102017491 | C | G | missense | 0,000003980 | 40/78 | 2722 | P/A | healthy | motor domain | 25,30 | 5,19 | 0,67 | 1,00 | 0,39 | 0,02 | 0,96 | 0,50 | 0,74 | 0,68 | 68,65 |
| 14 | 102017494 | C | T | missense | 0,000003980 | 40/78 | 2723 | L/F | healthy | motor domain | 26,00 | 5,19 | 0,40 | 1,00 | 0,35 | 0,00 | 0,98 | 0,50 | 0,73 | 0,68 | 69,27 |
| 14 | 102017497 | T | G | missense | 0,000003980 | 40/78 | 2724 | S/A | healthy | motor domain | 22,70 | 5,19 | 0,48 | 1,00 | 0,08 | 0,13 | 0,04 | 0,47 | 0,74 | 0,64 | 60,49 |
| 14 | 102017501 | A | C | missense | 0,000003980 | 40/78 | 2725 | H/P | healthy | motor domain | 24,60 | 5,19 | 0,65 | 1,00 | 0,40 | 0,21 | 0,66 | 0,47 | 0,73 | 0,64 | 61,67 |
| 14 | 102018473 | G | A | missense | 0,000055800 | 41/78 | 2734 | V/M | healthy | motor domain | 29,90 | 5,26 | 0,00 | 1,00 | 0,45 | 0,02 | 0,98 | 0,50 | 0,73 | 0,69 | 70,09 |
| 14 | 102018477 | A | G | missense | 0,000003980 | 41/78 | 2735 | Y/C | healthy | motor domain | 33,00 | 5,26 | 0,65 | 1,00 | 0,56 | 0,01 | 0,99 | 0,48 | 0,74 | 0,65 | 62,26 |
| 14 | 102018482 | G | C | missense | 0,000003980 | 41/78 | 2737 | D/H | healthy | motor domain | 33,00 | 5,26 | 0,60 | 1,00 | 0,68 | 0,00 | 1,00 | 0,48 | 0,74 | 0,65 | 62,26 |
| 14 | 102018497 | G | A | missense | 0,000003980 | 41/78 | 2742 | A/T | healthy | motor domain | 23,20 | 5,26 | 0,36 | 1,00 | 0,24 | 0,86 | 0,01 | 0,33 | 0,73 | 0,46 | 28,51 |
| 14 | 102018503 | C | G | missense | 0,000011900 | 41/78 | 2744 | L/V | healthy | motor domain | 25,90 | 5,26 | 0,00 | 1,00 | 0,36 | 0,00 | 1,00 | 0,37 | 0,72 | 0,51 | 36,76 |
| 14 | 102018507 | C | A | missense | 0,000000000 | 41/78 | 2745 | T/K | study patient | motor domain | 21,90 | 5,26 | 0,52 | 1,00 | 0,33 | 1,00 | 0,00 | 0,37 | 0,73 | 0,51 | 35,96 |
| 14 | 102018518 | G | A | missense | 0,000011900 | 41/78 | 2749 | G/S | healthy | motor domain | 33,00 | 5,26 | 0,00 | 1,00 | 0,47 | 0,02 | 0,92 | 0,45 | 0,72 | 0,62 | 56,94 |
| 14 | 102018548 | A | G | missense | 0,000007960 | 41/78 | 2759 | I/V | healthy | motor domain | 17,71 | 2,88 | 0,50 | 1,00 | 0,12 | 0,81 | 0,01 | 0,39 | 0,73 | 0,52 | 38,69 |
| 14 | 102018561 | G | A | missense | 0,000011900 | 41/78 | 2763 | R/Q | healthy | motor domain | 27,70 | 5,26 | 0,46 | 1,00 | 0,34 | 0,00 | 0,97 | 0,36 | 0,75 | 0,48 | 31,18 |
| 14 | 102018564 | C | T | missense | 0,000011900 | 41/78 | 2764 | T/M | healthy | motor domain | 24,60 | 5,26 | 0,57 | 1,00 | 0,31 | 0,00 | 0,91 | 0,40 | 0,75 | 0,53 | 40,13 |
| 14 | 102018599 | T | A | missense | 0,000007960 | 41/78 | 2776 | F/I | healthy | motor domain | 33,00 | 5,26 | 0,42 | 1,00 | 0,49 | 0,03 | 0,97 | 0,42 | 0,75 | 0,56 | 44,72 |
| 14 | 102018609 | T | C | missense | 0,000003980 | 41/78 | 2779 | M/T | healthy | motor domain | 23,10 | 5,26 | 0,00 | 1,00 | 0,24 | 0,48 | 0,05 | 0,40 | 0,76 | 0,52 | 38,69 |
| 14 | 102019921 | A | T | missense | 0,000007950 | 42/78 | 2791 | H/L | healthy | motor domain | 29,30 | 5,66 | 0,00 | 1,00 | 0,73 | 0,00 | 1,00 | 0,57 | 0,78 | 0,73 | 78,22 |
| 14 | 102019965 | A | G | missense | 0,000003980 | 42/78 | 2806 | I/V | healthy | motor domain | 22,90 | 5,66 | 0,60 | 1,00 | 0,18 | 0,36 | 0,34 | 0,67 | 0,75 | 0,89 | 94,84 |
| 14 | 102019975 | C | T | missense | 0,000003980 | 42/78 | 2809 | A/V | healthy | motor domain | 26,80 | 5,66 | 0,69 | 1,00 | 0,51 | 0,00 | 1,00 | 0,70 | 0,75 | 0,94 | 96,84 |
| 14 | 102019981 | G | T | missense | 0,000003980 | 42/78 | 2811 | R/I | healthy | motor domain | 26,10 | 5,66 | 0,54 | 1,00 | 0,41 | 0,03 | 0,36 | 0,64 | 0,74 | 0,86 | 93,24 |
| 14 | 102019991 | G | C | missense | 0,000003980 | 42/78 | 2814 | E/D | healthy | motor domain | 21,90 | 3,82 | 0,45 | 1,00 | 0,18 | 0,42 | 0,02 | 0,58 | 0,74 | 0,79 | 86,53 |
| 14 | 102019993 | C | T | missense | 0,000003980 | 42/78 | 2815 | T/I | healthy | motor domain | 24,40 | 5,66 | 0,63 | 1,00 | 0,26 | 0,07 | 0,76 | 0,58 | 0,74 | 0,79 | 86,23 |
| 14 | 102020013 | A | G | missense | 0,000003980 | 42/78 | 2822 | I/V | healthy | motor domain | 21,50 | 5,66 | 0,62 | 1,00 | 0,12 | 1,00 | 0,00 | 0,43 | 0,75 | 0,58 | 48,27 |
| 14 | 102020016 | C | T | missense | 0,000003980 | 42/78 | 2823 | R/W | healthy | motor domain | 28,00 | 3,70 | 0,61 | 1,00 | 0,56 | 0,00 | 1,00 | NA | NA | NA | NA |
| 14 | 102022755 | G | A | missense | 0,000003980 | 43/78 | 2838 | V/I | healthy | motor domain | 23,20 | 5,15 | 0,54 | 1,00 | 0,31 | 0,12 | 0,40 | 0,33 | 0,77 | 0,43 | 24,15 |
| 14 | 102022774 | G | A | missense | 0,000003980 | 43/78 | 2844 | R/H | healthy | motor domain | 24,20 | 5,15 | 0,00 | 1,00 | 0,29 | 0,06 | 0,35 | 0,35 | 0,79 | 0,44 | 26,68 |
| 14 | 102022773 | C | T | missense | 0,000023900 | 43/78 | 2844 | R/C | healthy | motor domain | 27,60 | 5,15 | 0,45 | 1,00 | 0,33 | 0,02 | 0,61 | 0,35 | 0,79 | 0,44 | 26,68 |
| 14 | 102022794 | G | A | missense | 0,000003980 | 43/78 | 2851 | D/N | healthy | motor domain | 23,70 | 5,15 | 0,43 | 1,00 | 0,25 | 0,15 | 0,40 | 0,46 | 0,79 | 0,58 | 48,40 |
| 14 | 102022794 | G | C | missense | 0,000099400 | 43/78 | 2851 | D/H | healthy | motor domain | 27,60 | 5,15 | 0,42 | 1,00 | 0,44 | 0,05 | 0,99 | 0,46 | 0,79 | 0,58 | 48,40 |
| 14 | 102022798 | C | T | missense | 0,000011900 | 43/78 | 2852 | T/M | healthy | motor domain | 19,20 | 4,03 | 0,69 | 0,86 | 0,06 | 0,20 | 0,00 | 0,50 | 0,79 | 0,63 | 58,68 |
| 14 | 102022817 | C | G | missense | 0,000003980 | 43/78 | 2858 | F/L | healthy | motor domain | 23,70 | 0,26 | 0,85 | 1,00 | 0,41 | 0,00 | 0,79 | 0,50 | 0,79 | 0,63 | 58,55 |
| 14 | 102022827 | G | A | missense | 0,000015900 | 43/78 | 2862 | D/N | healthy | motor domain | 23,50 | 5,17 | 0,00 | 1,00 | 0,14 | 0,49 | 0,01 | 0,47 | 0,79 | 0,60 | 52,40 |
| 14 | 102022830 | A | G | missense | 0,000003980 | 43/78 | 2863 | R/G | healthy | motor domain | 23,00 | 5,17 | 0,51 | 0,93 | 0,11 | 0,30 | 0,01 | NA | NA | NA | NA |
| 14 | 102022835 | G | C | missense | 0,000003980 | 43/78 | 2864 | E/D | healthy | motor domain | 20,50 | 4,07 | 0,00 | 0,99 | 0,08 | 0,55 | 0,00 | 0,50 | 0,79 | 0,64 | 59,72 |
| 14 | 102022844 | G | A | missense | 0,000011900 | 43/78 | 2867 | M/I | healthy | motor domain | 23,20 | 5,17 | 0,48 | 1,00 | 0,12 | 0,02 | 0,00 | 0,64 | 0,80 | 0,81 | 88,98 |
| 14 | 102022849 | G | A | missense | 0,000015900 | 43/78 | 2869 | R/Q | healthy | motor domain | 26,30 | 5,17 | 0,67 | 1,00 | 0,38 | 0,06 | 0,96 | 0,67 | 0,79 | 0,84 | 91,84 |
| 14 | 102026576 | T | G | missense | 0,000003980 | 44/78 | 2880 | D/E | healthy | motor domain | 19,39 | 0,52 | 0,53 | 1,00 | 0,23 | 0,19 | 0,04 | 0,56 | 0,79 | 0,70 | 72,52 |
| 14 | 102026574 | G | A | missense | 0,000003980 | 44/78 | 2880 | D/N | healthy | motor domain | 23,70 | 5,77 | 0,54 | 1,00 | 0,15 | 1,00 | 0,02 | 0,56 | 0,79 | 0,70 | 72,52 |
| 14 | 102026613 | G | A | missense | 0,000007950 | 44/78 | 2893 | V/I | healthy | motor domain | 23,40 | 5,91 | 0,25 | 1,00 | 0,20 | 0,16 | 0,23 | 0,38 | 0,78 | 0,48 | 31,50 |
| 14 | 102026698 | G | A | missense | 0,000003980 | 44/78 | 2921 | R/K | healthy | motor domain | 32,00 | 5,63 | 0,76 | 1,00 | 0,50 | 0,00 | 0,89 | 0,30 | 0,75 | 0,40 | 19,95 |
| 14 | 102027182 | G | A | missense | 0,000007960 | 45/78 | 2927 | R/H | healthy | motor domain | 34,00 | 5,91 | 0,57 | 1,00 | 0,55 | 0,00 | 0,99 | 0,33 | 0,73 | 0,46 | 28,34 |
| 14 | 102027245 | G | A | missense | 0,000011900 | 45/78 | 2948 | R/H | healthy | motor domain | 32,00 | 5,81 | 0,74 | 1,00 | 0,69 | 0,00 | 0,99 | 0,33 | 0,76 | 0,44 | 25,80 |
| 14 | 102027250 | G | A | missense | 0,000003980 | 45/78 | 2950 | V/I | healthy | motor domain | 26,60 | 5,81 | 0,62 | 1,00 | 0,32 | 0,00 | 0,89 | 0,33 | 0,76 | 0,44 | 25,80 |
| 14 | 102027265 | G | A | missense | 0,000003980 | 45/78 | 2955 | G/S | healthy | motor domain | 32,00 | 5,81 | 0,45 | 1,00 | 0,57 | 0,00 | 0,97 | 0,30 | 0,77 | 0,39 | 18,25 |
| 14 | 102027426 | G | A | missense | 0,000007950 | 46/78 | 2977 | R/Q | healthy | motor domain | 29,20 | 6,00 | 0,37 | 1,00 | 0,46 | 0,02 | 0,64 | 0,29 | 0,79 | 0,36 | 15,71 |
| 14 | 102027447 | G | T | missense | 0,000003980 | 46/78 | 2984 | G/V | healthy | motor domain | 29,70 | 6,06 | 0,59 | 1,00 | 0,62 | 0,00 | 1,00 | 0,38 | 0,79 | 0,48 | 30,99 |
| 14 | 102027473 | A | G | missense | 0,000003980 | 46/78 | 2993 | I/V | healthy | motor domain | 25,70 | 5,70 | 0,35 | 1,00 | 0,25 | 0,03 | 0,58 | 0,57 | 0,76 | 0,75 | 80,91 |
| 14 | 102027496 | A | C | missense | 0,000003980 | 46/78 | 3000 | L/F | healthy | motor domain | 23,40 | -8,74 | 0,34 | 1,00 | 0,36 | 0,02 | 0,91 | 0,43 | 0,76 | 0,56 | 46,07 |
| 14 | 102027500 | T | G | missense | 0,000003980 | 46/78 | 3002 | S/A | healthy | motor domain | 23,60 | 5,70 | 0,36 | 1,00 | 0,18 | 0,07 | 0,04 | 0,43 | 0,77 | 0,56 | 45,21 |
| 14 | 102027537 | A | G | missense | 0,000000000 | 46/78 | 3014 | N/S | study patient | motor domain | 23,50 | 5,67 | 0,26 | 1,00 | 0,28 | 0,11 | 0,42 | 0,29 | 0,77 | 0,37 | 16,31 |
| 14 | 102027641 | A | G | missense | 0,000003980 | 47/78 | 3024 | D/G | healthy | motor domain | 29,40 | 5,87 | 0,45 | 1,00 | 0,72 | 0,00 | 0,91 | 0,33 | 0,76 | 0,44 | 25,03 |
| 14 | 102027647 | A | G | missense | 0,000011900 | 47/78 | 3026 | Y/C | healthy | motor domain | 25,70 | 5,87 | 0,47 | 1,00 | 0,41 | 0,00 | 0,17 | 0,33 | 0,76 | 0,44 | 24,90 |
| 14 | 102027662 | C | T | missense | 0,000000000 | 47/78 | 3031 | T/M | patient | motor domain | 25,70 | 5,87 | 0,39 | 1,00 | 0,50 | 0,00 | 0,89 | 0,35 | 0,77 | 0,46 | 28,51 |
| 14 | 102027665 | A | T | missense | 0,000003980 | 47/78 | 3032 | Q/L | healthy | motor domain | 26,00 | 5,87 | 0,34 | 1,00 | 0,24 | 0,02 | 0,45 | 0,33 | 0,77 | 0,43 | 23,95 |
| 14 | 102027679 | G | T | missense | 0,000003980 | 47/78 | 3037 | A/S | healthy | motor domain | 22,90 | 5,87 | 0,37 | 1,00 | 0,09 | 0,31 | 0,10 | 0,41 | 0,79 | 0,52 | 38,15 |
| 14 | 102027679 | G | A | missense | 0,000007960 | 47/78 | 3037 | A/T | healthy | motor domain | 27,40 | 5,87 | 0,00 | 1,00 | 0,29 | 0,02 | 0,74 | 0,41 | 0,79 | 0,52 | 38,15 |
| 14 | 102027707 | C | T | missense | 0,000003980 | 47/78 | 3046 | S/L | healthy | motor domain | 27,60 | 5,87 | 0,47 | 1,00 | 0,37 | 0,03 | 0,50 | 0,53 | 0,79 | 0,68 | 68,43 |
| 14 | 102027725 | A | G | missense | 0,000051700 | 47/78 | 3052 | K/R | healthy | motor domain | 22,60 | 4,73 | 0,00 | 1,00 | 0,05 | 0,32 | 0,10 | 0,53 | 0,79 | 0,67 | 67,94 |
| 14 | 102027736 | A | G | missense | 0,000003980 | 47/78 | 3056 | S/G | healthy | motor domain | 23,30 | 5,87 | 0,51 | 1,00 | 0,15 | 0,29 | 0,00 | 0,41 | 0,78 | 0,53 | 39,57 |
| 14 | 102027748 | C | T | missense | 0,000007960 | 47/78 | 3060 | R/C | healthy | motor domain | 31,00 | 5,87 | 0,61 | 1,00 | 0,38 | 0,00 | 0,94 | 0,39 | 0,77 | 0,51 | 35,96 |
| 14 | 102027749 | G | A | missense | 0,000019900 | 47/78 | 3060 | R/H | healthy | motor domain | 33,00 | 5,87 | 0,58 | 1,00 | 0,45 | 0,01 | 0,89 | 0,39 | 0,77 | 0,51 | 35,96 |
| 14 | 102027760 | G | A | missense | 0,000003980 | 47/78 | 3064 | V/I | healthy | motor domain | 23,70 | 5,87 | 0,59 | 1,00 | 0,21 | 0,09 | 0,36 | 0,43 | 0,76 | 0,57 | 46,65 |
| 14 | 102027763 | G | A | missense | 0,000015900 | 47/78 | 3065 | V/M | healthy | motor domain | 32,00 | 5,87 | 0,00 | 1,00 | 0,54 | 0,00 | 1,00 | 0,43 | 0,76 | 0,56 | 46,18 |
| 14 | 102027969 | C | G | missense | 0,000007950 | 48/78 | 3099 | T/S | healthy | motor domain | 22,40 | 5,86 | 0,32 | 1,00 | 0,13 | 0,06 | 0,03 | 0,40 | 0,78 | 0,51 | 37,27 |
| 14 | 102027993 | A | G | missense | 0,000003980 | 48/78 | 3107 | K/R | healthy | motor domain | 23,20 | 5,86 | 0,44 | 1,00 | 0,12 | 0,40 | 0,02 | 0,46 | 0,80 | 0,57 | 47,17 |
| 14 | 102028005 | G | C | missense | 0,000003980 | 48/78 | 3111 | S/T | healthy | motor domain | 23,10 | 4,97 | 0,52 | 1,00 | 0,18 | 0,45 | 0,02 | 0,46 | 0,80 | 0,57 | 47,47 |
| 14 | 102028008 | A | G | missense | 0,000000000 | 48/78 | 3112 | K/R | healthy | motor domain | 23,30 | 4,70 | 0,00 | 1,00 | 0,14 | 0,32 | 0,02 | 0,42 | 0,80 | 0,52 | 38,30 |
| 14 | 102028036 | C | G | missense | 0,000003980 | 48/78 | 3121 | I/M | healthy | motor domain | 12,10 | -7,83 | 0,47 | 0,95 | 0,06 | 0,11 | 0,04 | NA | NA | NA | NA |
| 14 | 102028055 | G | C | missense | 0,000000000 | 48/78 | 3128 | V/L | healthy | motor domain | 20,10 | 4,12 | 0,65 | 0,71 | 0,06 | 0,18 | 0,00 | 0,36 | 0,78 | 0,47 | 29,01 |
| 14 | 102028056 | T | C | missense | 0,000007950 | 48/78 | 3128 | V/A | healthy | motor domain | 22,30 | 5,96 | 0,70 | 0,57 | 0,10 | 0,23 | 0,00 | 0,36 | 0,78 | 0,47 | 29,01 |
| 14 | 102028092 | G | A | missense | 0,000003980 | 48/78 | 3140 | R/Q | healthy | motor domain | 33,00 | 5,96 | 0,47 | 1,00 | 0,40 | 0,02 | 0,87 | 0,30 | 0,76 | 0,39 | 19,00 |
| 14 | 102029561 | G | A | missense | 0,000011900 | 49/78 | 3164 | R/Q | healthy | motor domain | 25,70 | 5,37 | 0,41 | 1,00 | 0,30 | 0,46 | 0,87 | 0,27 | 0,76 | 0,36 | 15,47 |
| 14 | 102029566 | G | A | missense | 0,000003980 | 49/78 | 3166 | G/S | healthy | motor domain | 23,80 | 5,37 | 0,33 | 1,00 | 0,28 | 0,09 | 0,30 | 0,25 | 0,76 | 0,33 | 13,23 |
| 14 | 102029572 | A | G | missense | 0,000003980 | 49/78 | 3168 | T/A | healthy | motor domain | 23,20 | 5,37 | 0,33 | 1,00 | 0,38 | 0,46 | 0,44 | 0,25 | 0,76 | 0,33 | 13,23 |
| 14 | 102029588 | C | G | missense | 0,000000000 | 49/78 | 3173 | P/R | study patient | motor domain | 26,40 | 5,37 | 0,63 | 1,00 | 0,69 | 0,00 | 1,00 | 0,33 | 0,76 | 0,44 | 25,46 |
| 14 | 102029624 | A | C | missense | 0,000003980 | 49/78 | 3185 | N/T | healthy | motor domain | 24,00 | 5,37 | 0,65 | 1,00 | 0,19 | 0,09 | 0,17 | 0,38 | 0,78 | 0,48 | 30,99 |
| 14 | 102029635 | G | A | missense | 0,000023900 | 49/78 | 3189 | E/K | healthy | motor domain | 29,10 | 5,37 | 0,36 | 1,00 | 0,51 | 0,07 | 0,98 | 0,30 | 0,80 | 0,38 | 16,83 |
| 14 | 102029642 | G | A | missense | 0,000003980 | 49/78 | 3191 | R/Q | healthy | stalk/MTBD | 34,00 | 5,37 | 0,62 | 1,00 | 0,48 | 0,00 | 0,94 | 0,30 | 0,79 | 0,38 | 16,98 |
| 14 | 102029848 | A | G | missense | 0,000003980 | 50/78 | 3224 | I/M | healthy | stalk/MTBD | 16,77 | -3,24 | 0,46 | 1,00 | 0,21 | 0,10 | 0,05 | 0,20 | 0,78 | 0,26 | 6,50 |
| 14 | 102029860 | G | C | missense | 0,000000000 | 50/78 | 3228 | E/D | patient | stalk/MTBD | 25,30 | 5,54 | 0,43 | 1,00 | 0,50 | 0,11 | 0,80 | 0,25 | 0,79 | 0,32 | 11,51 |
| 14 | 102029913 | A | G | missense | 0,000003980 | 50/78 | 3246 | D/G | healthy | stalk/MTBD | 25,20 | 4,49 | 0,36 | 1,00 | 0,57 | 0,01 | 0,33 | 0,20 | 0,82 | 0,24 | 5,44 |
| 14 | 102030196 | A | T | missense | 0,000015900 | 51/78 | 3266 | K/M | healthy | stalk/MTBD | 25,60 | 5,63 | 0,39 | 1,00 | 0,38 | 0,03 | 0,03 | 0,22 | 0,83 | 0,27 | 7,68 |
| 14 | 102030228 | A | C | missense | 0,000007950 | 51/78 | 3277 | S/R | healthy | stalk/MTBD | 22,70 | 5,63 | 0,35 | 1,00 | 0,25 | 0,59 | 0,00 | 0,27 | 0,81 | 0,34 | 13, Jun |
| 14 | 102030268 | T | C | missense | 0,000003980 | 51/78 | 3290 | I/T | healthy | stalk/MTBD | 23,40 | 5,63 | 0,54 | 1,00 | 0,41 | 0,49 | 0,27 | 0,20 | 0,79 | 0,25 | 6,28 |
| 14 | 102032332 | C | G | missense | 0,000007950 | 52/78 | 3315 | A/G | healthy | stalk/MTBD | 21,80 | 5,60 | 0,58 | 1,00 | 0,19 | 0,24 | 0,01 | 0,17 | 0,74 | 0,23 | 4,71 |
| 14 | 102032347 | C | T | missense | 0,000000000 | 52/78 | 3320 | A/V | patient | stalk/MTBD | 27,20 | 5,60 | 0,78 | 1,00 | 0,52 | 0,00 | 0,89 | 0,17 | 0,74 | 0,23 | 24563,00 |
| 14 | 102032396 | G | T | missense | 0,000000000 | 52/78 | 3336 | K/N | patient | stalk/MTBD | 26,00 | 4,60 | 0,45 | 1,00 | 0,54 | 0,04 | 0,98 | 0,25 | 0,75 | 0,34 | 13,43 |
| 14 | 102032415 | A | C | missense | 0,000003980 | 52/78 | 3343 | M/L | healthy | stalk/MTBD | 23,00 | 5,36 | 0,65 | 1,00 | 0,34 | 0,29 | 0,01 | 0,25 | 0,77 | 0,33 | 12,35 |
| 14 | 102032419 | G | A | missense | 0,000000000 | 52/78 | 3344 | R/Q | patient | stalk/MTBD | 25,00 | 5,36 | 0,40 | 1,00 | 0,44 | 0,06 | 0,11 | 0,25 | 0,77 | 0,32 | 12,22 |
| 14 | 102032421 | G | A | missense | 0,000000000 | 52/78 | 3345 | E/K | patient | stalk/MTBD | 26,70 | 5,36 | 0,67 | 1,00 | 0,48 | 0,02 | 0,08 | 0,25 | 0,77 | 0,32 | 12,01 |
| 14 | 102032439 | A | T | missense | 0,000000000 | 52/78 | 3351 | I/F | patient | stalk/MTBD | 27,90 | 5,36 | 0,65 | 1,00 | 0,91 | 0,00 | 0,96 | 0,57 | 0,81 | 0,71 | 73,92 |
| 14 | 102033068 | C | A | missense | 0,000003980 | 53/78 | 3361 | D/E | healthy | stalk/MTBD | 6,74 | -5,17 | 0,36 | 1,00 | 0,21 | 0,80 | 0,03 | 0,63 | 0,82 | 0,77 | 83,22 |
| 14 | 102033069 | G | T | missense | 0,000003980 | 53/78 | 3362 | A/S | healthy | stalk/MTBD | 21,90 | 5,36 | 0,00 | 1,00 | 0,28 | 1,00 | 0,00 | 0,63 | 0,81 | 0,77 | 83,69 |
| 14 | 102033069 | G | A | missense | 0,000003980 | 53/78 | 3362 | A/T | healthy | stalk/MTBD | 23,10 | 5,36 | 0,50 | 1,00 | 0,25 | 0,28 | 0,01 | 0,63 | 0,81 | 0,77 | 83,69 |
| 14 | 102033088 | A | G | missense | 0,000003980 | 53/78 | 3368 | K/R | healthy | stalk/MTBD | 23,30 | 5,36 | 0,41 | 1,00 | 0,24 | 0,71 | 0,05 | 0,67 | 0,79 | 0,85 | 92,00 |
| 14 | 102033099 | A | T | missense | 0,000003980 | 53/78 | 3372 | M/L | healthy | stalk/MTBD | 21,40 | 5,36 | 0,68 | 0,79 | 0,13 | 1,00 | 0,00 | 0,86 | 0,80 | 1,08 | 99,51 |
| 14 | 102033128 | T | G | missense | 0,000003980 | 53/78 | 3381 | I/M | healthy | stalk/MTBD | 17,79 | -1,73 | 0,66 | 0,91 | 0,14 | 0,03 | 0,08 | NA | NA | NA | NA |
| 14 | 102033136 | G | A | missense | 0,000000000 | 53/78 | 3384 | R/Q | patient | stalk/MTBD | 33,00 | 4,56 | 0,58 | 1,00 | 0,40 | 0,01 | 0,82 | 0,60 | 0,80 | 0,76 | 81,54 |
| 14 | 102033145 | T | C | missense | 0,000000000 | 53/78 | 3387 | L/P | patient | stalk/MTBD | 29,90 | 5,45 | 0,66 | 1,00 | 0,33 | 0,04 | 0,52 | 0,43 | 0,80 | 0,54 | 40,99 |
| 14 | 102033157 | C | T | missense | 0,000000000 | 53/78 | 3391 | P/L | patient | stalk/MTBD | 27,50 | 5,45 | 0,59 | 1,00 | 0,67 | 0,00 | 1,00 | 0,22 | 0,78 | 0,29 | 9,15 |
| 14 | 102033174 | A | G | missense | 0,000007950 | 53/78 | 3397 | I/V | healthy | stalk/MTBD | 19,55 | 3,10 | 0,00 | 1,00 | 0,18 | 0,29 | 0,05 | 0,10 | 0,77 | 0,13 | 0,90 |
| 14 | 102033309 | G | A | missense | 0,000000000 | 54/78 | 3413 | R/H | healthy | stalk/MTBD | 34,00 | 5,77 | 0,43 | 1,00 | 0,75 | 0,00 | 0,99 | 0,15 | 0,79 | 0,20 | 3,27 |
| 14 | 102033333 | A | G | missense | 0,000027800 | 54/78 | 3421 | D/G | healthy | stalk/MTBD | 24,40 | 5,77 | 0,49 | 1,00 | 0,35 | 0,07 | 0,07 | 0,25 | 0,80 | 0,31 | 11,43 |
| 14 | 102033351 | A | C | missense | 0,000000000 | 54/78 | 3427 | Q/P | patient | stalk/MTBD | 25,40 | 5,77 | 0,44 | 1,00 | 0,46 | 0,01 | 0,12 | 0,42 | 0,78 | 0,53 | 39,90 |
| 14 | 102033363 | A | G | missense | 0,000007950 | 54/78 | 3431 | N/S | healthy | stalk/MTBD | 23,20 | 5,77 | 0,38 | 1,00 | 0,15 | 0,45 | 0,01 | 0,43 | 0,79 | 0,54 | 41,73 |
| 14 | 102033384 | G | A | missense | 0,000007960 | 54/78 | 3438 | R/Q | healthy | stalk/MTBD | 23,90 | 5,77 | 0,34 | 1,00 | 0,29 | 0,55 | 0,01 | 0,43 | 0,78 | 0,55 | 42,59 |
| 14 | 102033386 | G | T | missense | 0,000003980 | 54/78 | 3439 | D/Y | healthy | stalk/MTBD | 26,30 | 5,77 | 0,38 | 1,00 | 0,61 | 0,00 | 0,42 | 0,43 | 0,78 | 0,55 | 42,59 |
| 14 | 102033404 | G | A | missense | 0,000007960 | 54/78 | 3445 | A/T | healthy | stalk/MTBD | 24,30 | 5,86 | 0,44 | 1,00 | 0,43 | 0,09 | 0,14 | 0,46 | 0,78 | 0,59 | 51,97 |
| 14 | 102033408 | G | A | missense | 0,000003980 | 54/78 | 3446 | R/H | healthy | stalk/MTBD | 27,90 | 5,86 | 0,44 | 1,00 | 0,61 | 0,07 | 0,68 | 0,46 | 0,77 | 0,60 | 52,92 |
| 14 | 102033419 | G | A | missense | 0,000000000 | 54/78 | 3450 | E/K | patient | stalk/MTBD | 34,00 | 5,86 | 0,42 | 1,00 | 0,69 | 0,00 | 1,00 | 0,46 | 0,76 | 0,60 | 53,60 |
| 14 | 102033428 | G | A | missense | 0,000019900 | 54/78 | 3453 | V/I | healthy | stalk/MTBD | 23,10 | 5,86 | 0,57 | 1,00 | 0,25 | 0,11 | 0,27 | 0,39 | 0,76 | 0,51 | 36,54 |
| 14 | 102033982 | C | T | missense | 0,000000000 | 55/78 | 3474 | R/W | patient | stalk/MTBD | 25,50 | 3,77 | 0,47 | 1,00 | 0,74 | 0,00 | 1,00 | NA | NA | NA | NA |
| 14 | 102033994 | C | T | missense | 0,000000000 | 55/78 | 3478 | L/F | study patient | stalk/MTBD | 25,40 | 5,72 | 0,43 | 1,00 | 0,60 | 0,00 | 1,00 | 0,21 | 0,76 | 0,28 | 09, Jun |
| 14 | 102034018 | C | T | missense | 0,000003980 | 55/78 | 3486 | R/C | healthy | stalk/MTBD | 28,30 | 4,81 | 0,53 | 1,00 | 0,67 | 0,00 | 0,99 | 0,23 | 0,76 | 0,30 | 10,35 |
| 14 | 102034019 | G | A | missense | 0,000007960 | 55/78 | 3486 | R/H | healthy | stalk/MTBD | 33,00 | 4,83 | 0,55 | 1,00 | 0,60 | 0,00 | 0,99 | 0,23 | 0,76 | 0,30 | 10,35 |
| 14 | 102034035 | A | C | missense | 0,000003980 | 55/78 | 3491 | K/N | healthy | stalk/MTBD | 22,10 | -0,57 | 0,38 | 1,00 | 0,12 | 0,28 | 0,17 | 0,29 | 0,76 | 0,37 | 16,61 |
| 14 | 102034073 | C | T | missense | 0,000003980 | 55/78 | 3504 | A/V | healthy | motor domain | 22,70 | 5,32 | 0,33 | 1,00 | 0,39 | 1,00 | 0,01 | 0,31 | 0,79 | 0,39 | 18,42 |
| 14 | 102034084 | C | A | missense | 0,000000000 | 55/78 | 3508 | L/I | healthy | motor domain | 26,00 | 5,32 | 0,00 | 1,00 | 0,55 | 0,02 | 0,98 | 0,33 | 0,79 | 0,42 | 22,27 |
| 14 | 102034112 | C | T | missense | 0,000007960 | 55/78 | 3517 | A/V | healthy | motor domain | 26,10 | 4,15 | 0,57 | 1,00 | 0,43 | 0,01 | 0,77 | 0,43 | 0,79 | 0,55 | 42,29 |
| 14 | 102034129 | C | G | missense | 0,000003980 | 55/78 | 3523 | Q/E | healthy | motor domain | 23,90 | 4,94 | 0,56 | 1,00 | 0,24 | 0,04 | 0,05 | 0,50 | 0,79 | 0,64 | 59,50 |
| 14 | 102034135 | C | T | missense | 0,000000000 | 55/78 | 3525 | R/C | patient | motor domain | 32,00 | 4,05 | 0,72 | 1,00 | 0,88 | 0,00 | 1,00 | 0,50 | 0,78 | 0,64 | 60,17 |
| 14 | 102034145 | T | C | missense | 0,000003980 | 55/78 | 3528 | L/S | healthy | motor domain | 33,00 | 4,94 | 0,79 | 1,00 | 0,95 | 0,00 | 1,00 | 0,60 | 0,78 | 0,77 | 83,58 |
| 14 | 102034153 | A | G | missense | 0,000003980 | 55/78 | 3531 | T/A | healthy | motor domain | 23,00 | 3,79 | 0,78 | 1,00 | 0,12 | 0,27 | 0,04 | 0,64 | 0,79 | 0,81 | 88,75 |
| 14 | 102034172 | A | G | missense | 0,000003980 | 55/78 | 3537 | Q/R | healthy | motor domain | 23,50 | 4,81 | 0,55 | 1,00 | 0,28 | 0,35 | 0,16 | 0,73 | 0,79 | 0,92 | 95,83 |
| 14 | 102034327 | C | G | missense | 0,000003980 | 56/78 | 3543 | F/L | healthy | motor domain | 24,10 | 5,07 | 0,61 | 1,00 | 0,24 | 0,06 | 0,44 | 0,83 | 0,79 | 1,06 | 99,40 |
| 14 | 102034329 | G | A | missense | 0,000007950 | 56/78 | 3544 | R/H | healthy | motor domain | 34,00 | 5,07 | 0,53 | 1,00 | 0,56 | 0,00 | 0,98 | 0,83 | 0,78 | 1,06 | 99,42 |
| 14 | 102034338 | T | C | missense | 0,000003980 | 56/78 | 3547 | I/T | healthy | motor domain | 24,30 | 5,17 | 0,63 | 1,00 | 0,47 | 0,05 | 0,28 | 0,75 | 0,78 | 0,97 | 97,38 |
| 14 | 102034347 | C | T | missense | 0,000003980 | 56/78 | 3550 | T/M | healthy | motor domain | 26,70 | 4,28 | 0,59 | 1,00 | 0,42 | 0,02 | 0,65 | 0,79 | 0,77 | 1,02 | 98,73 |
| 14 | 102034364 | G | C | missense | 0,000003980 | 56/78 | 3556 | A/P | healthy | motor domain | 22,90 | 4,27 | 0,62 | 1,00 | 0,30 | 0,46 | 0,01 | 0,67 | 0,77 | 0,86 | 93,33 |
| 14 | 102034364 | G | A | missense | 0,000003980 | 56/78 | 3556 | A/T | healthy | motor domain | 23,60 | 4,27 | 0,60 | 1,00 | 0,24 | 0,07 | 0,42 | 0,67 | 0,77 | 0,86 | 93,33 |
| 14 | 102034369 | T | A | missense | 0,000003980 | 56/78 | 3557 | D/E | healthy | motor domain | 16,83 | -2,43 | 0,56 | 1,00 | 0,19 | 0,08 | 0,37 | 0,67 | 0,78 | 0,86 | 93,05 |
| 14 | 102034374 | G | A | missense | 0,000007950 | 56/78 | 3559 | R/H | healthy | motor domain | 29,90 | 4,27 | 0,54 | 1,00 | 0,55 | 0,00 | 1,00 | 0,64 | 0,78 | 0,83 | 90,70 |
| 14 | 102034385 | C | G | missense | 0,000003980 | 56/78 | 3563 | Q/E | healthy | motor domain | 24,10 | 4,27 | 0,00 | 1,00 | 0,22 | 0,00 | 0,34 | 0,58 | 0,77 | 0,76 | 82,35 |
| 14 | 102034392 | G | A | missense | 0,000039800 | 56/78 | 3565 | S/N | healthy | motor domain | 22,70 | 4,24 | 0,48 | 1,00 | 0,09 | 1,00 | 0,00 | 0,62 | 0,77 | 0,80 | 87,56 |
| 14 | 102034436 | C | G | missense | 0,000031800 | 56/78 | 3580 | L/V | healthy | motor domain | 26,80 | 5,17 | 0,00 | 1,00 | 0,27 | 0,00 | 0,98 | NA | NA | NA | NA |
| 14 | 102034441 | A | C | missense | 0,000007950 | 56/78 | 3581 | K/N | healthy | motor domain | 24,80 | -8,96 | 0,00 | 1,00 | 0,33 | 0,05 | 0,80 | NA | NA | NA | NA |
| 14 | 102034448 | A | G | missense | 0,000003980 | 56/78 | 3584 | N/D | healthy | motor domain | 25,50 | 5,17 | 0,49 | 1,00 | 0,34 | 0,05 | 0,92 | 0,44 | 0,78 | 0,57 | 47,97 |
| 14 | 102034449 | A | G | missense | 0,000003980 | 56/78 | 3584 | N/S | healthy | motor domain | 26,60 | 5,17 | 0,46 | 1,00 | 0,36 | 0,04 | 0,85 | 0,44 | 0,78 | 0,57 | 47,97 |
| 14 | 102036547 | A | G | missense | 0,000000000 | 57/78 | 3605 | K/E | healthy | motor domain | 24,30 | 5,83 | 0,66 | 1,00 | 0,23 | 0,07 | 0,05 | 0,56 | 0,79 | 0,71 | 73,38 |
| 14 | 102036554 | G | A | missense | 0,000000000 | 57/78 | 3607 | R/H | healthy | motor domain | 26,60 | 4,94 | 0,57 | 1,00 | 0,32 | 0,00 | 0,01 | 0,60 | 0,78 | 0,77 | 84,44 |
| 14 | 102036561 | C | G | missense | 0,000003980 | 57/78 | 3609 | I/M | healthy | motor domain | 23,00 | 2,53 | 0,79 | 1,00 | 0,26 | 0,09 | 0,68 | NA | NA | NA | NA |
| 14 | 102036566 | G | A | missense | 0,000007950 | 57/78 | 3611 | R/Q | healthy | motor domain | 24,70 | 5,83 | 0,73 | 1,00 | 0,30 | 0,32 | 0,03 | 0,58 | 0,79 | 0,74 | 79,79 |
| 14 | 102036569 | C | T | missense | 0,000003980 | 57/78 | 3612 | T/I | healthy | motor domain | 27,50 | 5,83 | 0,55 | 1,00 | 0,52 | 0,00 | 1,00 | 0,58 | 0,78 | 0,75 | 80,35 |
| 14 | 102036596 | A | G | missense | 0,000000000 | 57/78 | 3621 | K/R | healthy | motor domain | 31,00 | 4,67 | 0,63 | 1,00 | 0,42 | 0,00 | 1,00 | 0,46 | 0,79 | 0,59 | 50,94 |
| 14 | 102036610 | G | T | missense | 0,000003980 | 57/78 | 3626 | A/S | healthy | motor domain | 23,80 | 5,83 | 0,67 | 1,00 | 0,28 | 0,19 | 0,30 | 0,42 | 0,77 | 0,54 | 41,58 |
| 14 | 102038463 | G | A | missense | 0,000003980 | 58/78 | 3638 | V/M | healthy | motor domain | 31,00 | 4,94 | 0,41 | 1,00 | 0,41 | 0,00 | 1,00 | 0,40 | 0,74 | 0,54 | 41,21 |
| 14 | 102038484 | T | A | missense | 0,000003980 | 58/78 | 3645 | L/M | healthy | motor domain | 22,90 | -2,77 | 0,64 | 1,00 | 0,47 | 0,00 | 1,00 | 0,54 | 0,72 | 0,75 | 80,35 |
| 14 | 102038503 | G | A | missense | 0,000011900 | 58/78 | 3651 | R/H | healthy | motor domain | 27,10 | 5,83 | 0,68 | 1,00 | 0,43 | 0,03 | 0,21 | 0,53 | 0,73 | 0,73 | 77,99 |
| 14 | 102038512 | G | A | missense | 0,000003980 | 58/78 | 3654 | R/Q | healthy | motor domain | 25,90 | 5,83 | 0,70 | 1,00 | 0,31 | 0,55 | 0,48 | 0,47 | 0,72 | 0,65 | 61,98 |
| 14 | 102038521 | G | T | missense | 0,000007960 | 58/78 | 3657 | G/V | healthy | motor domain | 28,60 | 5,83 | 0,82 | 1,00 | 0,42 | 0,01 | 0,59 | 0,44 | 0,73 | 0,60 | 52,92 |
| 14 | 102038521 | G | C | missense | 0,000019900 | 58/78 | 3657 | G/A | healthy | motor domain | 32,00 | 5,83 | 0,81 | 1,00 | 0,60 | 0,00 | 0,98 | 0,44 | 0,73 | 0,60 | 52,92 |
| 14 | 102038529 | G | A | missense | 0,000003980 | 58/78 | 3660 | V/M | healthy | motor domain | 32,00 | 5,83 | 0,74 | 1,00 | 0,32 | 0,00 | 0,99 | 0,42 | 0,72 | 0,58 | 50,03 |
| 14 | 102038538 | A | G | missense | 0,000007960 | 58/78 | 3663 | T/A | healthy | motor domain | 24,50 | 5,83 | 0,45 | 1,00 | 0,35 | 0,05 | 0,34 | 0,44 | 0,71 | 0,63 | 57,35 |
| 14 | 102038572 | C | T | missense | 0,000007960 | 58/78 | 3674 | S/L | healthy | motor domain | 25,50 | 4,94 | 0,42 | 1,00 | 0,37 | 0,00 | 0,71 | NA | NA | NA | NA |
| 14 | 102038577 | G | A | missense | 0,000003980 | 58/78 | 3676 | V/I | healthy | motor domain | 23,60 | 4,94 | 0,34 | 1,00 | 0,29 | 0,17 | 0,17 | 0,31 | 0,72 | 0,44 | 24,49 |
| 14 | 102038595 | C | T | missense | 0,000007960 | 58/78 | 3682 | R/W | healthy | motor domain | 31,00 | 3,82 | 0,49 | 1,00 | 0,47 | 0,00 | 1,00 | NA | NA | NA | NA |
| 14 | 102038701 | G | A | missense | 0,000003980 | 59/78 | 3687 | E/K | healthy | motor domain | 26,90 | 5,51 | 0,46 | 1,00 | 0,34 | 0,12 | 0,75 | 0,33 | 0,74 | 0,45 | 27,48 |
| 14 | 102038756 | G | A | missense | 0,000003980 | 59/78 | 3705 | R/H | healthy | motor domain | 26,20 | 5,83 | 0,35 | 1,00 | 0,31 | 0,05 | 0,18 | 0,38 | 0,76 | 0,49 | 33,31 |
| 14 | 102038755 | C | T | missense | 0,000003980 | 59/78 | 3705 | R/C | healthy | motor domain | 27,60 | 3,97 | 0,38 | 1,00 | 0,28 | 0,00 | 0,97 | 0,38 | 0,76 | 0,49 | 33,31 |
| 14 | 102038775 | G | T | missense | 0,000003980 | 59/78 | 3711 | Q/H | healthy | motor domain | 27,70 | 4,93 | 0,33 | 1,00 | 0,38 | 0,01 | 1,00 | 0,36 | 0,76 | 0,48 | 30,64 |
| 14 | 102038812 | G | A | missense | 0,000003980 | 59/78 | 3724 | V/M | healthy | motor domain | 32,00 | 5,69 | 0,51 | 1,00 | 0,38 | 0,00 | 0,97 | 0,38 | 0,77 | 0,49 | 32,32 |
| 14 | 102038825 | G | C | missense | 0,000000000 | 59/78 | 3728 | R/P | patient | motor domain | 34,00 | 5,69 | 0,44 | 1,00 | 0,61 | 0,00 | 1,00 | 0,43 | 0,77 | 0,56 | 45,02 |
| 14 | 102038847 | A | C | missense | 0,000003980 | 59/78 | 3735 | Q/H | healthy | motor domain | 28,10 | 4,45 | 0,37 | 1,00 | 0,36 | 0,00 | 1,00 | 0,50 | 0,75 | 0,67 | 66,86 |
| 14 | 102039022 | G | A | missense | 0,000003980 | 60/78 | 3743 | R/H | healthy | motor domain | 34,00 | 4,80 | 0,49 | 1,00 | 0,53 | 0,04 | 1,00 | 0,50 | 0,75 | 0,66 | 65,85 |
| 14 | 102039046 | A | T | missense | 0,000007960 | 60/78 | 3751 | Q/L | healthy | motor domain | 29,40 | 4,54 | 0,43 | 1,00 | 0,41 | 0,01 | 0,56 | 0,38 | 0,74 | 0,50 | 35,44 |
| 14 | 102039088 | C | T | missense | 0,000003980 | 60/78 | 3765 | T/M | healthy | motor domain | 26,60 | 4,81 | 0,29 | 1,00 | 0,27 | 0,02 | 0,76 | 0,20 | 0,74 | 0,27 | 8,03 |
| 14 | 102039144 | G | A | missense | 0,000003980 | 60/78 | 3784 | V/I | healthy | motor domain | 27,30 | 5,93 | 0,36 | 1,00 | 0,31 | 0,04 | 0,73 | 0,50 | 0,76 | 0,66 | 65,07 |
| 14 | 102039147 | G | A | missense | 0,000003980 | 60/78 | 3785 | E/K | healthy | motor domain | 24,20 | 6,07 | 0,42 | 1,00 | 0,32 | 0,20 | 0,11 | 0,50 | 0,75 | 0,66 | 65,85 |
| 14 | 102039159 | A | G | missense | 0,000003980 | 60/78 | 3789 | I/V | healthy | motor domain | 17,73 | -0,59 | 0,25 | 1,00 | 0,14 | 0,32 | 0,00 | 0,58 | 0,76 | 0,76 | 82,89 |
| 14 | 102039160 | T | C | missense | 0,000000000 | 60/78 | 3789 | I/T | healthy | motor domain | 22,50 | 6,07 | 0,00 | 1,00 | 0,10 | 0,60 | 0,00 | 0,58 | 0,76 | 0,76 | 82,89 |
| 14 | 102039168 | C | A | missense | 0,000015900 | 60/78 | 3792 | Q/K | healthy | motor domain | 22,10 | 5,07 | 0,43 | 1,00 | 0,06 | 0,37 | 0,00 | 0,58 | 0,77 | 0,75 | 81,13 |
| 14 | 102039179 | G | T | missense | 0,000015900 | 60/78 | 3795 | E/D | healthy | motor domain | 23,30 | 4,98 | 0,36 | 1,00 | 0,18 | 0,19 | 0,11 | 0,55 | 0,78 | 0,70 | 72,65 |
| 14 | 102039183 | G | A | missense | 0,000003980 | 60/78 | 3797 | V/M | healthy | motor domain | 32,00 | 5,18 | 0,41 | 1,00 | 0,41 | 0,00 | 1,00 | 0,55 | 0,77 | 0,71 | 73,06 |
| 14 | 102039193 | A | G | missense | 0,000003980 | 60/78 | 3800 | Q/R | healthy | motor domain | 24,50 | 6,07 | 0,37 | 1,00 | 0,17 | 0,05 | 0,19 | 0,50 | 0,78 | 0,64 | 61,09 |
| 14 | 102039202 | C | T | missense | 0,000003980 | 60/78 | 3803 | P/L | healthy | motor domain | 23,00 | 5,18 | 0,44 | 1,00 | 0,34 | 0,22 | 0,01 | 0,53 | 0,79 | 0,67 | 66,50 |
| 14 | 102039202 | C | A | missense | 0,000011900 | 60/78 | 3803 | P/Q | healthy | motor domain | 23,30 | 5,18 | 0,48 | 1,00 | 0,31 | 0,17 | 0,20 | 0,53 | 0,79 | 0,67 | 66,50 |
| 14 | 102039202 | C | G | missense | 0,000011900 | 60/78 | 3803 | P/R | healthy | motor domain | 24,40 | 5,18 | 0,45 | 1,00 | 0,36 | 0,09 | 0,56 | 0,53 | 0,79 | 0,67 | 66,50 |
| 14 | 102039219 | A | C | missense | 0,000003980 | 60/78 | 3809 | S/R | healthy | motor domain | 32,00 | 5,88 | 0,50 | 1,00 | 0,82 | 0,00 | 1,00 | 0,48 | 0,79 | 0,61 | 54,27 |
| 14 | 102039251 | G | C | missense | 0,000003990 | 60/78 | 3819 | K/N | healthy | motor domain | 17,24 | 2,64 | 0,46 | 1,00 | 0,10 | 1,00 | 0,00 | 0,39 | 0,80 | 0,48 | 31,70 |
| 14 | 102039412 | A | G | missense | 0,000003980 | 61/78 | 3821 | I/V | healthy | motor domain | 22,90 | 4,68 | 0,41 | 1,00 | 0,09 | 1,00 | 0,00 | 0,43 | 0,79 | 0,54 | 41,94 |
| 14 | 102039417 | C | G | missense | 0,000000000 | 61/78 | 3822 | H/Q | healthy | motor domain | 25,70 | 5,82 | 0,42 | 1,00 | 0,34 | 0,00 | 0,99 | 0,43 | 0,79 | 0,54 | 41,94 |
| 14 | 102039416 | A | C | missense | 0,000000000 | 61/78 | 3822 | H/P | patient | motor domain | 31,00 | 5,82 | 0,56 | 1,00 | 0,72 | 0,00 | 0,99 | 0,43 | 0,79 | 0,54 | 41,94 |
| 14 | 102039424 | T | C | missense | 0,000003980 | 61/78 | 3825 | Y/H | healthy | motor domain | 33,00 | 5,82 | 0,84 | 1,00 | 0,91 | 0,00 | 1,00 | 0,39 | 0,80 | 0,48 | 31,35 |
| 14 | 102039454 | A | G | missense | 0,000007950 | 61/78 | 3835 | I/V | healthy | motor domain | 24,20 | 5,82 | 0,00 | 1,00 | 0,25 | 0,04 | 0,02 | 0,42 | 0,79 | 0,53 | 40,13 |
| 14 | 102039482 | C | T | missense | 0,000023900 | 61/78 | 3844 | P/L | healthy | motor domain | 23,50 | 5,59 | 0,38 | 1,00 | 0,33 | 0,01 | 0,07 | 0,50 | 0,77 | 0,65 | 62,77 |
| 14 | 102039493 | G | A | missense | 0,000003980 | 61/78 | 3848 | G/S | healthy | motor domain | 20,80 | 4,68 | 0,29 | 1,00 | 0,10 | 0,28 | 0,00 | 0,50 | 0,77 | 0,65 | 63,87 |
| 14 | 102039494 | G | T | missense | 0,000003980 | 61/78 | 3848 | G/V | healthy | motor domain | 22,20 | 5,59 | 0,38 | 1,00 | 0,18 | 0,16 | 0,00 | 0,50 | 0,77 | 0,65 | 63,87 |
| 14 | 102039494 | G | A | missense | 0,000011900 | 61/78 | 3848 | G/D | healthy | motor domain | 23,70 | 5,59 | 0,41 | 1,00 | 0,28 | 0,01 | 0,19 | 0,50 | 0,77 | 0,65 | 63,87 |
| 14 | 102039502 | G | A | missense | 0,000003980 | 61/78 | 3851 | D/N | healthy | motor domain | 23,50 | 5,59 | 0,41 | 1,00 | 0,39 | 0,16 | 0,22 | 0,50 | 0,76 | 0,66 | 64,24 |
| 14 | 102039508 | A | G | missense | 0,000003980 | 61/78 | 3853 | T/A | healthy | motor domain | 21,60 | 5,59 | 0,28 | 1,00 | 0,09 | 0,93 | 0,00 | 0,52 | 0,75 | 0,70 | 71,57 |
| 14 | 102039512 | A | C | missense | 0,000003980 | 61/78 | 3854 | Q/P | healthy | motor domain | 23,00 | 5,59 | 0,43 | 1,00 | 0,22 | 0,21 | 0,00 | 0,54 | 0,75 | 0,72 | 76,26 |
| 14 | 102039515 | G | A | missense | 0,000003980 | 61/78 | 3855 | R/H | healthy | motor domain | 33,00 | 5,42 | 0,61 | 1,00 | 0,78 | 0,00 | 1,00 | 0,54 | 0,75 | 0,72 | 75,43 |
| 14 | 102039523 | A | G | missense | 0,000007950 | 61/78 | 3858 | I/V | healthy | motor domain | 14,08 | 0,23 | 0,44 | 0,72 | 0,04 | 0,25 | 0,00 | 0,60 | 0,75 | 0,80 | 87,86 |
| 14 | 102039526 | A | G | missense | 0,000003980 | 61/78 | 3859 | I/V | healthy | motor domain | 22,00 | 3,06 | 0,61 | 1,00 | 0,11 | 0,10 | 0,02 | 0,60 | 0,75 | 0,80 | 87,86 |
| 14 | 102039532 | A | C | missense | 0,000003980 | 61/78 | 3861 | K/Q | healthy | motor domain | 23,80 | 5,42 | 0,45 | 1,00 | 0,18 | 0,21 | 0,05 | 0,64 | 0,76 | 0,84 | 91,35 |
| 14 | 102039532 | A | G | missense | 0,000003980 | 61/78 | 3861 | K/E | healthy | motor domain | 24,00 | 5,42 | 0,00 | 1,00 | 0,20 | 0,18 | 0,01 | 0,64 | 0,76 | 0,84 | 91,35 |
| 14 | 102039642 | C | T | missense | 0,000003980 | 62/78 | 3867 | A/V | healthy | motor domain | 18,66 | 4,58 | 0,00 | 1,00 | 0,12 | 0,93 | 0,00 | 0,59 | 0,75 | 0,78 | 85,43 |
| 14 | 102039660 | G | C | missense | 0,000011900 | 62/78 | 3873 | R/P | healthy | motor domain | 24,00 | 5,50 | 0,54 | 1,00 | 0,64 | 0,22 | 0,23 | 0,54 | 0,75 | 0,72 | 76,26 |
| 14 | 102039660 | G | A | missense | 0,000031800 | 62/78 | 3873 | R/Q | healthy | motor domain | 24,00 | 5,50 | 0,59 | 1,00 | 0,65 | 0,20 | 0,04 | 0,54 | 0,75 | 0,72 | 76,26 |
| 14 | 102039713 | A | G | missense | 0,000003980 | 62/78 | 3891 | K/E | healthy | motor domain | 23,00 | 5,75 | 0,47 | 1,00 | 0,17 | 0,22 | 0,00 | 0,46 | 0,76 | 0,60 | 53,60 |
| 14 | 102039722 | G | C | missense | 0,000003980 | 62/78 | 3894 | G/R | healthy | motor domain | 25,20 | 5,75 | 0,57 | 1,00 | 0,44 | 0,06 | 0,61 | 0,50 | 0,77 | 0,65 | 62,62 |
| 14 | 102039726 | C | T | missense | 0,000003980 | 62/78 | 3895 | T/I | healthy | motor domain | 18,61 | 4,86 | 0,41 | 1,00 | 0,06 | 0,20 | 0,00 | 0,53 | 0,77 | 0,69 | 70,58 |
| 14 | 102039728 | G | A | missense | 0,000019900 | 62/78 | 3896 | V/M | healthy | motor domain | 8,32 | -7,95 | 0,39 | 1,00 | 0,12 | 0,14 | 0,06 | 0,53 | 0,76 | 0,70 | 72,37 |
| 14 | 102040255 | G | C | missense | 0,000003980 | 63/78 | 3904 | E/Q | healthy | motor domain | 24,80 | 5,82 | 0,50 | 1,00 | 0,44 | 0,13 | 0,93 | 0,65 | 0,75 | 0,87 | 93,74 |
| 14 | 102040266 | C | A | missense | 0,000003980 | 63/78 | 3907 | H/Q | healthy | motor domain | 22,50 | 3,99 | 0,41 | 1,00 | 0,32 | 0,30 | 0,73 | 0,68 | 0,74 | 0,92 | 95,98 |
| 14 | 102040264 | C | A | missense | 0,000007950 | 63/78 | 3907 | H/N | healthy | motor domain | 24,00 | 5,82 | 0,44 | 1,00 | 0,27 | 0,31 | 0,64 | 0,68 | 0,74 | 0,92 | 95,98 |
| 14 | 102040274 | G | C | missense | 0,000003980 | 63/78 | 3910 | R/T | healthy | motor domain | 23,70 | 5,82 | 0,46 | 1,00 | 0,38 | 0,35 | 0,03 | 0,72 | 0,75 | 0,97 | 97,50 |
| 14 | 102040287 | T | G | missense | 0,000031800 | 63/78 | 3914 | I/M | healthy | motor domain | 4,38 | -8,41 | 0,49 | 0,97 | 0,12 | 0,30 | 0,00 | NA | NA | NA | NA |
| 14 | 102040294 | A | C | missense | 0,000075600 | 63/78 | 3917 | S/R | healthy | motor domain | 22,90 | 5,82 | 0,00 | 0,94 | 0,07 | 0,15 | 0,04 | 0,77 | 0,75 | 1,20 | 98,64 |
| 14 | 102040300 | G | C | missense | 0,000003980 | 63/78 | 3919 | G/R | healthy | motor domain | 21,20 | 5,82 | 0,51 | 1,00 | 0,08 | 0,32 | 0,00 | 0,68 | 0,75 | 0,91 | 95,68 |
| 14 | 102040304 | C | T | missense | 0,000003980 | 63/78 | 3920 | S/F | healthy | motor domain | 20,00 | 4,75 | 0,48 | 1,00 | 0,10 | 0,03 | 0,01 | 0,70 | 0,75 | 0,92 | 96,06 |
| 14 | 102040307 | C | A | missense | 0,000007950 | 63/78 | 3921 | T/N | healthy | motor domain | 16,80 | 4,75 | 0,52 | 1,00 | 0,11 | 0,48 | 0,00 | 0,69 | 0,75 | 0,92 | 95,87 |
| 14 | 102040310 | C | T | missense | 0,000000000 | 63/78 | 3922 | P/L | healthy | motor domain | 22,20 | 5,82 | 0,00 | 1,00 | 0,37 | 0,06 | 0,00 | 0,69 | 0,75 | 0,93 | 96,21 |
| 14 | 102040319 | A | G | missense | 0,000011900 | 63/78 | 3925 | Q/R | healthy | motor domain | 17,81 | 3,41 | 0,00 | 0,63 | 0,06 | 0,51 | 0,00 | 0,67 | 0,75 | 0,89 | 94,73 |
| 14 | 102040330 | G | A | missense | 0,000003980 | 63/78 | 3929 | V/M | healthy | motor domain | 19,79 | 4,00 | 0,54 | 1,00 | 0,08 | 0,11 | 0,20 | 0,65 | 0,74 | 0,88 | 94,10 |
| 14 | 102040340 | C | T | missense | 0,000003980 | 63/78 | 3932 | A/V | healthy | motor domain | 21,40 | 5,82 | 0,58 | 0,64 | 0,05 | 0,29 | 0,00 | 0,65 | 0,74 | 0,87 | 93,54 |
| 14 | 102040345 | G | T | missense | 0,000003980 | 63/78 | 3934 | A/S | healthy | motor domain | 23,00 | 5,82 | 0,63 | 1,00 | 0,26 | 0,42 | 0,04 | 0,66 | 0,74 | 0,89 | 94,60 |
| 14 | 102040346 | C | T | missense | 0,000003980 | 63/78 | 3934 | A/V | healthy | motor domain | 23,40 | 4,88 | 0,69 | 1,00 | 0,21 | 0,20 | 0,45 | 0,66 | 0,74 | 0,89 | 94,60 |
| 14 | 102040348 | G | C | missense | 0,000003980 | 63/78 | 3935 | V/L | healthy | motor domain | 20,60 | 4,83 | 0,60 | 1,00 | 0,19 | 1,00 | 0,00 | 0,66 | 0,74 | 0,88 | 94,53 |
| 14 | 102040348 | G | A | missense | 0,000015900 | 63/78 | 3935 | V/M | healthy | motor domain | 21,90 | 4,83 | 0,64 | 1,00 | 0,14 | 0,50 | 0,01 | 0,66 | 0,74 | 0,88 | 94,53 |
| 14 | 102040351 | G | A | missense | 0,000011900 | 63/78 | 3936 | V/M | healthy | motor domain | 18,78 | 3,76 | 0,53 | 0,97 | 0,07 | 0,31 | 0,00 | 0,64 | 0,75 | 0,86 | 93,24 |
| 14 | 102040351 | G | T | missense | 0,000035800 | 63/78 | 3936 | V/L | healthy | motor domain | 20,30 | 3,76 | 0,54 | 0,98 | 0,07 | 0,16 | 0,01 | 0,64 | 0,75 | 0,86 | 93,24 |
| 14 | 102040366 | C | T | missense | 0,000007950 | 63/78 | 3941 | L/F | healthy | motor domain | 22,20 | 5,91 | 0,00 | 1,00 | 0,14 | 0,08 | 0,11 | 0,63 | 0,76 | 0,83 | 91,18 |
| 14 | 102040370 | C | A | missense | 0,000003980 | 63/78 | 3942 | P/H | healthy | motor domain | 23,80 | 5,03 | 0,50 | 1,00 | 0,23 | 0,02 | 0,06 | 0,63 | 0,76 | 0,83 | 90,64 |
| 14 | 102040370 | C | T | missense | 0,000003980 | 63/78 | 3942 | P/L | healthy | motor domain | 24,10 | 5,03 | 0,50 | 1,00 | 0,29 | 0,01 | 0,25 | 0,63 | 0,76 | 0,83 | 90,64 |
| 14 | 102040373 | C | A | missense | 0,000003980 | 63/78 | 3943 | A/E | healthy | motor domain | 23,80 | 5,91 | 0,58 | 1,00 | 0,32 | 0,13 | 0,88 | 0,62 | 0,77 | 0,81 | 88,98 |
| 14 | 102040372 | G | A | missense | 0,000007950 | 63/78 | 3943 | A/T | healthy | motor domain | 24,60 | 5,91 | 0,00 | 1,00 | 0,17 | 0,15 | 0,64 | 0,62 | 0,77 | 0,81 | 88,98 |
| 14 | 102040378 | A | C | missense | 0,000003980 | 63/78 | 3945 | K/Q | healthy | motor domain | 21,70 | 5,91 | 0,48 | 1,00 | 0,15 | 0,11 | 0,03 | 0,61 | 0,77 | 0,79 | 86,53 |
| 14 | 102040395 | G | T | missense | 0,000027800 | 63/78 | 3950 | K/N | healthy | motor domain | 22,70 | 4,85 | 0,54 | 1,00 | 0,12 | 0,42 | 0,23 | NA | NA | NA | NA |
| 14 | 102040403 | C | T | missense | 0,000003980 | 63/78 | 3953 | A/V | healthy | motor domain | 22,70 | 5,91 | 0,51 | 1,00 | 0,18 | 0,15 | 0,29 | 0,57 | 0,77 | 0,74 | 79,88 |
| 14 | 102040408 | G | A | missense | 0,000011900 | 63/78 | 3955 | E/K | healthy | motor domain | 24,00 | 4,93 | 0,51 | 1,00 | 0,26 | 0,29 | 0,02 | 0,59 | 0,78 | 0,76 | 82,40 |
| 14 | 102040605 | G | T | missense | 0,000063600 | 64/78 | 3958 | G/V | healthy | motor domain | 23,50 | 5,91 | 0,00 | 1,00 | 0,10 | 0,34 | 0,01 | 0,59 | 0,78 | 0,75 | 80,81 |
| 14 | 102040624 | C | A | missense | 0,000003980 | 64/78 | 3964 | S/R | healthy | motor domain | 22,00 | 4,07 | 0,66 | 1,00 | 0,12 | 0,30 | 0,07 | NA | NA | NA | NA |
| 14 | 102040629 | C | G | missense | 0,000000000 | 64/78 | 3966 | P/R | patient | motor domain | 27,20 | 5,91 | 0,62 | 1,00 | 0,68 | 0,00 | 0,99 | 0,56 | 0,77 | 0,72 | 75,43 |
| 14 | 102040631 | G | A | missense | 0,000003980 | 64/78 | 3967 | E/K | healthy | motor domain | 34,00 | 5,91 | 0,79 | 1,00 | 0,58 | 0,00 | 0,94 | 0,60 | 0,77 | 0,78 | 84,92 |
| 14 | 102040638 | C | A | missense | 0,000003980 | 64/78 | 3969 | T/N | healthy | motor domain | 22,10 | 5,91 | 0,65 | 1,00 | 0,13 | 0,39 | 0,00 | 0,58 | 0,77 | 0,75 | 80,91 |
| 14 | 102040656 | G | A | missense | 0,000003980 | 64/78 | 3975 | S/N | healthy | motor domain | 14,45 | 0,77 | 0,69 | 0,99 | 0,06 | 0,27 | 0,00 | 0,68 | 0,76 | 0,90 | 95,35 |
| 14 | 102040670 | G | T | missense | 0,000027800 | 64/78 | 3980 | A/S | healthy | motor domain | 16,47 | 1,67 | 0,67 | 0,60 | 0,08 | 0,75 | 0,00 | 0,71 | 0,75 | 0,94 | 96,97 |
| 14 | 102040673 | A | G | missense | 0,000007950 | 64/78 | 3981 | T/A | healthy | motor domain | 26,00 | 5,81 | 0,54 | 1,00 | 0,19 | 0,14 | 0,52 | 0,67 | 0,74 | 0,90 | 95,35 |
| 14 | 102041574 | C | G | missense | 0,000000000 | 65/78 | 3981 | T/R | healthy | motor domain | 26,70 | 5,42 | 0,00 | 1,00 | 0,24 | 0,10 | 0,92 | 0,67 | 0,74 | 0,90 | 95,35 |
| 14 | 102041576 | C | T | missense | 0,000003980 | 65/78 | 3982 | P/S | healthy | motor domain | 18,61 | 4,72 | 0,53 | 1,00 | 0,16 | 0,14 | 0,02 | 0,77 | 0,74 | 1,03 | 98,88 |
| 14 | 102041577 | C | G | missense | 0,000007960 | 65/78 | 3982 | P/R | healthy | motor domain | 23,90 | 5,61 | 0,56 | 1,00 | 0,17 | 0,04 | 0,01 | 0,77 | 0,74 | 1,03 | 98,88 |
| 14 | 102041594 | C | T | missense | 0,000003980 | 65/78 | 3988 | H/Y | healthy | motor domain | 22,30 | 5,61 | 0,72 | 1,00 | 0,23 | 0,60 | 0,00 | 0,82 | 0,75 | 1,10 | 99,61 |
| 14 | 102041594 | C | A | missense | 0,000003980 | 65/78 | 3988 | H/N | healthy | motor domain | 22,50 | 5,61 | 0,71 | 1,00 | 0,20 | 0,23 | 0,01 | 0,82 | 0,75 | 1,10 | 99,61 |
| 14 | 102041598 | G | A | missense | 0,000011900 | 65/78 | 3989 | R/H | healthy | motor domain | 24,10 | 4,72 | 0,00 | 1,00 | 0,21 | 0,13 | 0,00 | 0,82 | 0,74 | 1,11 | 99,63 |
| 14 | 102041597 | C | T | missense | 0,000015900 | 65/78 | 3989 | R/C | healthy | motor domain | 29,20 | 4,73 | 0,00 | 1,00 | 0,22 | 0,03 | 0,61 | 0,82 | 0,74 | 1,11 | 99,63 |
| 14 | 102041622 | G | A | missense | 0,000007960 | 65/78 | 3997 | R/Q | healthy | motor domain | 33,00 | 4,72 | 0,90 | 1,00 | 0,50 | 0,01 | 0,81 | 0,83 | 0,74 | 0,41 | 99,85 |
| 14 | 102041630 | C | T | missense | 0,000007950 | 65/78 | 4000 | R/C | healthy | motor domain | 33,00 | 5,61 | 0,87 | 1,00 | 0,67 | 0,01 | 1,00 | 0,84 | 0,74 | 1,13 | 99,89 |
| 14 | 102041637 | T | C | missense | 0,000019900 | 65/78 | 4002 | L/S | healthy | motor domain | 32,00 | 5,61 | 0,61 | 1,00 | 0,46 | 0,04 | 0,95 | NA | NA | NA | NA |
| 14 | 102041640 | C | T | missense | 0,000003980 | 65/78 | 4003 | A/V | healthy | motor domain | 23,60 | 4,73 | 0,63 | 1,00 | 0,24 | 0,16 | 0,18 | 0,86 | 0,76 | 1,13 | 99,81 |
| 14 | 102041645 | G | T | missense | 0,000003980 | 65/78 | 4005 | A/S | healthy | motor domain | 23,20 | 5,61 | 0,76 | 1,00 | 0,18 | 0,18 | 0,01 | 0,78 | 0,76 | 1,03 | 98,80 |
| 14 | 102041652 | T | C | missense | 0,000003980 | 65/78 | 4007 | M/T | healthy | motor domain | 20,30 | 2,00 | 0,68 | 0,86 | 0,04 | 0,69 | 0,00 | 0,78 | 0,76 | 1,03 | 98,86 |
| 14 | 102041651 | A | G | missense | 0,000007960 | 65/78 | 4007 | M/V | healthy | motor domain | 20,70 | -2,54 | 0,78 | 0,87 | 0,07 | 0,65 | 0,00 | 0,78 | 0,76 | 1,03 | 98,86 |
| 14 | 102041654 | T | C | missense | 0,000003980 | 65/78 | 4008 | F/L | healthy | motor domain | 24,10 | 5,61 | 0,82 | 1,00 | 0,29 | 0,28 | 0,05 | 0,78 | 0,75 | 1,03 | 98,92 |
| 14 | 102041663 | A | G | missense | 0,000051700 | 65/78 | 4011 | T/A | healthy | motor domain | 22,40 | 5,52 | 0,00 | 1,00 | 0,13 | 1,00 | 0,00 | 0,73 | 0,76 | 0,96 | 97,22 |
| 14 | 102041669 | C | T | missense | 0,000003980 | 65/78 | 4013 | L/F | healthy | motor domain | 21,50 | 5,52 | 0,81 | 1,00 | 0,22 | 1,00 | 0,01 | 0,75 | 0,76 | 0,98 | 97,72 |
| 14 | 102041672 | G | C | missense | 0,000003980 | 65/78 | 4014 | G/R | healthy | motor domain | 31,00 | 5,61 | 0,87 | 1,00 | 0,64 | 0,00 | 0,99 | 0,76 | 0,77 | 1,00 | 98,09 |
| 14 | 102041677 | G | T | missense | 0,000007960 | 65/78 | 4015 | E/D | healthy | motor domain | 18,00 | 1,68 | 0,51 | 1,00 | 0,15 | 0,51 | 0,00 | 0,77 | 0,76 | 1,02 | 98,60 |
| 14 | 102041679 | C | A | missense | 0,000007960 | 65/78 | 4016 | S/Y | healthy | motor domain | 23,40 | 5,61 | 0,72 | 1,00 | 0,18 | 0,01 | 0,15 | 0,76 | 0,77 | 1,00 | 98,09 |
| 14 | 102041679 | C | G | missense | 0,000015900 | 65/78 | 4016 | S/C | healthy | motor domain | 25,00 | 5,61 | 0,00 | 1,00 | 0,19 | 0,01 | 0,49 | 0,76 | 0,77 | 1,00 | 98,09 |
| 14 | 102041703 | C | T | missense | 0,000003980 | 65/78 | 4024 | P/L | healthy | motor domain | 22,90 | 5,61 | 0,73 | 1,00 | 0,22 | 0,28 | 0,03 | 0,73 | 0,78 | 0,94 | 96,75 |
| 14 | 102041708 | G | A | missense | 0,000011900 | 65/78 | 4026 | D/N | healthy | motor domain | 24,40 | 5,61 | 0,85 | 1,00 | 0,26 | 0,13 | 0,20 | 0,76 | 0,77 | 0,99 | 97,89 |
| 14 | 102041719 | C | A | missense | 0,000000000 | 65/78 | 4029 | H/Q | healthy | motor domain | 18,58 | 4,59 | 0,00 | 0,00 | 0,12 | 0,60 | 0,00 | NA | NA | NA | NA |
| 14 | 102041721 | T | C | missense | 0,000011900 | 65/78 | 4030 | I/T | healthy | motor domain | 23,90 | 5,61 | 0,83 | 1,00 | 0,27 | 0,26 | 0,16 | 0,72 | 0,76 | 0,95 | 97,10 |
| 14 | 102041727 | G | A | missense | 0,000003980 | 65/78 | 4032 | G/D | healthy | motor domain | 22,20 | 3,52 | 0,48 | 0,97 | 0,13 | 0,72 | 0,00 | 0,67 | 0,77 | 0,87 | 93,80 |
| 14 | 102041730 | C | T | missense | 0,000003980 | 65/78 | 4033 | T/I | healthy | motor domain | 23,00 | 4,73 | 0,58 | 1,00 | 0,09 | 0,07 | 0,17 | 0,69 | 0,76 | 0,91 | 95,42 |
| 14 | 102042014 | T | G | missense | 0,000003980 | 66/78 | 4035 | V/G | healthy | motor domain | 34,00 | 5,82 | 0,60 | 1,00 | 0,57 | 0,00 | 0,84 | 0,73 | 0,77 | 0,96 | 97,29 |
| 14 | 102042023 | A | G | missense | 0,000007950 | 66/78 | 4038 | N/S | healthy | motor domain | 22,60 | 5,91 | 0,40 | 1,00 | 0,09 | 1,00 | 0,00 | 0,69 | 0,77 | 0,90 | 95,14 |
| 14 | 102042026 | C | T | missense | 0,000003980 | 66/78 | 4039 | T/I | healthy | motor domain | 23,40 | 5,91 | 0,63 | 1,00 | 0,37 | 0,10 | 0,16 | 0,71 | 0,76 | 0,93 | 96,62 |
| 14 | 102042025 | A | G | missense | 0,000003980 | 66/78 | 4039 | T/A | healthy | motor domain | 23,70 | 5,91 | 0,61 | 1,00 | 0,30 | 0,14 | 0,11 | 0,71 | 0,76 | 0,93 | 96,62 |
| 14 | 102042052 | G | A | missense | 0,000003980 | 66/78 | 4048 | G/S | healthy | motor domain | 33,00 | 5,91 | 0,66 | 1,00 | 0,64 | 0,00 | 0,98 | 0,57 | 0,76 | 0,76 | 81,97 |
| 14 | 102042070 | C | T | missense | 0,000003980 | 66/78 | 4054 | H/Y | healthy | motor domain | 23,50 | 5,91 | 0,46 | 1,00 | 0,18 | 0,00 | 0,01 | 0,50 | 0,75 | 0,67 | 66,30 |
| 14 | 102042096 | G | C | missense | 0,000011900 | 66/78 | 4062 | Q/H | healthy | motor domain | 22,60 | 4,09 | 0,00 | 1,00 | 0,08 | 0,18 | 0,00 | 0,43 | 0,77 | 0,56 | 45,02 |
| 14 | 102042101 | C | T | missense | 0,000003980 | 66/78 | 4064 | T/M | healthy | motor domain | 22,70 | 5,03 | 0,44 | 1,00 | 0,08 | 0,09 | 0,03 | 0,39 | 0,76 | 0,51 | 35,79 |
| 14 | 102042105 | G | C | missense | 0,000003980 | 66/78 | 4065 | Q/H | healthy | motor domain | 22,90 | 5,03 | 0,56 | 1,00 | 0,12 | 0,11 | 0,03 | NA | NA | NA | NA |
| 14 | 102042240 | G | T | missense | 0,000000000 | 67/78 | 4076 | G/V | healthy | motor domain | 33,00 | 5,32 | 0,75 | 1,00 | 0,58 | 0,00 | 1,00 | 0,47 | 0,76 | 0,61 | 55,39 |
| 14 | 102042263 | A | G | missense | 0,000003980 | 67/78 | 4084 | I/V | healthy | motor domain | 23,50 | 3,38 | 0,78 | 1,00 | 0,27 | 0,07 | 0,46 | 0,40 | 0,75 | 0,53 | 40,35 |
| 14 | 102042272 | G | A | missense | 0,000003980 | 67/78 | 4087 | A/T | healthy | motor domain | 24,70 | 5,01 | 0,86 | 1,00 | 0,37 | 0,06 | 0,62 | 0,44 | 0,75 | 0,59 | 51,41 |
| 14 | 102042272 | G | T | missense | 0,000003980 | 67/78 | 4087 | A/S | healthy | motor domain | 28,20 | 5,01 | 0,84 | 1,00 | 0,43 | 0,00 | 0,94 | 0,44 | 0,75 | 0,59 | 51,41 |
| 14 | 102042443 | A | G | missense | 0,000003980 | 68/78 | 4112 | K/R | healthy | motor domain | 23,00 | 5,91 | 0,62 | 1,00 | 0,17 | 0,77 | 0,21 | 0,36 | 0,77 | 0,47 | 29,68 |
| 14 | 102042455 | T | A | missense | 0,000003980 | 68/78 | 4116 | L/Q | healthy | motor domain | 29,90 | 5,91 | 0,73 | 1,00 | 0,39 | 0,00 | 0,73 | 0,33 | 0,76 | 0,44 | 25,46 |
| 14 | 102042458 | A | G | missense | 0,000007960 | 68/78 | 4117 | Q/R | healthy | motor domain | 23,30 | 4,76 | 0,00 | 1,00 | 0,23 | 0,31 | 0,05 | 0,33 | 0,77 | 0,44 | 24,49 |
| 14 | 102042463 | C | A | missense | 0,000015900 | 68/78 | 4119 | H/N | healthy | motor domain | 24,50 | 5,03 | 0,00 | 1,00 | 0,26 | 0,17 | 0,68 | 0,31 | 0,75 | 0,41 | 20,87 |
| 14 | 102042647 | C | G | missense | 0,000003980 | 69/78 | 4138 | L/V | healthy | motor domain | 24,40 | 5,08 | 0,00 | 1,00 | 0,17 | 0,04 | 0,58 | NA | NA | NA | NA |
| 14 | 102042653 | C | T | missense | 0,000003980 | 69/78 | 4140 | R/C | healthy | motor domain | 29,80 | 6,07 | 0,73 | 1,00 | 0,42 | 0,00 | 1,00 | 0,50 | 0,74 | 0,67 | 67,83 |
| 14 | 102042654 | G | A | missense | 0,000015900 | 69/78 | 4140 | R/H | healthy | motor domain | 33,00 | 5,18 | 0,73 | 1,00 | 0,48 | 0,00 | 1,00 | 0,50 | 0,74 | 0,67 | 67,83 |
| 14 | 102042657 | C | T | missense | 0,000007960 | 69/78 | 4141 | A/V | healthy | motor domain | 22,40 | 6,07 | 0,56 | 1,00 | 0,29 | 0,09 | 0,13 | 0,50 | 0,75 | 0,67 | 66,86 |
| 14 | 102042662 | C | T | missense | 0,000003980 | 69/78 | 4143 | R/C | healthy | motor domain | 27,40 | 5,18 | 0,67 | 1,00 | 0,33 | 0,00 | 0,97 | 0,50 | 0,75 | 0,67 | 66,86 |
| 14 | 102042676 | C | G | missense | 0,000007960 | 69/78 | 4147 | F/L | healthy | motor domain | 17,55 | -9,11 | 0,62 | 1,00 | 0,33 | 0,09 | 0,62 | NA | NA | NA | NA |
| 14 | 102042677 | G | A | missense | 0,000003980 | 69/78 | 4148 | E/K | healthy | motor domain | 29,00 | 6,07 | 0,83 | 1,00 | 0,61 | 0,00 | 1,00 | NA | NA | NA | NA |
| 14 | 102042720 | G | T | missense | 0,000003980 | 69/78 | 4162 | S/I | healthy | motor domain | 26,00 | 6,07 | 0,00 | 1,00 | 0,39 | 0,03 | 0,11 | 0,53 | 0,75 | 0,70 | 72,52 |
| 14 | 102042731 | G | A | missense | 0,000023900 | 69/78 | 4166 | V/I | healthy | motor domain | 24,00 | 6,07 | 0,00 | 1,00 | 0,25 | 0,09 | 0,24 | 0,50 | 0,75 | 0,67 | 66,50 |
| 14 | 102042737 | C | T | missense | 0,000003980 | 69/78 | 4168 | R/W | healthy | motor domain | 27,50 | 2,88 | 0,00 | 1,00 | 0,36 | 0,00 | 0,97 | 0,53 | 0,75 | 0,71 | 74,09 |
| 14 | 102042738 | G | A | missense | 0,000007960 | 69/78 | 4168 | R/Q | healthy | motor domain | 34,00 | 6,07 | 0,57 | 1,00 | 0,32 | 0,04 | 0,84 | 0,53 | 0,75 | 0,71 | 74,09 |
| 14 | 102042747 | A | G | missense | 0,000011900 | 69/78 | 4171 | K/R | healthy | motor domain | 24,80 | 5,97 | 0,58 | 1,00 | 0,18 | 0,41 | 0,02 | 0,53 | 0,76 | 0,71 | 73,06 |
| 14 | 102043882 | A | G | missense | 0,000007950 | 70/78 | 4174 | N/S | healthy | motor domain | 22,40 | 6,07 | 0,41 | 1,00 | 0,08 | 0,64 | 0,01 | 0,44 | 0,76 | 0,58 | 50,03 |
| 14 | 102043891 | C | T | missense | 0,000003980 | 70/78 | 4177 | A/V | healthy | motor domain | 23,90 | 6,07 | 0,62 | 1,00 | 0,28 | 0,14 | 0,44 | 0,42 | 0,75 | 0,56 | 45,21 |
| 14 | 102043890 | G | A | missense | 0,000003980 | 70/78 | 4177 | A/T | healthy | motor domain | 24,10 | 6,07 | 0,00 | 1,00 | 0,28 | 0,36 | 0,04 | 0,42 | 0,75 | 0,56 | 45,21 |
| 14 | 102043942 | T | C | missense | 0,000007950 | 70/78 | 4194 | L/S | healthy | motor domain | 33,00 | 5,83 | 0,58 | 1,00 | 0,55 | 0,00 | 0,95 | 0,20 | 0,75 | 0,27 | 7,88 |
| 14 | 102043945 | G | A | missense | 0,000067600 | 70/78 | 4195 | R/Q | healthy | motor domain | 34,00 | 5,83 | 0,00 | 1,00 | 0,41 | 0,01 | 0,83 | 0,21 | 0,74 | 0,29 | 9,34 |
| 14 | 102043965 | T | A | missense | 0,000003980 | 70/78 | 4202 | S/T | healthy | motor domain | 23,40 | 5,83 | 0,63 | 1,00 | 0,19 | 0,35 | 0,33 | 0,24 | 0,75 | 0,32 | 11,51 |
| 14 | 102044005 | C | G | missense | 0,000003980 | 70/78 | 4215 | A/G | healthy | motor domain | 24,00 | 5,83 | 0,75 | 1,00 | 0,27 | 0,02 | 0,20 | 0,23 | 0,75 | 0,31 | 11,10 |
| 14 | 102044014 | C | T | missense | 0,000015900 | 70/78 | 4218 | T/M | healthy | motor domain | 23,60 | 5,83 | 0,67 | 1,00 | 0,35 | 0,10 | 0,69 | 0,25 | 0,76 | 0,33 | 13,00 |
| 14 | 102044280 | C | A | missense | 0,000003990 | 71/78 | 4231 | Q/K | healthy | tail domain | 24,70 | 5,56 | 0,64 | 1,00 | 0,11 | 0,00 | 0,05 | 0,19 | 0,75 | 0,25 | 5,75 |
| 14 | 102044311 | C | G | missense | 0,000003980 | 71/78 | 4241 | S/C | healthy | tail domain | 25,40 | 5,38 | 0,61 | 1,00 | 0,32 | 0,00 | 0,42 | 0,40 | 0,77 | 0,52 | 37,83 |
| 14 | 102044331 | G | T | missense | 0,000003980 | 71/78 | 4248 | A/S | healthy | tail domain | 22,50 | 4,48 | 0,72 | 1,00 | 0,25 | 1,00 | 0,01 | 0,37 | 0,77 | 0,48 | 31,18 |
| 14 | 102044334 | C | G | missense | 0,000035800 | 71/78 | 4249 | Q/E | healthy | tail domain | 22,50 | 5,38 | 0,71 | 1,00 | 0,29 | 0,59 | 0,44 | NA | NA | NA | NA |
| 14 | 102044340 | A | C | missense | 0,000003980 | 71/78 | 4251 | I/L | healthy | tail domain | 25,80 | 5,38 | 0,72 | 1,00 | 0,29 | 0,11 | 0,77 | 0,36 | 0,76 | 0,48 | 30,82 |
| 14 | 102044353 | G | A | missense | 0,000012000 | 71/78 | 4255 | R/H | healthy | tail domain | 33,00 | 5,37 | 0,89 | 1,00 | 0,35 | 0,01 | 0,98 | 0,43 | 0,75 | 0,57 | 47,17 |
| 14 | 102044355 | G | C | missense | 0,000007990 | 71/78 | 4256 | V/L | healthy | tail domain | 24,60 | 5,37 | 0,00 | 1,00 | 0,18 | 0,02 | 0,03 | 0,48 | 0,76 | 0,63 | 58,68 |
| 14 | 102044355 | G | A | missense | 0,000012000 | 71/78 | 4256 | V/M | healthy | tail domain | 25,00 | 5,37 | 0,72 | 1,00 | 0,26 | 0,00 | 0,12 | 0,48 | 0,76 | 0,63 | 58,68 |
| 14 | 102044386 | A | G | missense | 0,000007960 | 71/78 | 4266 | N/S | healthy | tail domain | 21,90 | 5,37 | 0,40 | 1,00 | 0,07 | 0,27 | 0,01 | 0,46 | 0,77 | 0,60 | 53,11 |
| 14 | 102044388 | A | G | missense | 0,000003980 | 71/78 | 4267 | T/A | healthy | tail domain | 22,70 | 5,37 | 0,66 | 1,00 | 0,21 | 0,46 | 0,01 | 0,46 | 0,78 | 0,60 | 52,64 |
| 14 | 102044401 | G | A | missense | 0,000007960 | 71/78 | 4271 | R/H | healthy | tail domain | 28,40 | 5,37 | 0,54 | 1,00 | 0,36 | 0,07 | 0,84 | 0,52 | 0,77 | 0,67 | 67,44 |
| 14 | 102044400 | C | T | missense | 0,000007960 | 71/78 | 4271 | R/C | healthy | tail domain | 33,00 | 4,48 | 0,61 | 1,00 | 0,45 | 0,01 | 0,91 | 0,52 | 0,77 | 0,67 | 67,44 |
| 14 | 102044408 | C | A | missense | 0,000003980 | 71/78 | 4273 | F/L | healthy | tail domain | 23,50 | 3,17 | 0,65 | 1,00 | 0,26 | 0,28 | 1,00 | 0,46 | 0,78 | 0,59 | 51,80 |
| 14 | 102044412 | A | C | missense | 0,000003980 | 71/78 | 4275 | T/P | healthy | tail domain | 22,40 | 5,37 | 0,54 | 0,66 | 0,29 | 1,00 | 0,00 | 0,52 | 0,79 | 0,66 | 65,51 |
| 14 | 102044413 | C | T | missense | 0,000007950 | 71/78 | 4275 | T/I | healthy | tail domain | 23,40 | 4,47 | 0,64 | 0,56 | 0,16 | 0,02 | 0,00 | 0,52 | 0,79 | 0,66 | 65,51 |
| 14 | 102044424 | G | A | missense | 0,000019900 | 71/78 | 4279 | D/N | healthy | tail domain | 24,20 | 4,47 | 0,00 | 1,00 | 0,23 | 0,38 | 0,04 | 0,50 | 0,79 | 0,63 | 59,26 |
| 14 | 102044429 | T | G | missense | 0,000003980 | 71/78 | 4280 | S/R | healthy | tail domain | 18,68 | -4,35 | 0,72 | 1,00 | 0,27 | 0,51 | 0,03 | NA | NA | NA | NA |
| 14 | 102044434 | T | G | missense | 0,000007950 | 71/78 | 4282 | F/C | healthy | tail domain | 34,00 | 5,37 | 0,70 | 1,00 | 0,70 | 0,00 | 0,99 | 0,58 | 0,77 | 0,75 | 80,01 |
| 14 | 102044442 | G | A | missense | 0,000003980 | 71/78 | 4285 | A/T | healthy | tail domain | 26,40 | 5,37 | 0,80 | 1,00 | 0,29 | 0,04 | 0,32 | 0,58 | 0,79 | 0,74 | 79,66 |
| 14 | 102044445 | T | C | missense | 0,000003980 | 71/78 | 4286 | C/R | healthy | tail domain | 23,00 | 2,86 | 0,56 | 1,00 | 0,30 | 0,43 | 0,00 | 0,56 | 0,79 | 0,71 | 74,18 |
| 14 | 102044454 | G | A | missense | 0,000011900 | 71/78 | 4289 | D/N | healthy | tail domain | 24,60 | 4,48 | 0,00 | 1,00 | 0,29 | 0,13 | 0,34 | 0,55 | 0,79 | 0,69 | 70,95 |
| 14 | 102044457 | G | A | missense | 0,000011900 | 71/78 | 4290 | G/R | healthy | tail domain | 33,00 | 5,37 | 0,55 | 1,00 | 0,39 | 0,01 | 0,78 | NA | NA | NA | NA |
| 14 | 102044472 | C | G | missense | 0,000011900 | 71/78 | 4295 | Q/E | healthy | tail domain | 22,50 | 5,37 | 0,63 | 1,00 | 0,20 | 0,97 | 0,00 | 0,60 | 0,80 | 0,75 | 81,26 |
| 14 | 102044477 | G | A | missense | 0,000007960 | 71/78 | 4296 | M/I | healthy | tail domain | 23,90 | 5,37 | 0,62 | 1,00 | 0,26 | 0,40 | 0,01 | 0,57 | 0,80 | 0,71 | 74,52 |
| 14 | 102044597 | G | A | missense | 0,000007950 | 72/78 | 4302 | R/Q | healthy | tail domain | 25,40 | 5,38 | 0,60 | 1,00 | 0,32 | 0,29 | 0,40 | 0,54 | 0,78 | 0,69 | 71,32 |
| 14 | 102044629 | C | T | missense | 0,000003980 | 72/78 | 4313 | P/S | healthy | tail domain | 23,10 | 5,38 | 0,68 | 1,00 | 0,32 | 0,08 | 0,12 | 0,30 | 0,76 | 0,40 | 19,20 |
| 14 | 102044642 | C | T | missense | 0,000011900 | 72/78 | 4317 | T/M | healthy | tail domain | 23,70 | 5,28 | 0,58 | 1,00 | 0,26 | 0,07 | 0,27 | 0,30 | 0,74 | 0,41 | 20,42 |
| 14 | 102044695 | C | A | missense | 0,000007960 | 72/78 | 4335 | Q/K | healthy | tail domain | 20,20 | 5,38 | 0,62 | 1,00 | 0,26 | 0,53 | 0,01 | 0,58 | 0,78 | 0,75 | 80,59 |
| 14 | 102047817 | G | C | missense | 0,000004000 | 73/78 | 4336 | G/A | healthy | tail domain | 24,50 | 5,37 | 0,64 | 1,00 | 0,36 | 0,25 | 0,38 | 0,58 | 0,78 | 0,75 | 80,59 |
| 14 | 102044698 | G | A | missense | 0,000011900 | 72/78 | 4336 | G/S | healthy | tail domain | 29,40 | 5,38 | 0,58 | 1,00 | 0,39 | 0,06 | 0,82 | 0,58 | 0,78 | 0,75 | 80,59 |
| 14 | 102047819 | G | A | missense | 0,000004000 | 73/78 | 4337 | V/M | healthy | tail domain | 23,10 | 4,36 | 0,61 | 0,99 | 0,15 | 0,10 | 0,17 | 0,64 | 0,78 | 0,82 | 90,04 |
| 14 | 102047834 | A | G | missense | 0,000003990 | 73/78 | 4342 | K/E | healthy | tail domain | 24,40 | 5,37 | 0,00 | 1,00 | 0,35 | 0,06 | 0,34 | NA | NA | NA | NA |
| 14 | 102047835 | A | G | missense | 0,000016000 | 73/78 | 4342 | K/R | healthy | tail domain | 25,60 | 5,37 | 0,58 | 1,00 | 0,18 | 0,12 | 0,52 | 0,50 | 0,79 | 0,63 | 59,26 |
| 14 | 102047852 | A | C | missense | 0,000003980 | 73/78 | 4348 | M/L | healthy | tail domain | 22,30 | 2,93 | 0,00 | 1,00 | 0,09 | 0,35 | 0,00 | 0,53 | 0,79 | 0,67 | 66,86 |
| 14 | 102047876 | G | C | missense | 0,000003980 | 73/78 | 4356 | A/P | healthy | tail domain | 25,60 | 4,48 | 0,50 | 1,00 | 0,18 | 0,07 | 0,95 | 0,43 | 0,79 | 0,54 | 41,36 |
| 14 | 102047882 | G | A | missense | 0,000031900 | 73/78 | 4358 | A/T | healthy | tail domain | 22,40 | 5,37 | 0,55 | 1,00 | 0,13 | 0,28 | 0,09 | 0,39 | 0,80 | 0,49 | 32,32 |
| 14 | 102047901 | C | T | missense | 0,000019900 | 73/78 | 4364 | T/M | healthy | tail domain | 19,23 | 5,37 | 0,50 | 1,00 | 0,09 | 0,10 | 0,01 | 0,35 | 0,77 | 0,46 | 27,98 |
| 14 | 102047913 | C | T | missense | 0,000003990 | 73/78 | 4368 | S/F | healthy | tail domain | 26,10 | 5,37 | 0,43 | 1,00 | 0,19 | 0,03 | 0,51 | 0,40 | 0,76 | 0,53 | 38,82 |
| 14 | 102047916 | C | T | missense | 0,000003990 | 73/78 | 4369 | T/M | healthy | tail domain | 22,60 | 4,45 | 0,33 | 1,00 | 0,14 | 0,09 | 0,56 | 0,44 | 0,76 | 0,59 | 50,61 |
| 14 | 102047921 | G | A | missense | 0,000007980 | 73/78 | 4371 | D/N | healthy | tail domain | 23,70 | 5,37 | 0,42 | 1,00 | 0,23 | 0,07 | 0,16 | 0,46 | 0,75 | 0,62 | 55,93 |
| 14 | 102047940 | T | C | missense | 0,000003990 | 73/78 | 4377 | M/T | healthy | tail domain | 27,60 | 5,37 | 0,63 | 1,00 | 0,40 | 0,00 | 0,98 | 0,50 | 0,75 | 0,67 | 67,03 |
| 14 | 102047955 | C | T | missense | 0,000004000 | 73/78 | 4382 | T/I | healthy | tail domain | 21,90 | 5,37 | 0,45 | 1,00 | 0,06 | 0,55 | 0,00 | 0,48 | 0,74 | 0,65 | 63,35 |
| 14 | 102047955 | C | A | missense | 0,000004000 | 73/78 | 4382 | T/N | healthy | tail domain | 22,80 | 5,37 | 0,53 | 1,00 | 0,10 | 0,16 | 0,12 | 0,48 | 0,74 | 0,65 | 63,35 |
| 14 | 102047954 | A | G | missense | 0,000004000 | 73/78 | 4382 | T/A | healthy | tail domain | 22,90 | 4,23 | 0,45 | 1,00 | 0,08 | 0,70 | 0,00 | 0,48 | 0,74 | 0,65 | 63,35 |
| 14 | 102047961 | C | T | missense | 0,000016000 | 73/78 | 4384 | A/V | healthy | tail domain | 23,30 | 5,37 | 0,00 | 1,00 | 0,16 | 0,32 | 0,16 | 0,48 | 0,74 | 0,65 | 63,07 |
| 14 | 102047960 | G | A | missense | 0,000020000 | 73/78 | 4384 | A/T | healthy | tail domain | 29,70 | 5,37 | 0,72 | 1,00 | 0,29 | 0,03 | 0,84 | 0,48 | 0,74 | 0,65 | 63,07 |
| 14 | 102047967 | A | G | missense | 0,000076000 | 73/78 | 4386 | N/S | healthy | tail domain | 23,20 | 5,37 | 0,51 | 1,00 | 0,08 | 0,50 | 0,00 | 0,44 | 0,75 | 0,60 | 52,40 |
| 14 | 102047971 | G | T | missense | 0,000000000 | 73/78 | 4387 | W/C | healthy | tail domain | 35,00 | 5,37 | 0,00 | 1,00 | 0,66 | 0,00 | 1,00 | 0,46 | 0,75 | 0,62 | 55,78 |
| 14 | 102047976 | A | G | missense | 0,000004010 | 73/78 | 4389 | H/R | healthy | tail domain | 23,00 | 4,21 | 0,44 | 0,99 | 0,10 | 0,41 | 0,00 | 0,46 | 0,75 | 0,62 | 56,34 |
| 14 | 102047991 | C | T | missense | 0,000000000 | 73/78 | 4394 | T/M | healthy | tail domain | 23,40 | 4,46 | 0,00 | 1,00 | 0,15 | 0,07 | 0,56 | 0,46 | 0,77 | 0,60 | 53,73 |
| 14 | 102048002 | C | T | missense | 0,000000000 | 73/78 | 4398 | L/F | healthy | tail domain | 27,40 | 5,37 | 0,00 | 1,00 | 0,24 | 0,05 | 0,98 | 0,41 | 0,76 | 0,54 | 40,65 |
| 14 | 102048014 | G | A | missense | 0,000016200 | 73/78 | 4402 | V/M | healthy | tail domain | 24,90 | 5,37 | 0,40 | 1,00 | 0,32 | 0,09 | 0,35 | 0,32 | 0,78 | 0,41 | 20,77 |
| 14 | 102048519 | C | A | missense | 0,000003980 | 74/78 | 4408 | P/T | healthy | tail domain | 26,40 | 5,25 | 0,78 | 1,00 | 0,75 | 0,00 | 1,00 | 0,38 | 0,77 | 0,49 | 33,74 |
| 14 | 102048558 | G | T | missense | 0,000007950 | 74/78 | 4421 | A/S | healthy | tail domain | 17,85 | 4,34 | 0,00 | 0,99 | 0,10 | 1,00 | 0,00 | 0,58 | 0,78 | 0,74 | 79,08 |
| 14 | 102048558 | G | A | missense | 0,000011900 | 74/78 | 4421 | A/T | healthy | tail domain | 20,90 | 4,34 | 0,51 | 1,00 | 0,15 | 0,36 | 0,01 | 0,58 | 0,78 | 0,74 | 79,08 |
| 14 | 102048563 | G | C | missense | 0,000003980 | 74/78 | 4422 | K/N | healthy | tail domain | 16,47 | -0,27 | 0,58 | 0,65 | 0,07 | 0,08 | 0,07 | 0,58 | 0,79 | 0,73 | 78,50 |
| 14 | 102048562 | A | C | missense | 0,000003980 | 74/78 | 4422 | K/T | healthy | tail domain | 18,65 | 1,68 | 0,62 | 0,97 | 0,23 | 0,11 | 0,24 | 0,58 | 0,79 | 0,73 | 78,50 |
| 14 | 102048576 | G | A | missense | 0,000011900 | 74/78 | 4427 | V/I | healthy | tail domain | 23,90 | 5,52 | 0,50 | 1,00 | 0,19 | 0,33 | 0,69 | 0,59 | 0,79 | 0,75 | 80,91 |
| 14 | 102048580 | G | A | missense | 0,000003980 | 74/78 | 4428 | R/H | healthy | tail domain | 24,10 | 5,52 | 0,67 | 1,00 | 0,26 | 0,07 | 0,06 | 0,61 | 0,79 | 0,77 | 84,38 |
| 14 | 102048579 | C | T | missense | 0,000007950 | 74/78 | 4428 | R/C | healthy | tail domain | 27,50 | 5,52 | 0,69 | 1,00 | 0,55 | 0,01 | 0,80 | 0,61 | 0,79 | 0,77 | 84,38 |
| 14 | 102048579 | C | G | missense | 0,000015900 | 74/78 | 4428 | R/G | healthy | tail domain | 31,00 | 5,52 | 0,00 | 1,00 | 0,43 | 0,00 | 0,94 | 0,61 | 0,79 | 0,77 | 84,38 |
| 14 | 102048583 | A | G | missense | 0,000003980 | 74/78 | 4429 | Q/R | healthy | tail domain | 22,20 | 5,52 | 0,48 | 1,00 | 0,22 | 0,83 | 0,00 | 0,59 | 0,79 | 0,75 | 80,05 |
| 14 | 102048583 | A | T | missense | 0,000011900 | 74/78 | 4429 | Q/L | healthy | tail domain | 23,30 | 5,52 | 0,49 | 1,00 | 0,23 | 0,26 | 0,00 | 0,59 | 0,79 | 0,75 | 80,05 |
| 14 | 102048594 | G | A | missense | 0,000003980 | 74/78 | 4433 | D/N | healthy | tail domain | 23,60 | 4,62 | 0,56 | 1,00 | 0,16 | 0,19 | 0,04 | 0,67 | 0,78 | 0,86 | 93,05 |
| 14 | 102048594 | G | T | missense | 0,000015900 | 74/78 | 4433 | D/Y | healthy | tail domain | 28,60 | 4,62 | 0,66 | 1,00 | 0,58 | 0,00 | 0,79 | 0,67 | 0,78 | 0,86 | 93,05 |
| 14 | 102048600 | G | A | missense | 0,000003980 | 74/78 | 4435 | V/I | healthy | tail domain | 22,00 | 4,62 | 0,50 | 1,00 | 0,11 | 0,68 | 0,01 | 0,63 | 0,77 | 0,82 | 89,82 |
| 14 | 102048601 | T | C | missense | 0,000019900 | 74/78 | 4435 | V/A | healthy | tail domain | 22,90 | 4,37 | 0,51 | 1,00 | 0,16 | 0,31 | 0,03 | 0,63 | 0,77 | 0,82 | 89,82 |
| 14 | 102048611 | C | G | missense | 0,000003980 | 74/78 | 4438 | C/W | healthy | tail domain | 22,60 | -2,54 | 0,68 | 1,00 | 0,42 | 0,00 | 1,00 | 0,59 | 0,76 | 0,77 | 83,58 |
| 14 | 102048612 | G | A | missense | 0,000000000 | 74/78 | 4439 | E/K | healthy | tail domain | 23,50 | 4,61 | 0,00 | 1,00 | 0,17 | 0,52 | 0,05 | 0,59 | 0,76 | 0,77 | 83,82 |
| 14 | 102048624 | A | C | missense | 0,000011900 | 74/78 | 4443 | K/Q | healthy | tail domain | 32,00 | 5,32 | 0,51 | 1,00 | 0,41 | 0,03 | 1,00 | 0,61 | 0,75 | 0,81 | 88,60 |
| 14 | 102048637 | A | G | missense | 0,000003980 | 74/78 | 4447 | Y/C | healthy | tail domain | 29,90 | 5,32 | 0,57 | 1,00 | 0,49 | 0,04 | 0,80 | 0,54 | 0,76 | 0,71 | 74,09 |
| 14 | 102048643 | G | A | missense | 0,000003980 | 74/78 | 4449 | R/H | healthy | tail domain | 32,00 | 5,32 | 0,65 | 1,00 | 0,53 | 0,00 | 1,00 | 0,52 | 0,76 | 0,69 | 70,09 |
| 14 | 102048646 | C | T | missense | 0,000000000 | 74/78 | 4450 | T/M | healthy | tail domain | 23,60 | 5,32 | 0,00 | 1,00 | 0,27 | 0,07 | 0,70 | 0,55 | 0,76 | 0,73 | 77,21 |
| 14 | 102048656 | C | G | missense | 0,000003980 | 74/78 | 4453 | N/K | healthy | tail domain | 12,73 | -1,49 | 0,53 | 1,00 | 0,12 | 0,07 | 0,09 | 0,52 | 0,76 | 0,69 | 69,81 |
| 14 | 102048667 | A | G | missense | 0,000003980 | 74/78 | 4457 | K/R | healthy | tail domain | 23,80 | 5,32 | 0,46 | 1,00 | 0,22 | 0,21 | 0,19 | 0,54 | 0,76 | 0,72 | 74,87 |
| 14 | 102049452 | G | A | missense | 0,000055700 | 75/78 | 4462 | R/Q | healthy | tail domain | 23,10 | 3,68 | 0,00 | 1,00 | 0,06 | 0,35 | 0,08 | 0,54 | 0,76 | 0,71 | 73,38 |
| 14 | 102049464 | A | T | missense | 0,000007960 | 75/78 | 4466 | H/L | healthy | tail domain | 23,30 | 5,54 | 0,49 | 1,00 | 0,14 | 0,08 | 0,00 | 0,55 | 0,76 | 0,72 | 75,90 |
| 14 | 102049466 | T | C | missense | 0,000003980 | 75/78 | 4467 | Y/H | healthy | tail domain | 32,00 | 5,54 | 0,58 | 1,00 | 0,33 | 0,00 | 1,00 | 0,58 | 0,76 | 0,76 | 82,25 |
| 14 | 102049470 | C | T | missense | 0,000007960 | 75/78 | 4468 | T/M | healthy | tail domain | 27,10 | 4,64 | 0,65 | 1,00 | 0,24 | 0,01 | 0,91 | 0,55 | 0,76 | 0,72 | 75,90 |
| 14 | 102049475 | C | T | missense | 0,000007960 | 75/78 | 4470 | P/S | healthy | tail domain | 26,80 | 5,54 | 0,00 | 1,00 | 0,39 | 0,03 | 0,97 | 0,56 | 0,76 | 0,73 | 77,51 |
| 14 | 102049481 | G | A | missense | 0,000007960 | 75/78 | 4472 | G/S | healthy | tail domain | 23,10 | 5,54 | 0,00 | 1,00 | 0,08 | 0,35 | 0,01 | 0,58 | 0,78 | 0,75 | 80,16 |
| 14 | 102049482 | G | T | missense | 0,000015900 | 75/78 | 4472 | G/V | healthy | tail domain | 23,60 | 5,54 | 0,55 | 1,00 | 0,18 | 0,08 | 0,10 | 0,58 | 0,78 | 0,75 | 80,16 |
| 14 | 102049486 | G | C | missense | 0,000003980 | 75/78 | 4473 | M/I | healthy | tail domain | 22,70 | 5,54 | 0,58 | 1,00 | 0,09 | 0,99 | 0,01 | 0,56 | 0,77 | 0,72 | 76,26 |
| 14 | 102049498 | G | T | missense | 0,000007960 | 75/78 | 4477 | Q/H | healthy | tail domain | 28,10 | 4,64 | 0,69 | 1,00 | 0,27 | 0,04 | 0,96 | NA | NA | NA | NA |
| 14 | 102049526 | A | G | missense | 0,000003980 | 75/78 | 4487 | K/E | healthy | tail domain | 23,80 | 4,39 | 0,48 | 1,00 | 0,25 | 0,32 | 0,22 | NA | NA | NA | NA |
| 14 | 102049576 | G | T | missense | 0,000003980 | 75/78 | 4503 | E/D | healthy | tail domain | 15,90 | 0,16 | 0,35 | 1,00 | 0,13 | 0,65 | 0,01 | 0,33 | 0,74 | 0,45 | 27,48 |
| 14 | 102049720 | C | T | missense | 0,000003990 | 76/78 | 4508 | H/Y | healthy | tail domain | 24,10 | 5,45 | 0,57 | 1,00 | 0,29 | 0,03 | 0,12 | 0,40 | 0,73 | 0,55 | 43,08 |
| 14 | 102049743 | C | G | missense | 0,000003990 | 76/78 | 4515 | F/L | healthy | tail domain | 14,84 | -3,86 | 0,88 | 1,00 | 0,28 | 0,31 | 0,13 | 0,40 | 0,74 | 0,54 | 41,21 |
| 14 | 102049744 | G | A | missense | 0,000003990 | 76/78 | 4516 | V/M | healthy | tail domain | 24,20 | 5,45 | 0,67 | 1,00 | 0,30 | 0,24 | 0,35 | 0,44 | 0,74 | 0,60 | 53,60 |
| 14 | 102049811 | A | C | missense | 0,000003990 | 76/78 | 4538 | E/A | healthy | tail domain | 26,80 | 5,35 | 0,57 | 1,00 | 0,35 | 0,06 | 0,56 | 0,57 | 0,76 | 0,76 | 81,82 |
| 14 | 102049823 | A | G | missense | 0,000012000 | 76/78 | 4542 | E/G | healthy | tail domain | 24,80 | 5,35 | 0,52 | 1,00 | 0,41 | 0,21 | 0,25 | 0,53 | 0,74 | 0,71 | 74,52 |
| 14 | 102049825 | G | A | missense | 0,000003990 | 76/78 | 4543 | V/I | healthy | tail domain | 23,60 | 5,35 | 0,52 | 1,00 | 0,25 | 0,23 | 0,06 | 0,50 | 0,75 | 0,67 | 67,59 |
| 14 | 102049829 | A | G | missense | 0,000003990 | 76/78 | 4544 | N/S | healthy | tail domain | 19,54 | 1,58 | 0,43 | 0,92 | 0,12 | 0,32 | 0,01 | 0,53 | 0,75 | 0,70 | 72,52 |
| 14 | 102049831 | G | A | missense | 0,000003990 | 76/78 | 4545 | V/I | healthy | tail domain | 21,50 | 5,35 | 0,61 | 1,00 | 0,18 | 0,83 | 0,04 | 0,53 | 0,75 | 0,70 | 72,52 |
| 14 | 102049841 | C | T | missense | 0,000016000 | 76/78 | 4548 | S/L | healthy | tail domain | 21,30 | 5,35 | 0,00 | 1,00 | 0,08 | 0,13 | 0,08 | NA | NA | NA | NA |
| 14 | 102049849 | G | A | missense | 0,000012000 | 76/78 | 4551 | A/T | healthy | tail domain | 16,00 | 1,51 | 0,00 | 1,00 | 0,09 | 0,56 | 0,02 | 0,57 | 0,75 | 0,76 | 82,89 |
| 14 | 102049850 | C | T | missense | 0,000032000 | 76/78 | 4551 | A/V | healthy | tail domain | 21,10 | 5,45 | 0,41 | 1,00 | 0,15 | 0,31 | 0,17 | 0,57 | 0,75 | 0,76 | 82,89 |
| 14 | 102049865 | G | A | missense | 0,000063900 | 76/78 | 4556 | C/Y | healthy | tail domain | 23,20 | 5,45 | 0,00 | 1,00 | 0,17 | 0,95 | 0,74 | 0,50 | 0,74 | 0,67 | 68,20 |
| 14 | 102049873 | G | A | missense | 0,000016000 | 76/78 | 4559 | G/R | healthy | tail domain | 23,30 | 5,45 | 0,64 | 1,00 | 0,38 | 0,53 | 0,02 | 0,48 | 0,74 | 0,65 | 62,77 |
| 14 | 102049880 | C | T | missense | 0,000003990 | 76/78 | 4561 | T/M | healthy | tail domain | 24,90 | 5,45 | 0,00 | 1,00 | 0,11 | 0,07 | 0,48 | 0,43 | 0,74 | 0,58 | 49,69 |
| 14 | 102050082 | C | G | missense | 0,000004010 | 77/78 | 4566 | Q/E | healthy | tail domain | 21,30 | 5,42 | 0,59 | 1,00 | 0,19 | 1,00 | 0,01 | 0,44 | 0,73 | 0,60 | 52,81 |
| 14 | 102050092 | C | T | missense | 0,000007950 | 77/78 | 4569 | T/M | healthy | tail domain | 22,80 | 4,53 | 0,00 | 1,00 | 0,06 | 0,10 | 0,18 | 0,42 | 0,73 | 0,58 | 48,96 |
| 14 | 102050104 | A | G | missense | 0,000071600 | 77/78 | 4573 | N/S | healthy | tail domain | 22,70 | 4,24 | 0,34 | 1,00 | 0,16 | 0,30 | 0,19 | 0,48 | 0,73 | 0,66 | 65,51 |
| 14 | 102050121 | A | G | missense | 0,000000000 | 77/78 | 4579 | N/D | healthy | tail domain | 15,78 | 3,05 | 0,50 | 0,94 | 0,09 | 1,00 | 0,00 | 0,48 | 0,73 | 0,66 | 65,59 |
| 14 | 102050127 | A | G | missense | 0,000003980 | 77/78 | 4581 | I/V | healthy | tail domain | 18,79 | 5,42 | 0,46 | 1,00 | 0,28 | 0,49 | 0,03 | 0,48 | 0,74 | 0,65 | 63,70 |
| 14 | 102050134 | C | G | missense | 0,000003980 | 77/78 | 4583 | T/S | healthy | tail domain | 18,44 | 5,42 | 0,36 | 1,00 | 0,16 | 0,33 | 0,15 | 0,46 | 0,74 | 0,62 | 56,94 |
| 14 | 102050136 | G | T | missense | 0,000007950 | 77/78 | 4584 | A/S | healthy | tail domain | 9,52 | 0,88 | 0,58 | 1,00 | 0,05 | 0,73 | 0,00 | 0,50 | 0,74 | 0,67 | 68,20 |
| 14 | 102050136 | G | A | missense | 0,000011900 | 77/78 | 4584 | A/T | healthy | tail domain | 11,87 | 0,88 | 0,00 | 1,00 | 0,06 | 0,58 | 0,00 | 0,50 | 0,74 | 0,67 | 68,20 |
| 14 | 102050139 | C | T | missense | 0,000003980 | 77/78 | 4585 | L/F | healthy | tail domain | 22,20 | 3,61 | 0,60 | 1,00 | 0,36 | 0,01 | 0,95 | 0,54 | 0,74 | 0,74 | 78,74 |
| 14 | 102050143 | C | T | missense | 0,000007950 | 77/78 | 4586 | P/L | healthy | tail domain | 24,40 | 5,42 | 0,82 | 1,00 | 0,50 | 0,01 | 0,52 | 0,60 | 0,74 | 0,81 | 89,54 |
| 14 | 102050149 | C | T | missense | 0,000043700 | 77/78 | 4588 | T/M | healthy | tail domain | 25,50 | 5,42 | 0,00 | 1,00 | 0,35 | 0,01 | 0,69 | 0,61 | 0,73 | 0,83 | 90,64 |
| 14 | 102050158 | G | A | missense | 0,000003980 | 77/78 | 4591 | R/H | healthy | tail domain | 25,10 | 5,42 | 0,55 | 1,00 | 0,28 | 0,04 | 0,06 | 0,65 | 0,73 | 0,90 | 95,05 |
| 14 | 102050157 | C | T | missense | 0,000007960 | 77/78 | 4591 | R/C | healthy | tail domain | 25,60 | 5,42 | 0,65 | 1,00 | 0,45 | 0,10 | 0,91 | 0,65 | 0,73 | 0,90 | 95,05 |
| 14 | 102050175 | A | G | missense | 0,000003980 | 77/78 | 4597 | N/D | healthy | tail domain | 12,87 | 5,42 | 0,32 | 1,00 | 0,11 | 1,00 | 0,00 | 0,58 | 0,73 | 0,79 | 86,36 |
| 14 | 102050181 | G | A | missense | 0,000007960 | 77/78 | 4599 | E/K | healthy | tail domain | 22,90 | 5,42 | 0,43 | 1,00 | 0,16 | 0,55 | 0,01 | NA | NA | NA | NA |
| 14 | 102050188 | A | C | missense | 0,000003980 | 77/78 | 4601 | K/T | healthy | tail domain | 19,62 | 5,42 | 0,44 | 1,00 | 0,25 | 0,58 | 0,00 | 0,54 | 0,74 | 0,74 | 79,08 |
| 14 | 102050187 | A | G | missense | 0,000007960 | 77/78 | 4601 | K/E | healthy | tail domain | 19,63 | 5,42 | 0,42 | 1,00 | 0,22 | 0,74 | 0,00 | 0,54 | 0,74 | 0,74 | 79,08 |
| 14 | 102050194 | G | T | missense | 0,000003980 | 77/78 | 4603 | S/I | healthy | tail domain | 12,81 | -4,05 | 0,00 | 1,00 | 0,07 | 0,12 | 0,02 | 0,50 | 0,74 | 0,68 | 68,43 |
| 14 | 102050193 | A | C | missense | 0,000000000 | 77/78 | 4603 | S/R | healthy | tail domain | 20,30 | 5,42 | 0,57 | 1,00 | 0,11 | 0,36 | 0,02 | 0,50 | 0,74 | 0,68 | 68,43 |
| 14 | 102050197 | T | C | missense | 0,000003980 | 77/78 | 4604 | V/A | healthy | tail domain | 19,48 | 5,51 | 0,64 | 1,00 | 0,14 | 0,51 | 0,01 | 0,54 | 0,75 | 0,73 | 77,38 |
| 14 | 102050463 | C | G | missense | 0,000003980 | 78/78 | 4614 | T/S | healthy | tail domain | 22,10 | 5,61 | 0,45 | 1,00 | 0,08 | 0,45 | 0,18 | 0,59 | 0,77 | 0,76 | 83,00 |
| 14 | 102050465 | C | T | missense | 0,000000000 | 78/78 | 4615 | R/C | healthy | tail domain | 27,80 | 4,70 | 0,88 | 1,00 | 0,65 | 0,00 | 1,00 | 0,55 | 0,77 | 0,72 | 75,43 |
| 14 | 102050477 | A | G | missense | 0,000003980 | 78/78 | 4619 | I/V | healthy | tail domain | 24,00 | 5,61 | 0,51 | 1,00 | 0,29 | 0,02 | 0,32 | 0,57 | 0,76 | 0,75 | 80,35 |
| 14 | 102050478 | T | C | missense | 0,000003980 | 78/78 | 4619 | I/T | healthy | tail domain | 27,50 | 5,61 | 0,50 | 1,00 | 0,34 | 0,00 | 0,76 | 0,57 | 0,76 | 0,75 | 80,35 |
| 14 | 102050486 | G | A | missense | 0,000007950 | 78/78 | 4622 | V/M | healthy | tail domain | 27,30 | 5,61 | 0,50 | 1,00 | 0,27 | 0,00 | 0,80 | 0,58 | 0,75 | 0,78 | 84,57 |
| 14 | 102050489 | G | A | missense | 0,000003980 | 78/78 | 4623 | D/N | healthy | tail domain | 26,50 | 5,61 | 0,00 | 1,00 | 0,22 | 0,11 | 0,78 | 0,61 | 0,75 | 0,82 | 89,82 |
| 14 | 102050494 | C | G | missense | 0,000003980 | 78/78 | 4624 | F/L | healthy | tail domain | 8,09 | -10,90 | 0,58 | 1,00 | 0,29 | 1,00 | 0,01 | NA | NA | NA | NA |
| 14 | 102050495 | G | A | missense | 0,000059600 | 78/78 | 4625 | E/K | healthy | tail domain | 23,60 | 5,61 | 0,00 | 1,00 | 0,22 | 0,80 | 0,00 | 0,61 | 0,75 | 0,82 | 89,82 |
| 14 | 102050498 | A | C | missense | 0,000003980 | 78/78 | 4626 | I/L | healthy | tail domain | 23,30 | 5,61 | 0,55 | 1,00 | 0,23 | 0,51 | 0,01 | 0,65 | 0,76 | 0,85 | 92,64 |
| 14 | 102050501 | G | A | missense | 0,000003980 | 78/78 | 4627 | A/T | healthy | tail domain | 24,00 | 5,61 | 0,37 | 1,00 | 0,19 | 0,40 | 0,02 | 0,67 | 0,76 | 0,88 | 94,34 |
| 14 | 102050507 | A | C | missense | 0,000003980 | 78/78 | 4629 | K/Q | healthy | tail domain | 23,50 | 5,61 | 0,41 | 1,00 | 0,20 | 0,53 | 0,01 | NA | NA | NA | NA |
| 14 | 102050516 | C | T | missense | 0,000003980 | 78/78 | 4632 | P/S | healthy | tail domain | 23,20 | 5,61 | 0,42 | 1,00 | 0,19 | 0,22 | 0,01 | 0,67 | 0,77 | 0,87 | 93,74 |
| 14 | 102050517 | C | G | missense | 0,000003980 | 78/78 | 4632 | P/R | healthy | tail domain | 23,60 | 5,61 | 0,48 | 1,00 | 0,31 | 0,07 | 0,01 | 0,67 | 0,77 | 0,87 | 93,74 |
| 14 | 102050520 | G | A | missense | 0,000011900 | 78/78 | 4633 | R/H | healthy | tail domain | 24,00 | 5,61 | 0,44 | 1,00 | 0,10 | 0,41 | 0,00 | 0,69 | 0,76 | 0,91 | 95,44 |
| 14 | 102050523 | G | T | missense | 0,000003980 | 78/78 | 4634 | S/I | healthy | tail domain | 23,60 | 5,61 | 0,51 | 1,00 | 0,19 | 0,29 | 0,01 | 0,69 | 0,76 | 0,91 | 95,57 |
| 14 | 102050527 | C | G | missense | 0,000003980 | 78/78 | 4635 | F/L | healthy | tail domain | 23,70 | 5,61 | 0,73 | 1,00 | 0,31 | 0,21 | 0,32 | 0,67 | 0,76 | 0,87 | 94,02 |
| 14 | 102050532 | A | G | missense | 0,000003980 | 78/78 | 4637 | E/G | healthy | tail domain | 24,70 | 5,61 | 0,52 | 1,00 | 0,34 | 0,06 | 0,09 | 0,67 | 0,77 | 0,87 | 93,48 |
| 14 | 102050531 | G | A | missense | 0,000003980 | 78/78 | 4637 | E/K | healthy | tail domain | 27,70 | 5,61 | 0,47 | 1,00 | 0,44 | 0,17 | 0,69 | 0,67 | 0,77 | 0,87 | 93,48 |
| 14 | 102050534 | C | T | missense | 0,000019900 | 78/78 | 4638 | R/W | healthy | tail domain | 33,00 | 5,61 | 0,76 | 1,00 | 0,72 | 0,00 | 1,00 | 0,68 | 0,77 | 0,89 | 94,71 |
| 14 | 102050543 | G | A | missense | 0,000007950 | 78/78 | 4641 | A/T | healthy | tail domain | 26,80 | 5,61 | 0,85 | 1,00 | 0,57 | 0,00 | 0,99 | 0,67 | 0,76 | 0,87 | 94,00 |

**Supplemental Table 2:** Raw data of Figure 1E and discussed domain-based statistical analyses with gnomAD allele frequencies, CADD-Phred scores, and MTR scores for *DYNC1H1* variants in the healthy population dataset (gnomAD v2.1), as well as likely pathogenic and pathogenic variants from the ClinVar database, and the pathogenic variants in our ten patients. We further report domains and pathogenicity scores for *DYNC1H1* variants (GERP RS, MutPred, MutationTaster, REVEL, SIFT, Polyphen, MTR observed (obs.), MTR expected (exp.), MTR score and MTR centile). AA, amino acid.
