# Supplementary material for: The clinical-phenotype continuum in DYNC1H1-related disorders—genomic profiling and proposal for a novel classification
Source: J Hum Genet. 2020 Aug 12;65(11):1003–17. doi: 10.1038/s10038-020-0803-1 (PMC7719554; doi:10.1038/s10038-020-0803-1)
Supplement: Supplementary file 3 — Supplementary Table 3 [file 10038_2020_803_MOESM3_ESM.docx]

**SUPPLEMENTARY MATERIAL**

**The clinical phenotype continuum in *DYNC1H1-*related disorders - genomic profiling and proposal for a novel classification**

**Supplementary Table 3**

| investigated clinical characteristics | no. of reported phenotypes | beginning tail domain | | dimerization domain | | linker | | motor domain | | Pearson*s Chi-Square Test | Lamdba symmetric | Phi | Cramer V |
| --- | --- | --- | --- | --- | --- | --- | --- | --- | --- | --- | --- | --- | --- |
|  |  | normal | abnormal | normal | abnormal | normal | abnormal | normal | abnormal |  |  |  |  |
| upper limb strength | 88 | 2 (33%) | 4 (67%) | 60 (88%) | 8 (12%) | 2 (100%) | 0 (0%) | 7 (58%) | 5 (42%) | 0.001 | ,054 | ,419 | ,419 |
| lower limb strength | 102 | 1 (17%) | 5 (83%) | 1 (1%) | 79 (99%) | 0 (0%) | 3 (100%) | 2 (15%) | 11 (85%) | 0.033 | 0.038 | 0.293 | 0.293 |
| intellectual disability | 83 | 0 (0%) | 6 (100%) | 40 (74%) | 14 (24%) | 0 (0%) | 4 (100%) | 5 (26%) | 14 (74%) | <0.0001 | 0.284 | 0.564 | 0.564 |
| behavioural abnormailites | 38 | 0 (0%) | 5 (100%) | 20 (69%) | 9 (31%) | 0 (0%) | 1 (100%) | 0 (0%) | 3 (100%) | 0.004 | 0.333 | 0.587 | 0.587 |
| seizures | 53 | 4 (80%) | 1 (20%) | 24 (89%) | 3 (11%) | 3 (75%) | 1 (25%) | 5 (30%) | 12 (71%) | 0.001 | 0.372 | 0.574 | 0.574 |
| MRI abnormalities | 70 | 1 (17%) | 5 (83%) | 27 (68%) | 13 (33%) | 0 (0%) | 4 (100%) | 0 (0%) | 20 (100%) | <0.0001 | 0.362 | 0.654 | 0.654 |
| pachygyria | 70 | 4 (67%) | 2 (34%) | 30 (75%) | 10 (25%) | 0 (0%) | 4 (100%) | 1 (5%) | 19 (95%) | <0.0001 | 0.477 | 0.664 | 0.664 |
| grey matter heterotopia | 70 | 6 (100%) | 0 (0%) | 40 (100%) | 0 (0%) | 4 (100%) | 0 (0%) | 12 (60%) | 8 (40%) | <0.0001 | 0.211 | 0.568 | 0.568 |
| enlarged ventricles | 70 | 3 (50%) | 3 (50%) | 37 (93%) | 3 (7%) | 4 (100%) | 0 (0%) | 17 (85%) | 3 (15%) | 0.028 | <0.0001 | 0.360 | 0.360 |
| hypoplasia corpus callosum | 70 | 5 (83%) | 3 (17%) | 34 (85%) | 6 (15%) | 4 (100%) | 0 (0%) | 11 (55%) | 9 (45%) | 0.041 | 0.065 | 0.344 | 0.344 |
| hypoplasia brain stem | 70 | 6 (100%) | 0 (0%) | 40 (100%) | 0 (0%) | 4 (100%) | 0 (0%) | 14 (70%) | 6 (30%) | 0.001 | 0.167 | 0.484 | 0.484 |
| hypoplasia cerebelllum | 70 | 6 (100%) | 0 (0%) | 38 (95%) | 2 (5%) | 4 (100%) | 0 (0%) | 12 (60%) | 8 (40%) | 0.002 | 0.150 | 0.467 | 0.467 |

**Supplementary Table 3:** Statistical analyses of the clinical characteristics of 130 patients, including 120 patients from the literature and ten from this study and mutations categorized into localization in DYNC1H1 (beginning tail: n=6, dimerization: n=85, linker: n=4, motor domain: n=25).
